# Scrub typhus ecology: a systematic review of *Orientia* in vectors and hosts

**DOI:** 10.1186/s13071-019-3751-x

**Published:** 2019-11-04

**Authors:** Ivo Elliott, Isabelle Pearson, Prabin Dahal, Nigel V. Thomas, Tamalee Roberts, Paul N. Newton

**Affiliations:** 1 Lao-Oxford-Mahosot Hospital-Wellcome Trust Research Unit, Microbiology Laboratory, Mahosot Hospital, Vientiane, Lao PDR; 2 Centre for Tropical Medicine and Global Health, Nuffield Department of Medicine, University of Oxford, Oxford, UK; 3 Worldwide Anti Malarial Resistance Network, University of Oxford, Oxford, UK; 4 Linacre College, University of Oxford, St Cross Road, Oxford, UK

**Keywords:** Scrub typhus, *Orientia tsutsugamushi*, Chigger, Trombiculid, Ecology, Vector, Host

## Abstract

Scrub typhus, caused by *Orientia tsutsugamushi*, is an important and neglected vector-borne zoonotic disease with an expanding known distribution. The ecology of the disease is complex and poorly understood, impairing discussion of public health interventions. To highlight what we know and the themes of our ignorance, we conducted a systematic review of all studies investigating the pathogen in vectors and non-human hosts. A total of 276 articles in 7 languages were included, with 793 study sites across 30 countries. There was no time restriction for article inclusion, with the oldest published in 1924. Seventy-six potential vector species and 234 vertebrate host species were tested, accounting for over one million trombiculid mites (‘chiggers’) and 83,000 vertebrates. The proportion of *O. tsutsugamushi* positivity was recorded for different categories of laboratory test and host species. Vector and host collection sites were geocoded and mapped. Ecological data associated with these sites were summarised. A further 145 articles encompassing general themes of scrub typhus ecology were reviewed. These topics range from the life-cycle to transmission, habitats, seasonality and human risks. Important gaps in our understanding are highlighted together with possible tools to begin to unravel these. Many of the data reported are highly variable and inconsistent and minimum data reporting standards are proposed. With more recent reports of human *Orientia* sp. infection in the Middle East and South America and enormous advances in research technology over recent decades, this comprehensive review provides a detailed summary of work investigating this pathogen in vectors and non-human hosts and updates current understanding of the complex ecology of scrub typhus. A better understanding of scrub typhus ecology has important relevance to ongoing research into improving diagnostics, developing vaccines and identifying useful public health interventions to reduce the burden of the disease.
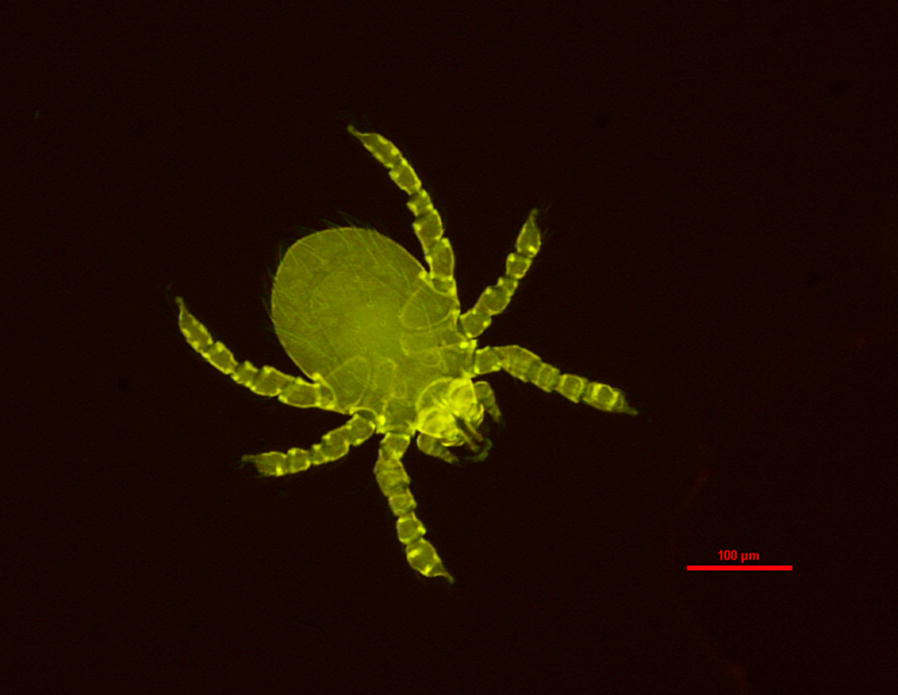

## Background

Scrub typhus is a vector-borne zoonotic disease with the potential of causing life-threatening febrile infection in humans. The disease is caused by a Gram-negative bacterium, *Orientia tsutsugamushi*, and is transmitted by the larval stage of mites (“chiggers”) in the family Trombiculidae. Scrub typhus has an expanding known distribution, with most disease occurring across South and East Asia and parts of the Pacific Rim [1]. The infection may be acquired in both rural and semi-urban environments and thus an enormous human population is likely to be at risk. In some areas of Southeast Asia, scrub typhus causes up to 23% of febrile hospital admissions [2–5]. In prospective studies in six countries across Asia, seroprevalence ranged from 9.3 to 27.9% with a median of 22.2% [6]. Minimum disease incidence reported by countries with passive national surveillance systems has shown an apparent rise with a median of 4.6/100,000/10 years [6]. Mortality rates range widely with a median of 6% untreated, falling to 1.4% for treated cases [7]. A single study from eastern China has calculated disability adjusted life years (DALYs), estimating a rate of 1.06/100,000 [8]. The ecology of scrub typhus covers a multitude of topics from the life-cycle and biology of the vector, to its interaction with hosts and the environment. Much of our existing knowledge of these topics is based on research carried out during World War II and until the 1970s. Traub & Wisseman [9] published the last comprehensive review on the subject in 1974. Scientific and technological advances now provide the opportunity to revisit many of these critical topics. Important gaps in knowledge include fundamental aspects of the biology, life-cycle and effects of *Orientia* on the vector; taxonomy and geographical variation of vectors; the scale, heterogeneity and dynamics of high-risk areas and the key factors that influence human infection risk. These many gaps in our knowledge act as barriers to our ability to make breakthroughs in diagnostics and vaccine development and ultimately public health interventions to reduce the burden of the disease in poor rural communities across Asia and potentially further afield.

Data were systematically reviewed from all accessible articles using aetiological diagnostic tests to identify *Orientia* sp. infection in vectors and non-human hosts and the location of these studies mapped. The major themes in the ecology of the disease are then reviewed. The relationship between human infection and disease ecology is examined and the limitations of the existing literature are discussed and minimum reporting criteria proposed. Finally, the key gaps in our understanding are reviewed and available tools identified to begin to unravel the details of this complex tropical disease.

## Methods

### Eligibility criteria

Articles were selected with two separate aims. First, all articles using any aetiological laboratory test to detect *Orientia* sp. infection in any potential vector or non-human animal vertebrate host were included. Secondly, any article not included in the first selection, but containing information broadly encompassing the term “ecology” was reviewed. In this review the term “ecology” describes vector-host-pathogen interactions in the context of their environment and evolution. Although the focus of the systematic review is on non-human hosts, the review of ecology includes detailed human interactions. There were no restrictions based on year of publication or language.

### Information sources

Articles were identified through electronic resources and by scanning reference lists of relevant articles. The electronic search was performed using Embase (1974-present), Medline (1950-present), CAB Abstracts (1910-present) and Web of Science (1900-present). Additionally, an unpublished list of scrub typhus articles produced by Michael W. Hastriter in 2012 was scanned for relevant articles (previously, but no longer, accessible at: http://www.afpmb.org/sites/default/files/whatsnew/2012/Hastriter_Complete.pdf). The first search took place on 26th October 2015 with regular updates using the same search terms until 20th November 2018.

### Search strategy

The electronic databases were searched using the following terms: scrub typhus or *Orientia tsutsugamushi* or *Rickettsia tsutsugamushi* or *O tsutsugamushi* or Orientia tsu* or akamushi disease or Japanese river fever or Nippon river fever or mite typhus or mite-borne typhus or tropical typhus or tsutsugamushi disease or Kedani fever or akamushi or shimamushi or shichito fever or XK typhus. These terms were combined with at least 1 of the following terms: ! mite* or chigger* or trombicul* or Leptotromb* or rodent* or rats or mammal* or animal* or ecolog* or epidemiolog* or vector* or ‘natural history’. Duplicate search results were removed using Endnote X7. Articles were searched in all languages. No unpublished literature or conference abstracts were included.

This review followed the PRISMA statement for systematic reviews (Additional file 1: Table S1). The review was not eligible for registration with the international prospective register of systematic reviews (PROSPERO) as it does not have a health-related outcome directly relevant to human health.

### Study selection

The first author reviewed titles and abstracts for all articles for inclusion. If there was any doubt regarding inclusion, then the full article was obtained for assessment. A native speaker in collaboration with the author reviewed articles in languages other than English, French, German or Dutch for inclusion.

### Data extraction

For articles in English, the first author and two other investigators extracted data for year of study, dates of sample collection, location, host and vector species collected, numbers tested and numbers positive, whether samples were pooled or tested individually, sample type, vector infestation rate and index, vector collection method, habitat description, rainfall during study, minimum, maximum and mean temperature during study and laboratory test used. For Chinese, Japanese, Russian, Korean and Thai languages a native speaker extracted data using the same template, crosschecking with the author for consistency. Data was entered into a pre-designed Microsoft Access database.

### Planned analysis

#### Descriptive summaries

The location and accuracy of each study site was recorded. All laboratory tests used to identify *O. tsutsugamushi* were noted and classified into 8 groups: molecular, serological, combined molecular and serological, culture, culture with serology, culture with molecular, microscopy alone and unknown (Additional file 1: Table S2). The distribution of key vector species is described together with all reported vector species. Key themes in the ecology of scrub typhus are reviewed in detail. The risk of bias was high due to many missing data, particularly denominator values for number of tested vectors and hosts.

#### Statistics

The primary outcome of the systematic review was the median (range) positivity of *O. tsutsugamushi* in diverse mites and other Acari and vertebrates. Analysis was performed using Stata v.15 (StataCorp, College Station, TX, USA) and R statistical software (R Core Development Team, 2018).

### Geocoding

All study sites were geocoded with the aim of creating a single location for each site. Where exact coordinates were provided, these were used. For all other locations, the “Geocode csv with Google/Open street map”, MMQGIS plugin for QGIS was used to geocode sites (QGIS Development Team, 2018; Geographic Information System, Open Source Geospatial Foundation Project; http://qgis.osgeo.org). Any available combination of address, city, state, province and country was entered. Where geocoding failed, several solutions were explored. First, the site was searched for on the internet using numerous resources and then geocoded manually using Google Maps. Secondly, spelling variation of place names (e.g. for Korean sites) was frequently inconsistent with Google Maps, and variations were tried with input from a native speaker where possible. Finally, if no location could be found, then the next administrative level up was selected by using the geocoding method above.

A number of additional situations arose: (i) where samples were collected from multiple locations and pooled such that it could not be determined from where the samples originated, the mean latitude and longitude of these sites was used to generate a single point; (ii) where samples were collected from multiple contiguous administrative areas, these areas were combined in QGIS using the ‘Vector-Geoprocessing-Dissolve’ function and a polygon centroid generated to create a single point for the study site; (iii) where samples originated from multiple non-contiguous sites, these areas were selected using QGIS and converted from singlepart to multipart and then a centroid created to give a single point; in the latter case the point could be outside the actual administrative zones where the study took place.

All study sites were classified into 1 of 6 administrative divisions (Additional file 1: Table S3). Administrative levels 1 to 4 were based on those listed in the International Organization for Standardization codes ISO 3166-1 and ISO 3166-2 [10]. Where further detail for a particular country was required, this information was obtained through the country’s Wikipedia page for administrative divisions. Some extinct historical administrative divisions were encountered, and here the closest match or next administrative level up was selected. Two additional administrative divisions were included: Level 0 for the country alone and level 0.5 for a well-defined region of a country, e.g. Peninsular Malaysia, Kyushu or Kanto regions of Japan.

## Results

### Study characteristics

**Fig. 1:**
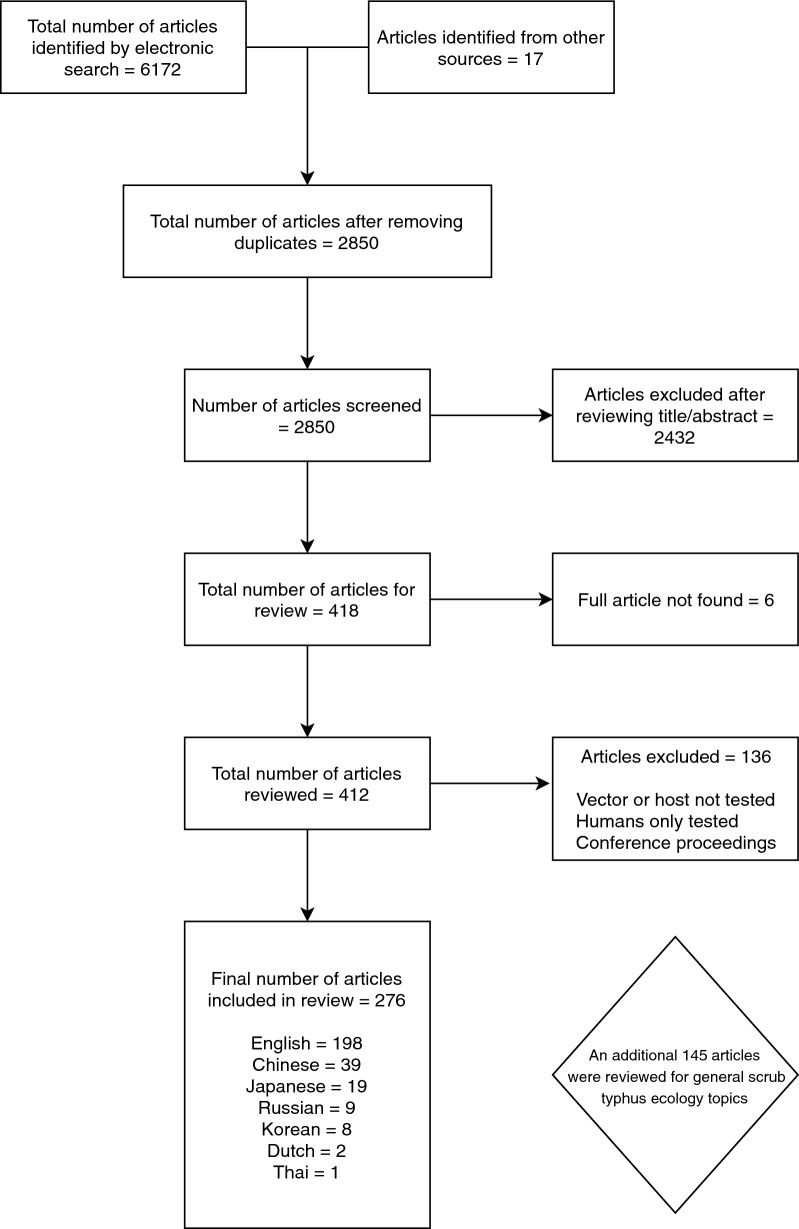
Study selection strategy flowchart

**Fig. 2:**
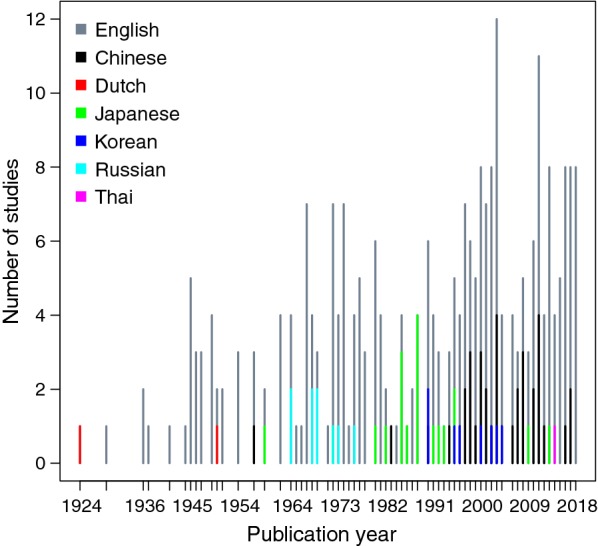
Number of included studies published in different languages over time

A total of 276 articles were included in the systematic review, with a further 145 reviewed for information on a general discussion of scrub typhus ecology. Only 6 articles were excluded, as the full text could not be obtained (Fig. 1). Systematic review articles were published between 1924 and 2018, and other articles reviewed dated back to 1878. Systematic review papers included 198 in English, 39 in Standard Chinese (Mandarin), 19 in Japanese, 9 in Russian, 8 in Korean, 2 in Dutch and 1 in Thai (Fig. 2).

The number of published articles has gradually increased over time. Some of the earliest work was published in Dutch, reflecting the Dutch presence in the East Indies (now Indonesia). From the mid-1960s to the mid-1970s there were a number of papers in Russian. There has been little published investigation in Russia since then. Articles in Japanese were more frequent in the 1980s, followed by Korean in the 1990s to 2000s. Articles from these two countries are now mostly published in English. A surge in Mandarin Chinese articles is seen from the mid-1990s.

### Geography

Historically scrub typhus was thought to be present across a large swathe of South and East Asia, known as the “tsutsugamushi triangle”. In vectors and non-human hosts, the pathogen has been identified from as far north as the Russian Kuril Islands, north of Japan and Inner Mongolia (~ 49°N) [11, 12]. The most easterly record comes from the Eastern Solomon Islands (~ 167°E) [13]. To the south there is evidence from North Queensland, Australia (~ 21°S) and in the west from eastern Iran (~ 59°E) [14, 15]. In 1946, Baker [16] published a study suggesting a rickettsial species consistent with scrub typhus that was detected in Canadian voles trapped on Grosse Isle in the St. Lawrence River near Quebec City. More recently two studies using *16S* rRNA sequencing of blood samples from rodents in the Ardennes, France and in Senegal and from dogs near Kruger National Park, South Africa, identified organisms with close sequence homology to *O. tsutsugamushi* [17, 18]. In 2006, serological evidence of human scrub typhus was reported from Chiloe Island in Chile [19]. In 2016, molecular testing confirmed further cases [20] and then in 2018, serological evidence of *O. tsutsugamushi* was demonstrated in dogs on Chiloe Island (~ 42°S) [21]. In the same year, an organism with close homology to *O. chuto* was detected in pooled *Microtrombicula* and *Neotrombicula* species chiggers in Baringo County, Kenya [22]. This follows the identification of a human case of *O. chuto* infection in the United Arab Emirates in 2010 [23]*. Orientia tsutsugamushi* has been identified from as high as 3200 m above sea level in the Kaghan Valley of Pakistan [24].

The disease, in *sensu lato*, has an expanding known geographical distribution and much remains to be understood of its distribution across tropical and subtropical regions, its presence in vectors and hosts and role in causing human disease.

#### Studies sites per country

**Fig. 3:**
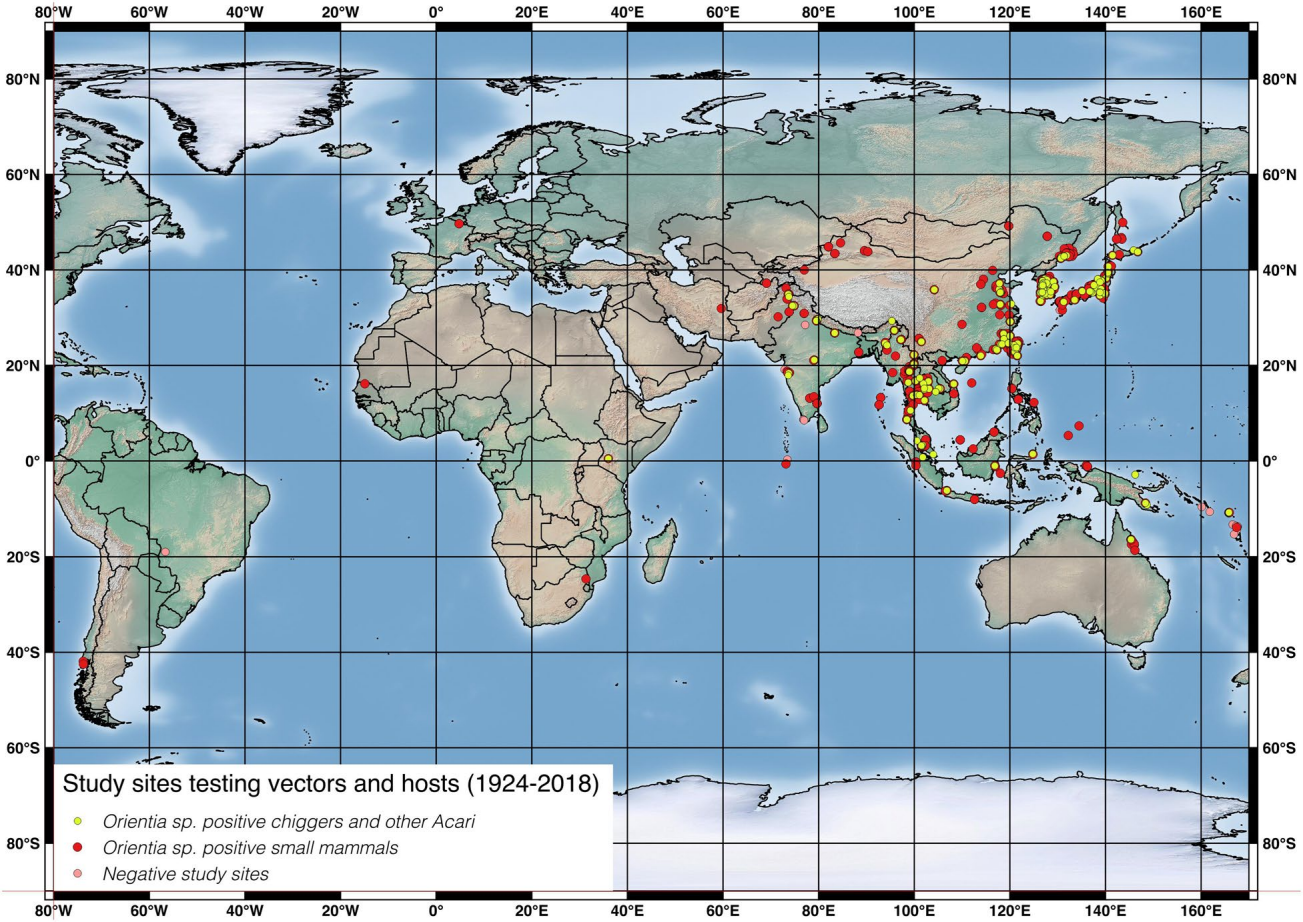
Location of study sites investigating *O. tsutsugamushi* in vectors and hosts. One study identifying *O. tsutsugamushi*-like organisms in small mammals in Quebec Province, Canada in 1946 is omitted here [16]

Articles included in this review span publication across 94 years. Studies for which laboratory tests were performed on vectors and non-human hosts to identify *O. tsutsugamushi* took place at 793 sites in 30 countries (Additional file 1: Table S3). South Korea and Japan had by far the most study sites recorded at 183 and 144, respectively (accounting for 42.1% of all study sites). Thailand had 87 sites, China 66, Taiwan 63, Russia 53, India 44 and Malaysia 43. Thirteen countries had 3 or less study sites (Fig. 3).

It is worth noting that individual studies varied enormously in the number of collection sites, with some having just 1 and others 30 or more. Additionally, more study sites than those reported here exist in practice, but where data could not be separated by site some were amalgamated following the strategy described above.

Apart from an early inconclusive investigation in Canadian voles by Baker in 1946 [16], it is only since 2015 that investigations into *Orientia* infection in vectors and hosts have taken place outside the Asia–Pacific region.

#### Negative study sites

In total, 53/793 (6.7%) study sites reported no positive vectors or hosts. These were located in 12 countries across the Asia–Pacific region and Brazil (Fig. 3). Twenty-one of these sites were from a single study of ports and harbours in the Republic of China (hereafter Taiwan) [25].

#### Geocoding accuracy

Only 12 out of 793 (1.5%) sites were geocoded to administrative level 0 (corresponding to an unknown point in the country) and a further 12 sites to administrative level 0.5. A total of 100 out of 793 (13%) sites were geocoded at level 1, 124 (16%) sites at level 2, 209 (26%) sites at level 3 and 336 (42%) were geocoded most accurately at level 4 (either an exact site was provided or the village or equivalent given). On the other hand, 456 out of 793 sites (58%) were geocoded at administrative level 3 or less, indicating that the majority of reported sites were no more accurate than the district or equivalent level.

### Laboratory tests and sample types

Given the 94-year period from which included studies were drawn and the many countries in which studies were performed, it is unsurprising that a large range of laboratory tests and combinations of tests were used to test both vectors and hosts. More than 40 tests and combinations of tests were recorded (Additional file 1: Table S2). These include some broad categories (serology, antigen tests and molecular tests) for which further details were not provided. Four studies did not clearly state the laboratory test used. Two of these were review articles that contained data not published elsewhere [26, 27], one was a short report [28] and the fourth paper was on studies of transovarial transmission in chiggers collected from the wild [29].

To aid analysis, these tests were grouped into 8 categories (Additional file 1: Table S2). Serological tests [which include direct and indirect immunofluorescence (DIF, IIF), indirect immunoperoxidase (IIP), complement fixation (CF), enzyme-linked immunosorbent assays (ELISA) and the Weil–Felix (OXK) test] were performed in 121/276 (44%) articles. Next most commonly used was culture (with or without microscopic confirmation) with 72/276 (26%) articles. All but one of these used the xenodiagnosis method of animal inoculation and passage. Only 3 such studies were reported since the start of the 21st century. Molecular methods were used in 63 articles from 14 countries. The first of these was published in 1995. A range of *O. tsutsugamushi* PCR targets were used including 47 and 56 kDa, GroEL, OmpB, in-house targets and nested PCRs. Two of the studies used *16S* rRNA sequencing [17, 18]. Microscopy alone (“organ impression smears”) was performed in 3 studies, 2 prior to 1950 and 1 from India in 2012 [30–32].

**Fig. 4:**
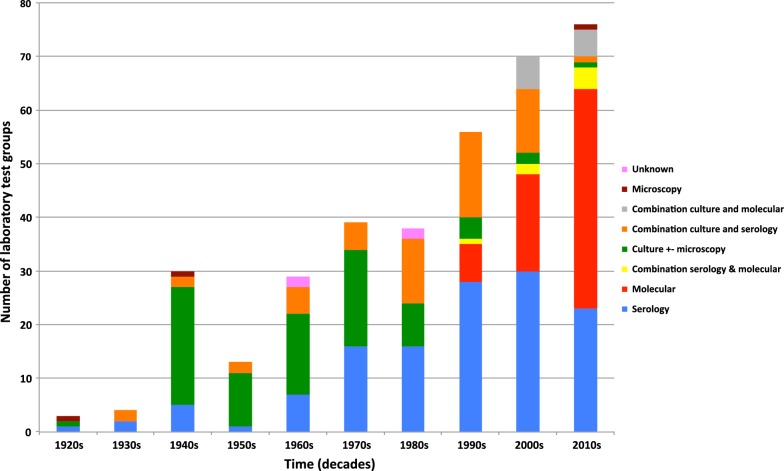
Use of different categories of laboratory test over time

Combinations of tests were used in 71/276 (26%) studies. Fifty-three studies used a combination of culture (xenodiagnosis) and one of several serological tests. Less frequently, 11 (4%) studies (all published since 2000) reported combinations of culture and molecular diagnosis. Four of these studies used L929 cell culture [33–36]. Seven studies used a combination of molecular (PCR) and serological (ELISA or IIF) tests to report *O. tsutsugamushi* testing of vectors and hosts. Figure 4 shows the use of test groups over time.

Different sample types were used from host animals. Reflecting the frequency of serological studies, 99/276 (36%) articles used serum specimens. Whole blood was analysed in 23/276 (8%) studies. Single organ types were tested in 69/276 (25%) studies, with spleen predominating in 62 studies, kidney in 2 and brain in 5 (from the 1940s). A high proportion of studies 91/276 (33%) used a combination of tissues (spleen, liver, kidney, lung, brain and whole blood). In only 3 studies was the specimen not recorded, 2 of these were review articles [26, 27] and the other Audy’s War Office report [31].

### Vectors

#### Orientia tsutsugamushi testing of individual and pooled vectors collected from hosts

A total of at least 74 “vector” species were tested for *O. tsutsugamushi* using a laboratory test. Sixty of these were trombiculid mites and the rest were other members of the Acari: Ixodida, Laelapidae or Macronyssidae. Of the Trombiculidae, 46 species tested positive for *O. tsutsugamushi* at least once (Additional file 1: Table S4). Vectors were tested either individually or as pools of individuals. Pool size varied enormously, from less than 10 to over 1000 chiggers. A total of over 123,000 individuals and 8000 pools (accounting for over 1 million chiggers) were tested.

In some studies, the species were listed but details on the numbers tested (denominator) of each were not specified. In other studies, the overall number of different species tested were listed, but the data was not divided between two or more collection sites. In these cases, the species was reported as “not identified’ in favour of the location which was deemed a more useful data record. For pools of vectors, many were of mixed species or unidentified, and in many studies vectors were identified only to genus rather than species.

Percentage infection rates among individual trombiculid mites range from 0.6 to 5% depending on the laboratory tests employed (Table 1). The highest rates of infection were observed using immunofluorescence techniques that are sensitive but can suffer from false positives. Culture is likely to be specific but may lack sensitivity. Molecular methods gave an overall infection rate of 1.9%. Pools of vectors gave expectedly higher positivity rates ranging from 9.6 to 56% (Table 1). The highest rate was seen for combined molecular and serological methods, although only a small number of pools were tested in this manner. Molecular techniques for pooled vectors gave a positivity rate of 31% and, surprisingly, using serological tests, only 11% were reported positive.

**Table 1:** Summary of number of tested “vectors” and number of positives of all species combined. Data is divided into individual vectors or pooled (multiple) individuals and subdivided by laboratory test category

Laboratory method	Total no. of individuals tested	Total no. of individuals positive (%)	Total no. of pools tested	Total no. of pools positive (%)
Culture +/− microscopy	1286	35 (2.7)	2919	688 (24)
Combination culture and serology	37,603	244 (0.6)^a^	1928	389 (20)
Combination culture and molecular	–	–	229	22 (9.6)
Combination serological and molecular	8019	90 (1.1)	347	195 (56)
Serological	45,439	2260 (5)	1170	123 (11)
Molecular	30,767	588 (1.9)	1343	411 (31)
Unknown	?	9	80	24 (30)

^a^One study reported 75% *O. tsutsugamushi* infection rate in pools made up of 20,700 *L. deliense*, but the number of pools tested and number positive were not reported [257]

*Key*: +/−, with or without; ?, unknown

*Note*: Percentages shown in parentheses were pooled by giving equal weight to all studies

#### Orientia tsutsugamushi testing of key vector species by laboratory test category

A summary of *O. tsutsugamushi* testing of individual and pooled vectors collected from vertebrate hosts using different categories of laboratory test are shown in Table 2 for the three most frequently reported species of chigger. The median positive per site varies significantly dependent on the number of studies and size of studies.

**Table 2:** Summary of total and median tested and *O. tsutsugamushi* positive for the three most frequently reported *Leptotrombidium* chigger species, subdivided into laboratory test categories

Laboratory method	Vector species name	Total no. tested	Median no. tested	Range	Total no. positive	Median no. positive/study site	Median positive (%)
Combination culture and molecular							
	*L. deliense*	0^a^	0	0	0	0	0
Pools	*L. scutellare*	10^b^	10	10	8	4	40.0
	*L. pallidum*	?^b^	?	?	3	3	–
Combination culture and serology							
	*L. deliense*	?^b^	?	?	3	3	–
Pools	*L. deliense*	?^b^	?	?	2	2	–
	*L. scutellare*	10	10	10	4	4	40.0
Pools	*L. scutellare*	15	4	1–10	5	2.5	63.0
	*L. pallidum*	1811^b^	66	23–734	75	12	18.0
Pools	*L. pallidum*	115^b^	15	1–52	68	5	33.0
Combination culture +/− microscopy							
	*L. deliense*	7^b^	4	1–6	1	1	25.0
Pools	*L. deliense*	398^b^	7	1–131	193	3	43.0
Pools	*L. scutellare*	183^b^	24	11–148	5	1	4.2
	*L. pallidum*	17^b^	8.5	1–16	8	4	47.0
Pools	*L. pallidum*	36^b^	3	1–30	13	1	33.0
Serology							
	*L. deliense*	1874	314	285–1275	51	4	13.0
Pools	*L. deliense*	665	333	5–660	18	9	27.0
	*L. scutellare*	1242	131	1–1110	6	3	2.3
	*L. pallidum*	1900	42	12–1263	202	10	24.0
Pools	*L. pallidum*	?^b^	?	7–?	73	4	–
Molecular							
	*L. deliense*	44	22	15–29	3	1.5	6.8
Pools	*L. deliense*	515	6	1–315	124	3	50.0
	*L. scutellare*	3053	54	11–1907	57	5	9.3
Pools	*L. scutellare*	127^b^	4	1–105	35	1	25.0
	*L. pallidum*	1357	38	1–474	57	7	18.0
Pools	*L. pallidum*	4	2	1–3	0	0	0
Combined molecular and serology							
Pools	*L. deliense*	42	42	42	25	25	60.0
	*L. scutellare*	2050	119	6–579	22	2	1.7
	*L. pallidum*	2735	80	1–1420	31	3	3.8

^a^One study reported 75% *O. tsutsugamushi* infection rate in pools made up of 20,700 *L. deliense*, but the number of pools tested and number positive were not reported [258]

^b^Includes studies where number of individuals/pools tested was not given (i.e. no denominator)

*Key*: +/−, with or without; ? unknown

#### Orientia tsutsugamushi testing of free-living vectors

A separate analysis of free-living trombiculid mites [larvae (chiggers), nymphs and adults] was carried out. In view of our current understanding of the life-cycle of trombiculid mites, *O. tsutsugamushi*-infected free-living larvae should be considered potential vectors, although not necessarily to humans. In total 40,995 individual and 266 pools of trombiculid mites were tested. Infection rates for individuals were: 413/18,945 (2.2%) with culture alone, 380/15,852 (2.4%) with serological tests, 304/6125 (5%) using combined culture and serology and 7/73 (9.6%) with molecular tests.

Thirty-one species of trombiculid mite were tested for *O. tsutsugamushi* and at least 23 species were positive (Table 3). All species were of the genus *Leptotrombidium* apart from *Eutrombicula wichmanni*, *Odontacarus* sp. and *Microtrombicula chamlongi*, all of which were reported positive from a single study in Thailand using immunofluorescence [37], *Neotrombicula japonica* from 1 study in Japan [38] and *Helenicula miyagawai* from Mt. Gwanak, outside Seoul, South Korea [39]. Of *Leptotrombidium* species tested in the greatest numbers, *L. pallidum*, *L. deliense*, *L. scutellare* and *L. fletcheri* were the most frequently positive.

**Table 3:** Summary of free-living trombiculid mites (larvae, nymphs and adults) tested by different laboratory categories. All species testing positive for *O. tsutsugamushi* at least once are shown with total and median numbers tested and testing positive

Laboratory method	Vector species^a^	Total no. tested	Median no. tested	Range	Total no. positive	Median no. positive/study site	Medianpositive (%)
Combination culture and serology							
	*L. intermedium*	3237	3237	3237	3	3	0.1
	*L. pallidum*	1879	940	53–1826	288	144	15.3
	*L. deliense*	570	570	570	10	10	1.8
	*L. palpale*	177	177	177	0	0	0
	*L. scutellare*	113	56	19–94	2	1	1.8
	*L. vivericola*	80	80	80	1	1	1.3
Pools	*L. intermedium*	2	2	2	1	1	50.0
Combination culture +/− microscopy							
	*L. deliense*	7060	3530	1180–5880	413	413	11.7
	*G. cassiope*	6120	6120	6120–6120	0	0	0
	*A. indica*	4930	4930	4930	0	0	0
	*L. scutellare*	65	65	65	0	0	0
Pools	*L. deliense*	2	2	2	1	1	50.0
Pools	*L. pavlovskyi*	?	?	?	1	1	–
Serology							
	*L. scutellare*	8444	591	235–2443	41	1	0.2
	*L. deliense*	3030	289	41–949	132	16	5.5
	*L. intermedium*	1208	1208	1208	2	2	0.2
	*L. pallidum*	743	743	743	134	134	18.0
	*L. keukenshrijveri*	646	646	646	15	15	2.3
	*L. fletcheri*	404	202	13–391	14	7	3.5
	*L. vivericola*	358	358	358	15	15	4.2
	*L. arvinum*	181	90	58–123	9	4.5	5.0
	*Leptotrombidium* sp.	127	127	127	9	9	7.1
	*Odontacarus* sp.	81	81	81	3	3	3.7
	*L. bodense*	77	21	9–47	2	2	9.5
	*L. peniculatum*	67	67	67	1	1	1.5
	*E. wichmanni*	23	23	23	2	2	8.7
	*M. chamlongi*	5	5	5	1	1	20.0
Molecular							
	*L. scutellare*	27	27	27	1	1	3.7
Pools	*L. scutellare*	242	121	8–234	7	4	3.3
Pools	*L. fuji*	14	14	14	1	1	7.0
Pools	*N. japonica*	1	1	1	1	1	100

^a^*Leptotombidium akamushi*, *L. pavlovskyi* and *Helenicula miyagawai* were also reported as testing positive, but without a denominator

*Abbreviations*: *L*, *Leptotrombidium*; *G*, *Guntheria*; *A*, *Ascoschoengastia*; *E*, *Eutrombicula*; *M*, *Microtrombicula*; *N*, *Neotrombicula*

*Key*: +/−, with or without; ? unknown

#### Distribution of key vector species

**Fig. 5:**
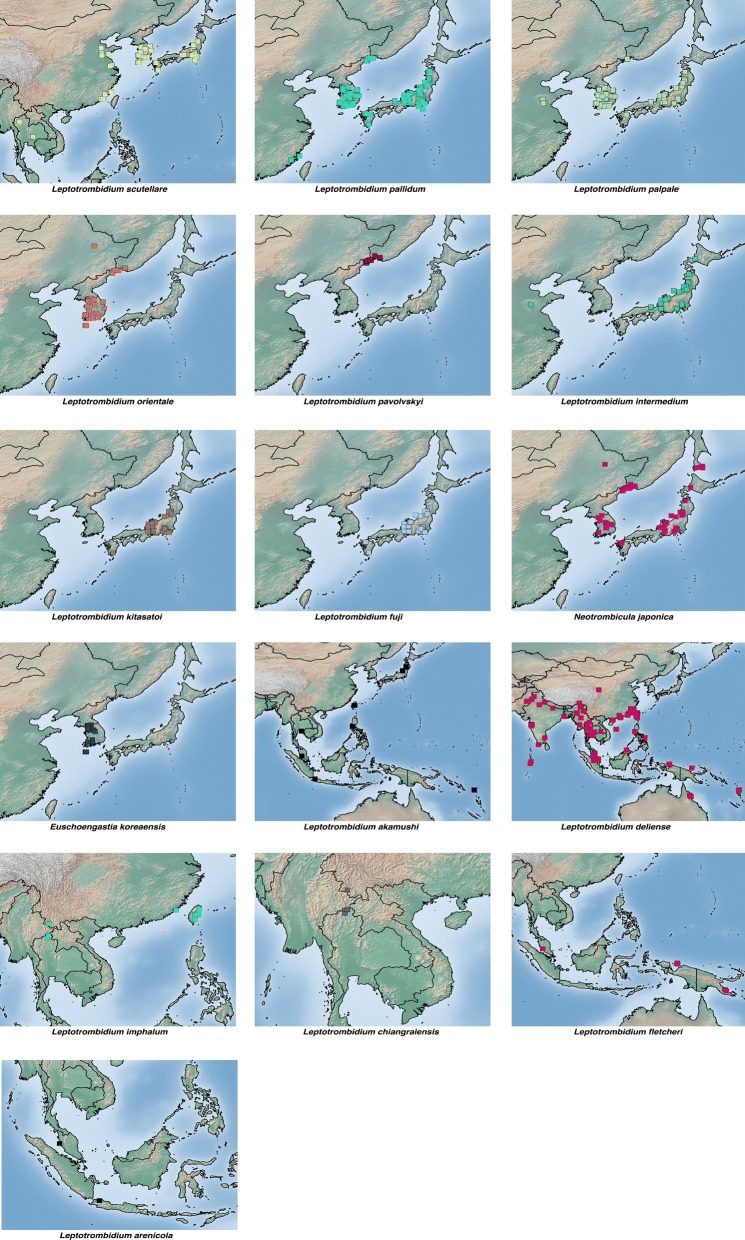
Distribution maps of the 16 most frequently reported *O. tsutsugamushi*-positive chigger species from all studies included in this review

There is very little published information summarising the distribution of chigger species considered important human vectors of scrub typhus. Kim et al. [40] recently reported the distribution of 9 representative *Leptotrombidium* species. The locations of the 16 most frequently positive trombiculid species from articles included in this review are shown in Fig. 5.

#### Other positive trombiculid species

**Fig. 6:**
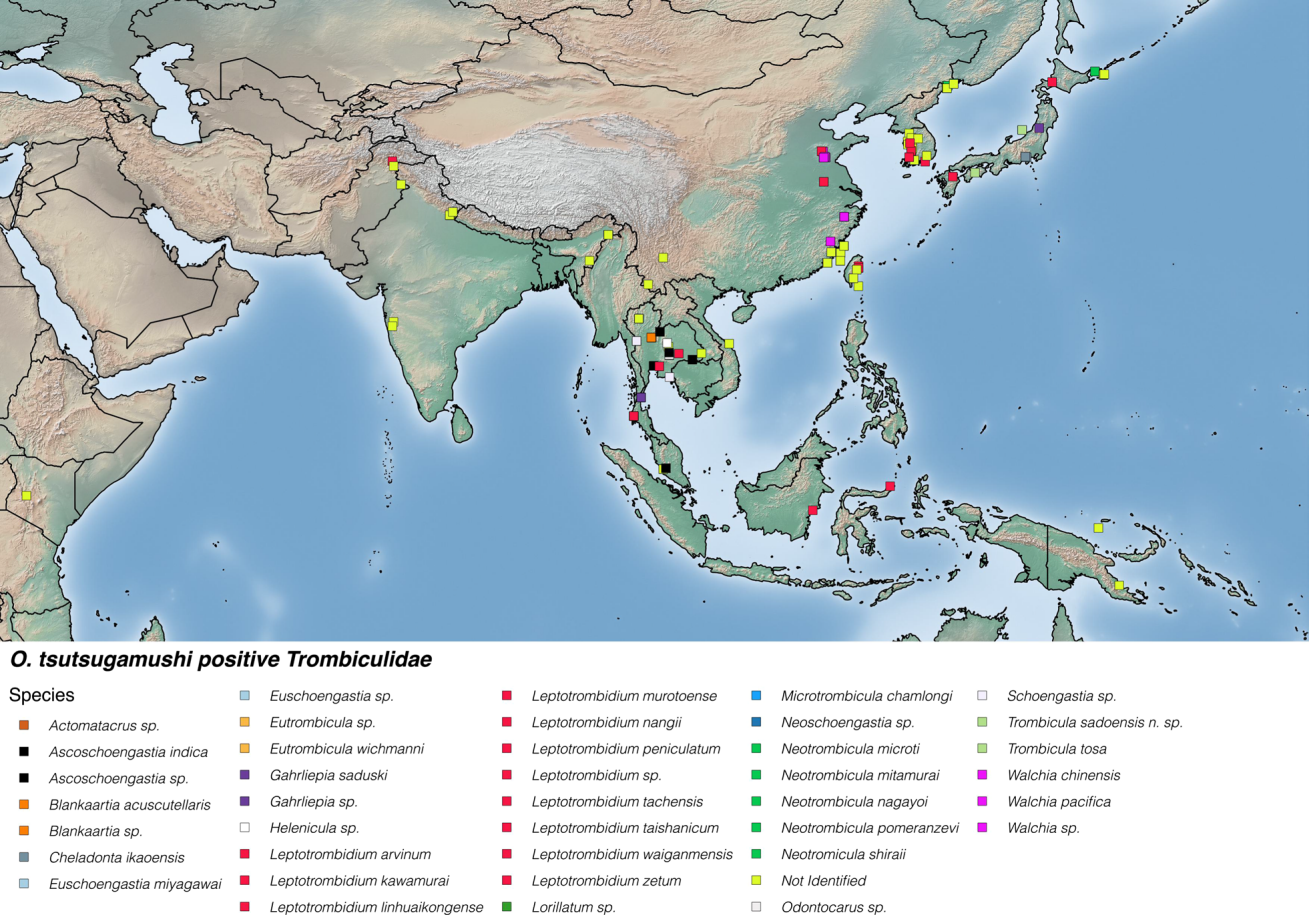
Location of all other trombiculid mite species not listed in Fig. 5 testing positive for *O. tsutsugamushi* including those identified to genus level only and unidentified chiggers

*Orientia* sp. positive tests have been reported from a further 17 genera of trombiculid mites, made up of at least 32 species (Fig. 6). Figure 6 includes chiggers reported to genus only (including *Leptotrombidium*) and unidentified *O. tsutsugamushi* positive chiggers to provide a complete map of positives. It is likely that some of these will be among the 16 species shown above. These are distributed across the Asia–Pacific region, with the exception of the recent report of an organism close to *O. chuto* in either *Microtrombicula* or *Eutrombicula* species of chiggers in Kenya [22]. The robustness of these data is variable, with many different laboratory tests, of variable specificity, used to identify the presence of *Orientia* in vectors. Furthermore, for a number of species, trombiculid mites were pooled and the possibility of mixed-species pools remains. A full analysis of *O. tsutsugamushi*-positive species is given in Additional file 1: Table S5.

### Hosts

#### Orientia tsutsugamushi testing of major host animal groups

A total of 234 species of “host” vertebrates (excluding humans) were tested for *O. tsutsugamushi*, with 122 species testing positive (Additional file 1: Table S6). In Table 4 all different forms of laboratory tests are combined. A large number of hosts were reported here as either: ‘species not identified’ or ‘multiple species listed’ (as for vectors above).

**Table 4:** Summary of number of tested hosts and *O. tsutsugamushi* positives of all species, subdivided by laboratory test category

Laboratory test category	Total no. of individuals tested	Total no. positive	Percent positive
Culture +/− microscopy	16,486	2943	18
Combination culture and serology	14,195	2761	19
Combination culture and molecular	389	91	23
Microscopy alone	250	6	2
Combination serological and molecular	3443	1387	40
Serological	36,089	10,874	30
Molecular	12,198	1170	10
Unknown	169	10	6

*Key*: +/−, with or without

Serological tests were performed most frequently, with 36,089/83,219 (43%) hosts being tested using these methods. These were also most frequently *O. tsutsugamushi* positive at 10,868/35,960 (30%). Culture with or without microscopy and culture with serological confirmation were next most frequent with 16,486/83,219 (20%) and 14,195 (17%) tests performed, respectively, and similar rates of positivity at 2943/16,486 (18%) and 2761/14,195 (19%), respectively. Although rarely performed, microscopy alone expectedly had the lowest rates of positivity at 6/250 (2%). Molecular methods were used to test 12,198 (15%) of hosts with 1170/12,198 (10%) positive (Table 4).

To assist with summarising the results, the 234 species tested were classified into 21 groups (Table 5). The testing of non-human hosts for *O. tsutsugamushi* has been performed primarily on small mammals, long considered the major hosts for vector trombiculid mites. The Muridae (rats and mice) included the major identified species tested at 52,670/62,726 (84%). The Cricetidae (voles, hamsters, etc.), Soricidae (shrews) and Sciuridae (squirrels) constituted just 4%, 2% and 2.7%, respectively. Birds (Aves) constituted 0.5% (Table 5).

**Table 5:** Summary of percentage of hosts testing *O. tsutsugamushi* positive, subdivided into taxonomic groups

Group	Major species tested	Total no. of individuals tested	Total no. positive (all test types)	Percent positive
Artiodactyla^a^	Cow, sheep, goat, pig	1568	54	3.4
Aves	Chicken, *Passer domesticus*, *Motacilla cinerea*	293	16	5.5
Canidae^b^	Dog, *Cerdocyon thous*	1826	325	17.8
Chiroptera^c^	*Rhinolophus ferrumequinum*, *Eptisicus serotinus*	797	99	12.4
Cricetidae	*Cricetulus triton*, *Microtus fortis*, *Myodes glareolus*	2516	308	12.2
Echimyidae^d^	*Thrichomys fosteri*	85	0	0
Erinaceidae	*Echinosorex gymnura*	8	0	0
Felidae^d^	*Leopardus pardialis*	7	0	0
Herpestidae	*Herpestes javanicus*	1	0	0
Lagomorpha	*Ochotona roylei*	6	0	0
Marsupialia	*Isoodon macrourus*, *Thylamys macrurus*	285	37	14.5
Muridae	*Apodemus agrarius*, *Rattus rattus*, *Rattus norvegicus*, *Rattus tiomanicus*, *Bandicota indica*	52,670	13,419	25.5
Mustelidae	*Melogale personata*	7	1	14.2
Reptilia	Lizards, *Physignathus lesuerii*	61	0	0
Sciuridae	*Callosciurus notatus*, *Tamias sibiricus*	1692	105	6.2
Simiformes	*Macaca fascicularis*	27	12	44.0
Soricidae	*Suncus murinus*, *Crocidura lasiura*	1247	165	13.2
Talpidae^e^	*Urotrichus talpoides*	13	3	23.0
Tupaiidae	*Tupaia glis*	333	49	14.7
Viverridae^f^	*Paradoxurus hermaphrodites*	6	0	0
Multiple or unidentified species	–	20,056	4649	23.2

^a^Only performed in China, Taiwan and Russia

^b^Only dogs positive

^c^Single study from South Korea using serology

^d^Only tested in Brazil

^e^Only tested in Japan

^f^Only tested in Vietnam

Of the major vertebrate groups tested, combining all test types, the Muridae had the highest proportion of positive tests at 13,419/52,670 (25.5%). Of the other major groups of small mammal, the Cricetidae and Soricidae had similar rates of positivity at 12.2 and 13.2%, respectively. Six percent of the mainly arboreal Sciuridae were positive. The Canidae tested 18% positive, with the majority [307/319 (96%)] tested using serological methods. Among the Artiodactyla, cows, goats and pigs were tested by serological methods only, testing 3.6% positive overall. The group listed as “multiple or unidentified species” had a similar positivity rate to the Muridae, most likely because the species composition was similar to that presented overall, with most being Muridae.

#### Orientia tsutsugamushi testing of key host species by laboratory test category

Additional file 1: Table S7 presents data for the 5 most frequently tested species subdivided by laboratory test category. The major species tested and positivity rates are broadly consistent with the overall data presented above. Muridae account for most of the species listed. A large number of dogs were tested serologically in several studies with a median positive rate per site of 5.5%. Two Chinese studies from Bole region of Xinjiang, China, account for the surprisingly large number of sheep testing positive by PCR [41, 42]. Whole blood was collected and tested by 56 kDa PCR.

#### Orientia tsutsugamushi testing of other host species

*Mus* spp. have long been considered as unimportant host species for *O. tsutsugamushi*-carrying chigger species [9]. However, in culture-based studies a median of 16% positive (median positive per study site/median tested at all sites) was seen for wild *M. caroli* and (13.5/34.5) 40% for *M. musculus*.

Many species of the Cricetidae have been tested. Using combination culture and serology, a median of 28% (7.5/27) *Microtus montebelli* and 5% (0.5/10) *M. fortis* were positive. By serology, 17% (1/6) of *Cricetulus triton* and using molecular techniques 3% (1/33) of *Cricetulus migratorius* tested positive.

*Suncus murinus* was the most frequently tested of the Soricidae and a median of 6% (1/15.5) was positive among all studies. Of the Sciuridae, *Callosciurus notatus* was positive with a median of 2% (5/243.5) serologically and 13% (1/8) using combined culture and serology. Chiroptera were tested only by serological methods and 12% (38/308) of *Eptesicus serotinus* and 11% (7/66) *Rhinolophus ferrumequinum* were positive.

For birds, using PCR, 17% (2/12) of *Motacilla cinerea* (grey wagtail) and 10% (1.5/15) of *Passer domesticus* (house sparrow) were positive in China (see below).

### Ecological relationships of vectors and hosts

Bipartite network analysis is becoming increasingly used to understand host-parasite interactions [43]. These networks can help reveal the importance of certain species in the transmission ecology of a disease [44]. Bipartite network figures depicting the relationship between host animal species and vectors were constructed for selected country groups. A quantitative depiction was made for all host/vector species interactions (shown by line thickness). A sub-analysis is also shown for interactions where either a vector or host tested positive for *Orientia*.

**Fig. 7:**
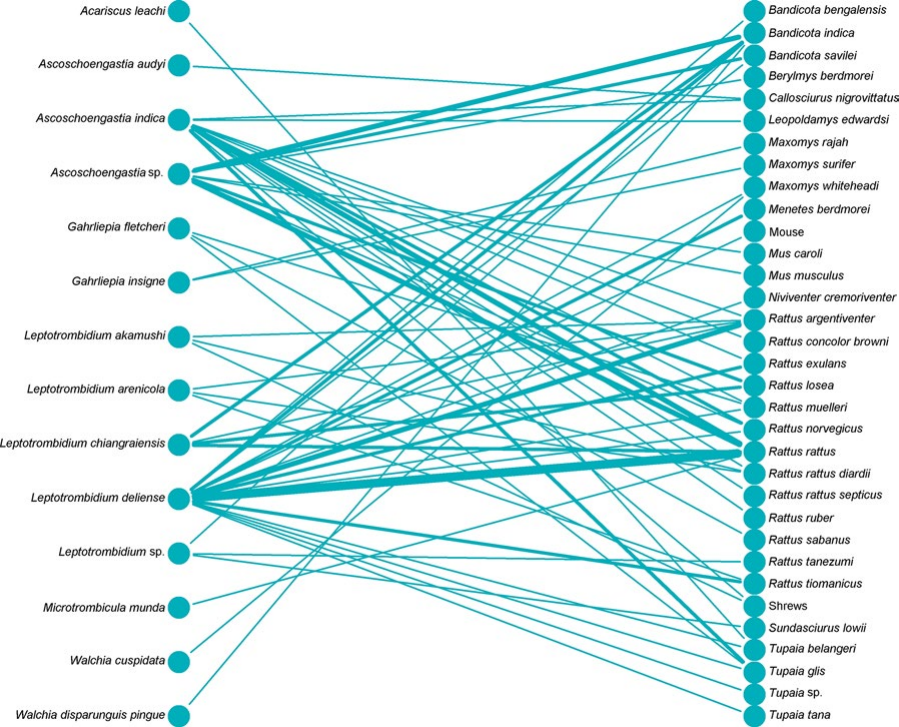
Network analysis of small mammal and chigger species for studies from Southeast Asian countries (Thailand, Vietnam, Malaysia, Indonesia and Myanmar)

**Fig. 8:**
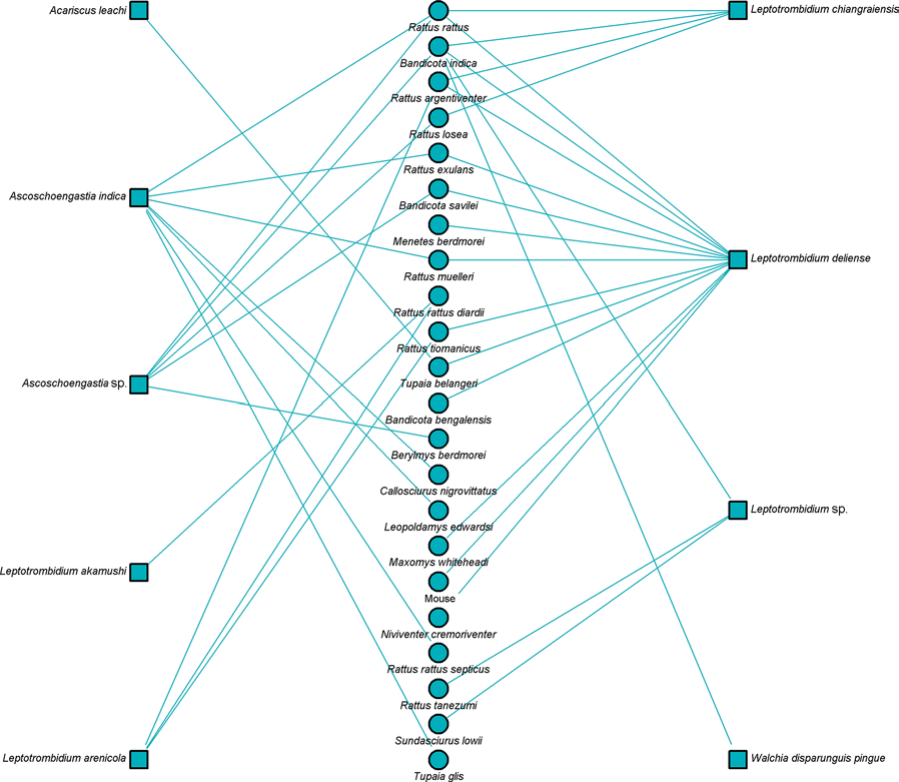
Network analysis of small mammal and chigger species testing positive for *O. tsutsugamushi* by any laboratory test for studies from Southeast Asian countries

**Fig. 9:**
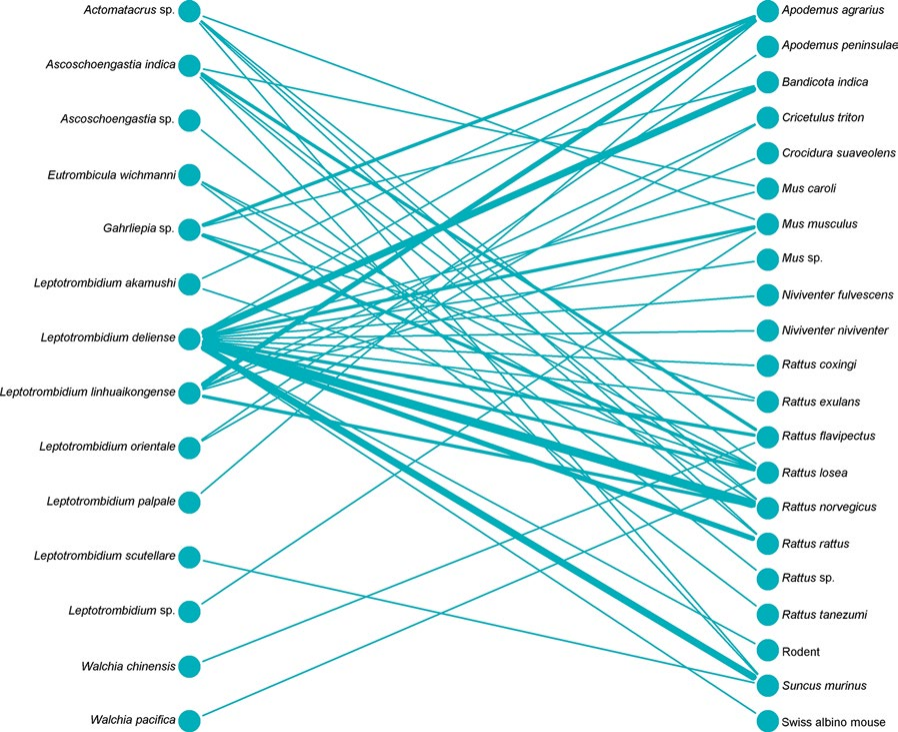
Network analysis of small mammal and chigger species for studies from the People’s Republic of China and Republic of China (Taiwan). The Swiss albino mouse is included where it was used as a bait animal to collect chiggers

**Fig. 10:**
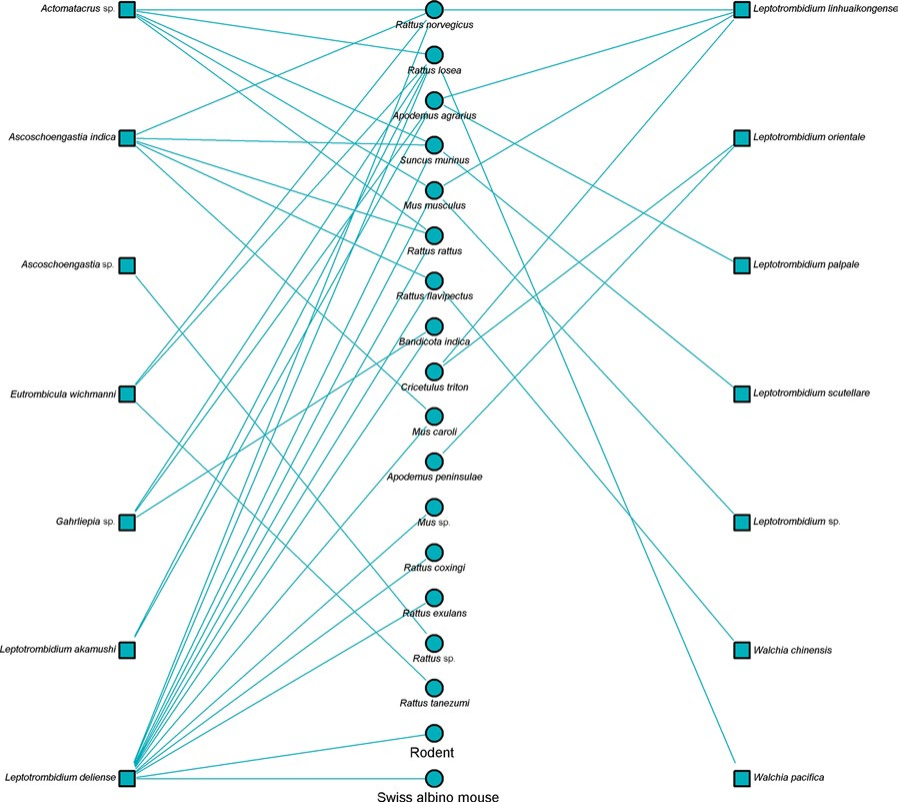
Network analysis of small mammal and chigger species testing positive for *O. tsutsugamushi* by any laboratory test for studies from the People’s Republic of China and Republic of China (Taiwan)

**Fig. 11:**
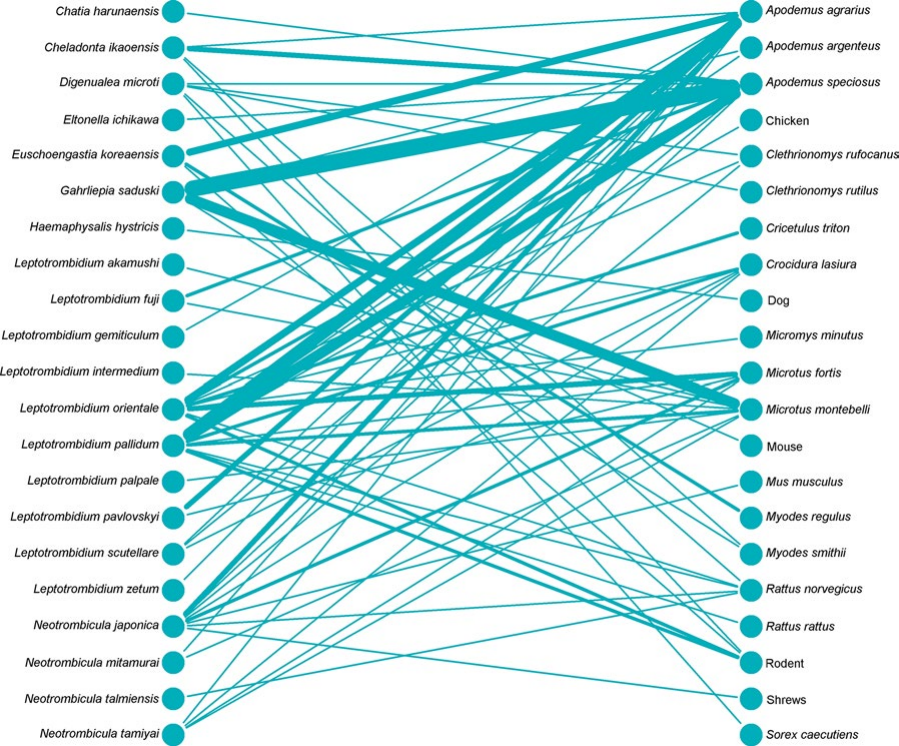
Network analysis of small mammal and chigger species for studies from Japan, South Korea and Russia

**Fig. 12:**
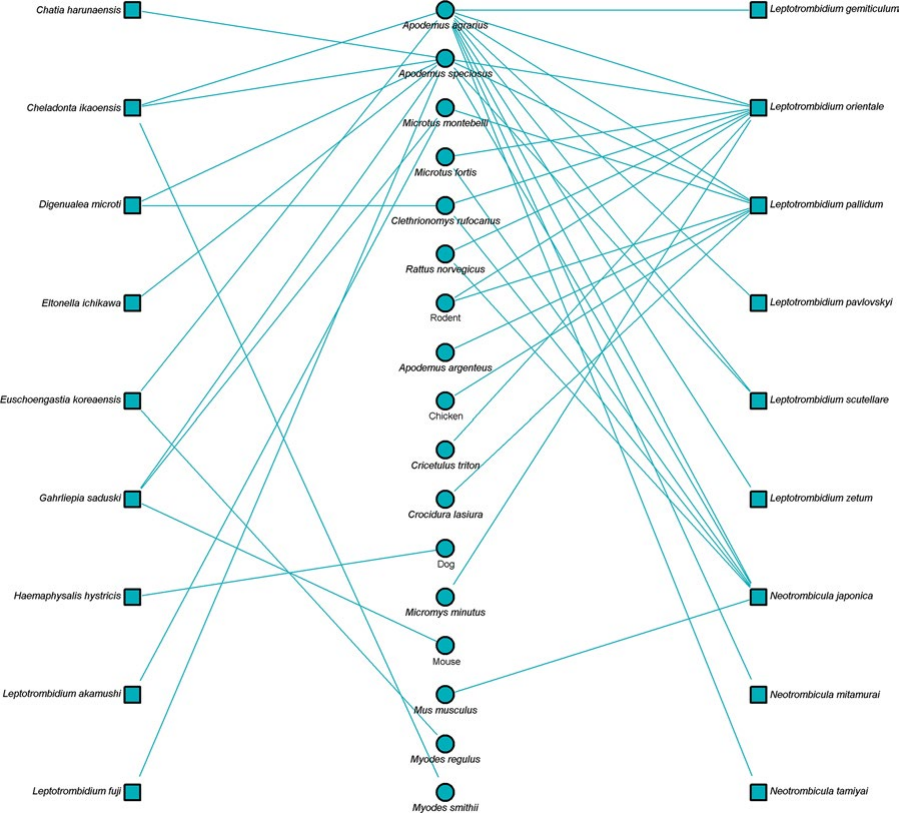
Network analysis of small mammal and chigger species testing positive for *O. tsutsugamushi* by any laboratory test for studies from Japan, South Korea and Russia

Networks for Southeast Asia clearly show *L. deliense* and *Ascoschoengastia indica* as key chigger species whether testing *O. tsustugamushi* positive or not (Figs. 7, 8). Among those testing positive, the pattern of host species was different with little overlap. In the China/Taiwan analysis, *L. deliense* is again the key species and associated with a wide host range (Figs. 9, 10). Networks for Japan, Korea and far-eastern Russia reveal a different pattern of host-vector interactions. Overall *L. pallidum* and *G. saduski* interacting with *Apodemus* spp. and *Microtus* spp. hosts were by far the most frequent (Figs. 11, 12). However, among *O. tsutsugamushi-*positive species, *L. pallidum*, *L. orientale* and *Neotrombicula japonica* appear to be key species.

### Non-chigger species

**Fig. 13:**
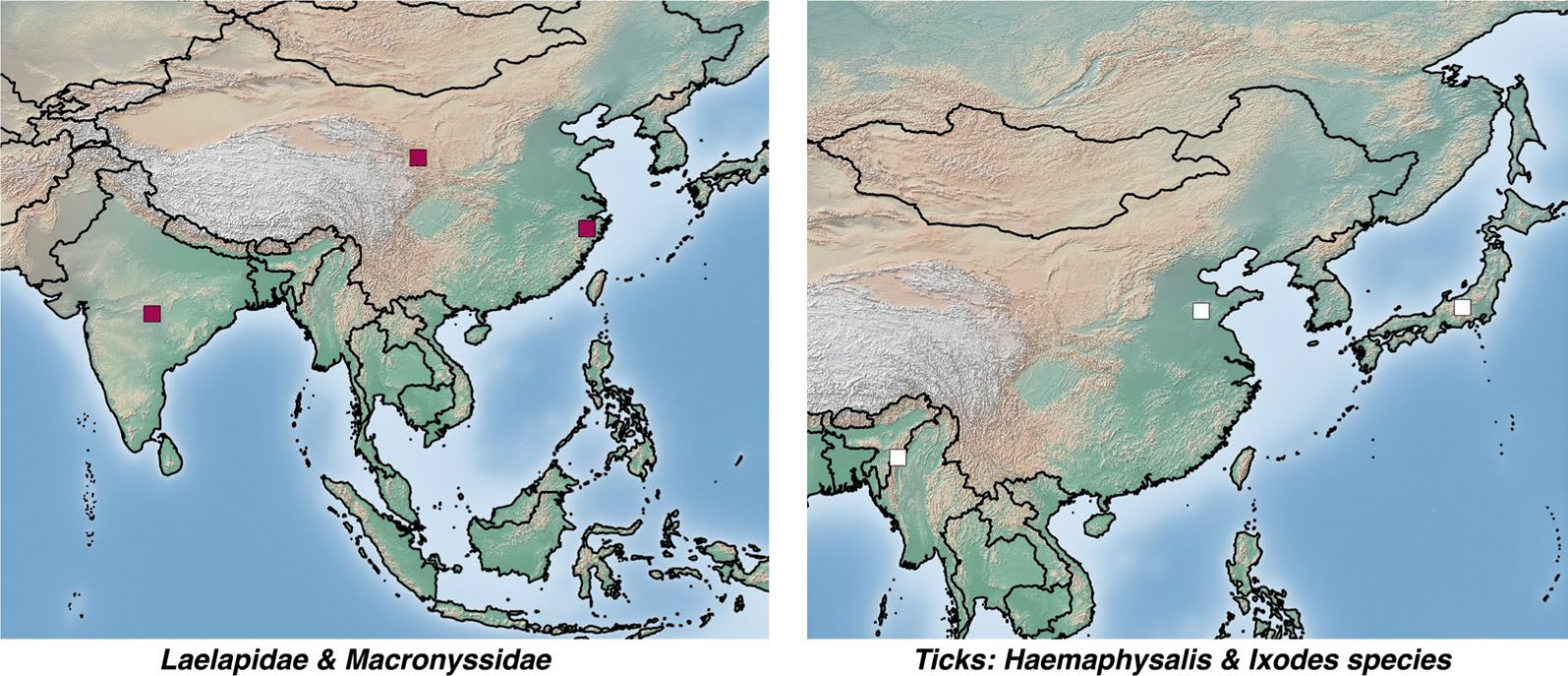
Location of *O. tsutsugamushi*-positive non-trombiculid mite species

Few non-trombiculid mites have been reported as testing positive for *O. tsutsugamushi* (Additional file 1: Table S5). In an unspecified site in Japan, several *Haemaphysalis* sp. ticks removed from scrub typhus-infected dogs tested positive by PCR [45]. A section from Audy’s War Office report also recorded *O. tsutsugamushi* in the same genus of ticks using xenodiagnosis [31]. Two of 12 pools of *Ixodes* sp. ticks removed from rodents tested positive for *O. tsutsugamushi* by PCR in Shandong Province, China [46]. *Ornithonyssus bacoti* (Macronyssidae) removed from rodents in Nagpur, India, tested positive by PCR (1 of 5 pools) [47] and in the review of rickettsial disease in China by Fan et al. [26], *Echinolaelaps echidninus* and *Laelaps turkestanicus* also tested positive, but further details are not provided (Fig. 13).

### Chigger indices, percentage host infestation and Orientia positivity

Only 43/276 (16%) studies provided data on the chigger index (mean number of chiggers per host species) of collected animals. Even fewer studies, just 34 (13%), reported the percentage infestation rates of collected animals (percentage of host species with 1 or more chiggers found attached). Chigger index was reported for 47 host species and percentage infestation for 52 species. Consistent with overall data on hosts tested, the major genera and chigger indices reported were: *Apodemus* spp. (66); *Bandicota* spp. (54); *Mus* spp. (4); *Rattus* spp. (50); and *Suncus murinus* (29) (Additional file 1: Table S8). The median percentage infestation rates were: *Apodemus* spp. (38%); *Bandicota* spp. (95%); *Mus* spp. (27%); *Rattus* spp. (59%); and *Suncus murinus* (59%) (Additional file 1: Table S9).

**Fig. 14:**
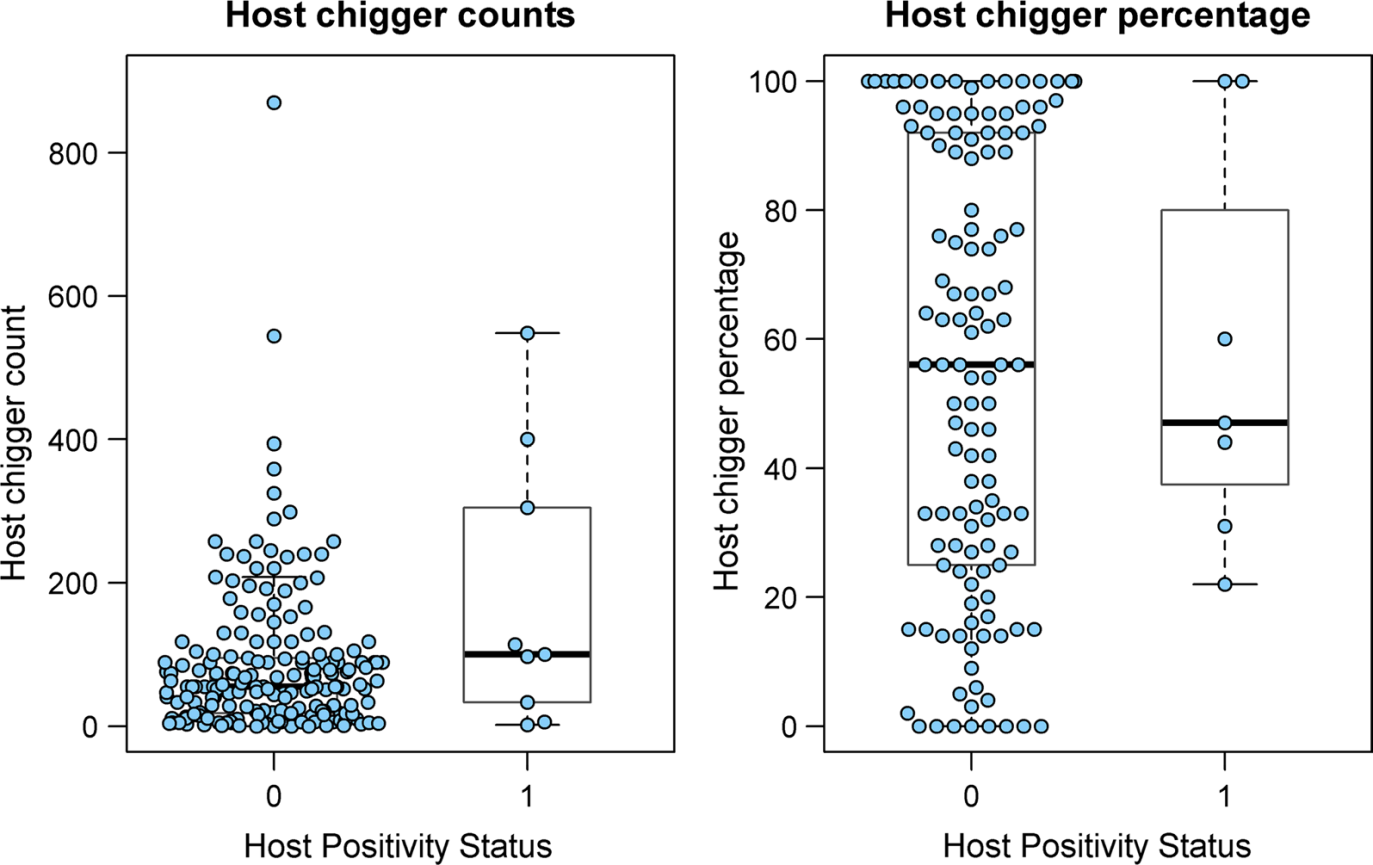
Box plots showing small mammal host chigger index (count) and percentage for *O. tsutsugamushi* positive and negative test results

Chigger index and small mammal infestation rates were compared to *O. tsutsugamushi* positive and negative test results across all species and locations using the Mann–Whitney U-test. There was a significant association of chigger index with positive detection of the pathogen (*P* < 0.005) (Fig. 14). This relationship was not seen with percentage infestation. Only a small number of *O. tsutsugamushi* positive samples were reported for which either the host chigger index or percentage infestation rate was also reported.

### Summary of ecological data

A total of 793 study sites were recorded from 276 included articles. For 610/793 (77%) study sites, no descriptive information on the habitat or other local ecological features was given. At 91 (12%) study sites, only the very briefest and most basic habitat classification was given. Frequently used terms included: scrub, fallow, fields, agricultural land, mountainous, forest and forest edge, rice fields, grass, parks, riverside, orchards and plantations, settlements, urban and rural. More detailed description was provided for 57 (7%) study sites. In these cases, the above terms were usually used with additional, non-scientific habitat description. Common examples included: crop names (banana, tea, sugarcane and sweet potato); forest types (coniferous, deciduous, broadleaf, evergreen and bamboo); and general plant types (*Miscanthus*, lalang, palm, *Pandanus* and *Lantana*).

A small minority of just 31 (4%) study sites provided detailed scientific habitat and or ecological description, including soil type. To be classified as such, at least one plant must have been identified to the species level. Of the 31 sites, 20 were sites where *O. tsutsugamushi* was identified in vectors or non-human hosts. Ten sites were in Japan where commonly reported plants included *Phragmites communis*, *Quercus serrata*, *Cryptomeria japonica*, *Phyllostachys pubescens*, *Artemisia* sp. and others. Of 4 sites in Malaysia, *Imperata cylindrical*, *Paspalum* and *Melastoma* were reported. Additional reports from 2 sites in Tajikistan (*Populus pruinosa*, *Tamarix* sp. and *Salix* sp.), 2 sites in Taiwan (*Bidens pilosa*, *Miscanthus* sp. and *Leucaena* sp.), and single sites from Russia and the Philippines were found. No clearly dominant plant species were reported among the sites, even in the same country. Soil type (loam, red clay, humus, etc.) was recorded at only 5 sites. No analysis of soil was performed in any of the included studies.

A very small number of studies reported rainfall either as an annual figure or total precipitation during the study period. Of the 13 studies, 10 were in China, 2 in India and 1 in Russia. Twelve studies reported average, minimum and maximum temperatures at the study sites. The average temperatures reported ranged from 12 to 26 °C and maximum of 42 °C and minimum of − 2 °C.

### General ecology themes

#### Trombiculid mite life-cycle

**Fig. 15:**
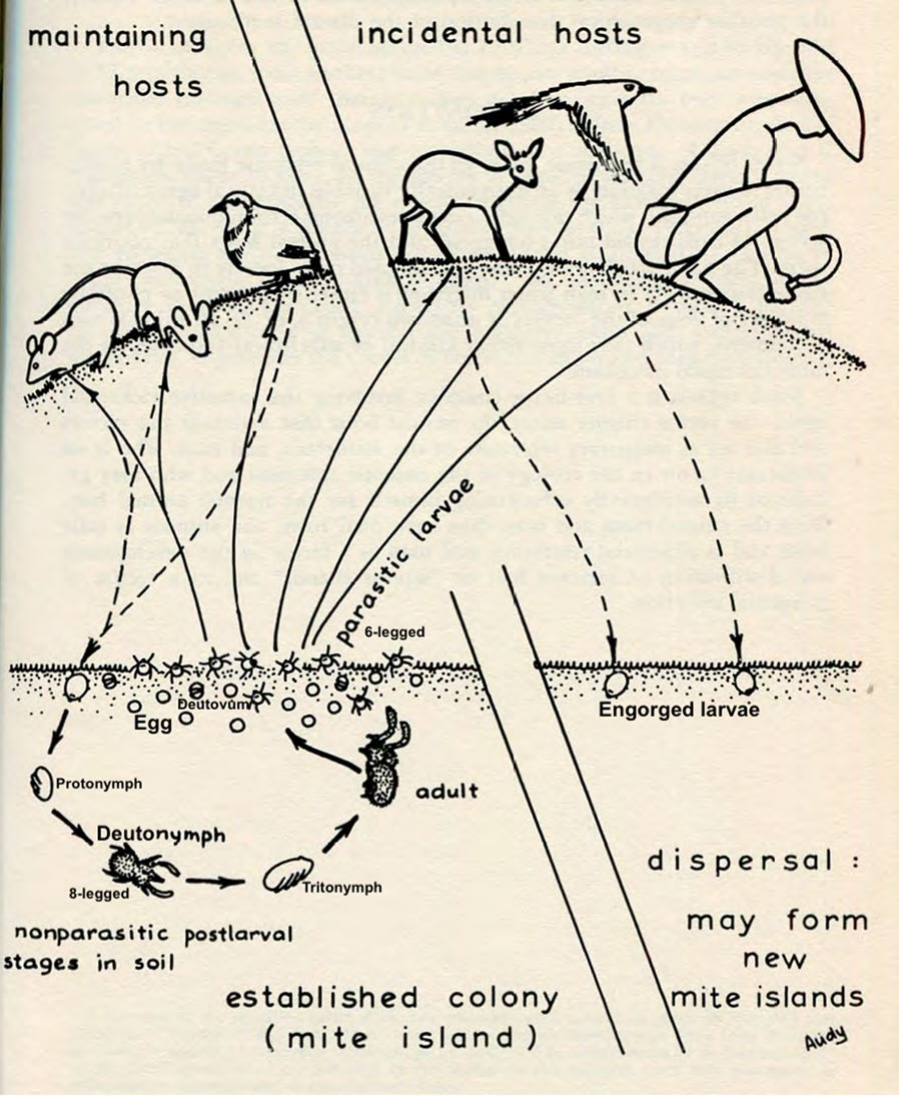
Chigger and scrub typhus life-cycle (adapted from Audy [55])

Many aspects of the life-cycle of chiggers are well understood from laboratory colony studies, although how this may vary in nature is not. Neal & Barnett [48] provided a detailed account of the life-cycle of *Trombicula akamushi* in laboratory conditions. Males produce stalked spermatophores that are deposited in the environment and taken up by females to fertilise their eggs. Egg deposition begins between 6 and 21 days post-insemination. Egg laying continues for as much as 253 days in one case, with mean daily egg production ranging from 2.4 to 21.7 and a maximum of 41 recorded. After 7–11 days the ovum ruptures to produce a quiescent deutovum. A further 5–7 days later the 6-legged larva emerges. These larvae remain within a few centimetres of their birthplace and after 2 days may start to display host-seeking behaviour by forming clusters on leaves, grasses and twigs above the soil surface. Larvae can survive for many months awaiting the opportunity to feed. The larvae feed on digested tissue fluid from a vertebrate host, becoming engorged and increasing in size by several fold. Larvae will feed from anything between 2 to 12 days or longer, depending on the chigger species. They then detach and return to a suitable habitat on the soil surface. Over about 3 days they develop into another quiescent phase: the nymphophane or protonymph. After a further 7–10 days the 8-legged deutonymph emerges. This, and the adult stage, feed on arthropod eggs (e.g. *Culex* mosquito eggs), or recently deceased or quiescent soft-bodied insects such as Collembola [49, 50]. Two weeks later the nymph develops into the tritonymph (teleiophane), lasting about 2 weeks from which the adult finally emerges [9, 48, 51–56] (Fig. 15). Adults may survive for 15 months or more. Their food preferences in the wild for different species remain unknown. In some tropical species of *Leptotrombidium*, the entire cycle may take just 2 or 3 months, allowing at least 2 generations per year. Only a single generation is possible in more temperate zones [9]. It can frequently be seen that groups of attached chiggers are of similar size, suggesting that opportunities may arise for batches of chiggers, possibly from the same brood, to latch onto a host [9].

This detailed understanding of the trombiculid mite life-cycle is almost entirely derived from studies on mites kept in laboratory colonies, due to the challenges involved in observing mites in their natural habitat. It is thus unknown how these stages may vary in nature.

Comparisons in the life-cycle between *O. tsutsugmaushi* infected and uninfected chigger lines of *L. imphalum* and *L. changraiensis* have suggested developmental stages are delayed in infected lines, but host-feeding time is reduced. A shorter feeding time may confer a survival advantage [57]. From laboratory studies, typical female to male ratios of offspring range from 5:1 to 2:1. However, in infected chiggers, 95–100% are female [29, 57–62]. Several maternally inherited bacterial species including *Rickettsia* are known to manipulate sex ratios towards female offspring [63–66]. The exact reasons and mechanisms by which *O. tsutsugamushi* manipulates sex ratios in mites remain unknown. The occurrence of infected males is rare but has been reported in *L. pallidum* [67], *L. imphalum* [68], *L. arenicola* [62] and *L. fletcheri* [62]. However, *O. tsutsugamushi* is not found in spermatophores [69]. Parthenogenesis has been demonstrated in *L. arenicola*, but to what extent this occurs in nature and in other species is not known [70].

#### Transovarial and transstadial transmission

Transmission of *O. tsutsugamushi via* the ovum is known as transovarial transmission and through the various stages of the life-cycle as transstadial transmission. Nagayo & Kawamura first recognized this in the 1920s [71, 72]. In adults, the ovaries appear to most frequently harbour *O. tsutsugamushi* compared with other organs [73]. In larvae, a higher incidence of *O. tsutsugamushi* in the salivary organs was seen in unengorged larvae than in engorged [74, 75].

Transovarial transmission of *Orientia* has shown to be effective in chiggers kept in the laboratory. Rapmund et al. [29] reported close to 100% efficiency over 4 generations in *L. akamushi*. In *L. deliense* and *L. arenicola*, transmission to the F2 generation was approximately 95% and 92.6%, respectively. However, a marked decline in transmission was seen after 17 generations with fewer eggs produced [76, 77]. Similarly, rates of 93–100% were reported from Malaysia and Thailand in *L. chiangraiensis*, *L. fletcheri* and *L. arenicola* [58, 78]. Transstadial transmission must clearly be present if transovarial transmission rates are high; however, evidence of *O. tsutsugamushi* in different life stages has been difficult to demonstrate. Several studies showed lower rates of *O. tsutsugamushi* isolation in stages other than engorged larvae, particularly in eggs, deutova and adults [29, 59, 73, 79]. This was also noted when free-living adults and nymphs were collected from a hyperendemic area in Malaya and all tested negative [80]. These findings led investigators to postulate whether *O. tsutsugamushi* are in some way reactivated during the feeding stage and become occult in other stages [9, 73].

Whether small mammals or other hosts can act as a reservoir of *O. tsutsugamushi* infection is of crucial importance in understanding the ecology and population genetics of this pathogen. To date there has been relatively little investigation of this, all performed in the laboratory setting. In one study, 3 different species of chigger were allowed to feed on experimentally infected wild-caught rodents. After 10 days, 9.1% of *L. fuji*, 5% of *L. pallidum* and 0% of *L. deliense* were infected and at 20 days all were negative [81]. In a further study, Takahashi et al. [82] were able to demonstrate transstadial transmission but no transovarial transmission. Walker et al. [60] carried out a similar study with *L. deliense* and *L. arenicola*. No *L. arenicola* acquired *O. tsutsugamushi*; however, 7.5% of *L. deliense* did, with evidence of transstadial transmission to the adult stage, but transovarial transmission could not be demonstrated. Toyokawa [83] was able to demonstrate that several typical vector species including *L. akamushi* and *L. scutellare* were able to acquire *O. tsutsugamushi* during feeding. Traub et al. [84] also investigated this question and showed 60–100% of engorged larvae (tested in pools) were *O. tsutsugamushi*-positive 1–4 days after detaching. This figure fell to 10–27% after 5–15 days. A single case (1/43) of transovarial transmission was reported. In 2009, Frances [85] re-examined this subject and again demonstrated that *L. deliense* feeding on an infected host will acquire *O. tsutsugamushi* and transmit it transstadially. Indeed, infection could be demonstrated in all life stages, but no vertical transmission occurred. A single case of transovarial transmission in *Blankaartia acuscutellaris* occurred, despite the fact that the engorged larvae tested negative. This case was hard to explain, but could reflect very low levels of *O. tsutsugamushi* post-feeding. The possibility remains for *O. tsutsugamushi* to be boosted in the chigger population from wild small mammals.

The existing evidence suggests that chiggers probably act as both host and reservoir of the disease. There is much uncertainty about the interaction between *Orientia* and transovarial and transstadial transmission. The lack of evidence of vertical transmission of *O. tsutsugamushi* following acquisition from an infected host in the laboratory, led to speculation that this event may be so rare as to be probably irrelevant in nature. However, evidence is growing that for many vector-borne pathogens, the prevalence in vectors is very low and transmission is inefficient. For example, tick-borne encephalitis virus prevalence was 0.5–2% in *Ixodes ricinus* in endemic areas [86] and 1.7% of the same species of tick were infected with *Anaplasma phagocytophilum* in Switzerland [87]. Even if transmission is infrequent in nature, this may be sufficient to maintain and spread the pathogen in the population.

#### Chigger behaviour

Larval chiggers are present in a huge range of habitats, depending on species, where they await the opportunity to attach to a suitable host to feed. During this time, chiggers remain very still, probably moving less than 45 cm [88]. Chiggers are primarily stimulated by carbon dioxide, exhaled from an approaching host. They display negative geotaxis and phototaxis, and neither sound, heat, vibration, human sweat nor saliva, nor various other chemicals could induce a clear questing response [89–92].

Chiggers tend to be inactive until the temperature rises above 10 °C and begin to crawl above 12 °C. Chiggers can crawl at approximately 10 cm per minute at 28 °C [93]. They are surprisingly hardy, surviving for 60 days at 1–2 °C and even − 20 °C for a month [93]. Chigger survival submerged in water for 2 weeks (with subsequent normal continuation of the life-cycle) has been recorded, with important implications in its ecology [52, 84].

#### Chigger feeding

Once aboard a host, chiggers may move around for some time, before attaching at a suitable site to feed [9]. The larva attaches by means of its sharp mouth parts (chelicerae) and develops a characteristic feeding tube (stylostome) over several hours. The stylostome is formed by the chigger’s salivary enzymes, which contain homologues of tick cement proteins and probably form the structure of the tube on contact with host tissues [94]. The stylostome may extend for 120 μm or more. Below the distal end of the stylostome a pool of digested epithelioid and lymphoid tissues is created and this is “sucked” up by the chigger [95–97]. There is little evidence that chiggers routinely ingest blood, particularly as the stylostome probably only extends a little beyond the epidermis. Traub et al. [84] and Traub & Wisseman [98], however, reported visualising red blood cells within the stylostome. Presumably, *O. tsutsugamushi* is usually acquired from lymphoid tissue, although no known animal studies have demonstrated this.

It is widely reported in the literature that chiggers feed only once during their life-cycle, attaching from anywhere between 2 days and several weeks depending on the species [52, 53, 99–104]. In temperate climates, some species of chigger appear to overwinter on the host and detach once conditions are suitable for development into the next instar [105]. If this is the case, then axiomatically chiggers must be able to act as both vector and host. Traub et al. [84] reported that when a host was killed once chiggers had begun to engorge, 250/1000 individuals detached and reattached to a new host. Transmission of *O. tsutsugamushi* to the second host could not be demonstrated. They went on to claim that 5% of chiggers feeding on a host will voluntarily detach and after regressing in size, one third reattached to a new host [9]. However, Kohls et al. [106] could not elicit any reattachment of chiggers to a second host. The lack of studies in this area reflects the challenges involved. Traub & Wisseman [98] postulated that reattachment might be a more important phenomenon in nature, with frequent small mammal deaths. Live-trapped animals examined immediately after death frequently have a large proportion of unattached chiggers present on the animal’s body, together with clusters remaining attached (personal observation). To what extent these represent detached chiggers, or chiggers in the process of finding a site for attachment is unknown.

The phenomenon of co-feeding chiggers and the role this plays in exchanging *O. tsutsugamushi* between individual vertebrates is a much-overlooked subject. Chiggers tend to aggregate into tight clusters to feed, very often consisting of the same species and same age. Frances et al. [107] were able to show transmission of *O. tsutsugamushi* to uninfected *L. deliense* (1.6%) and *B. acuscutellaris* (2.5%) co-feeding with infected chiggers. This may not only act as an important method of horizontal transfer of *O. tsutsugamushi* between individuals of the same and other species, but also account for some of the enormous strain diversity and existence of multiple strains in the same individual vertebrates [108, 109]. The mechanisms and processes driving the evolution of strain diversity and the interaction of the pathogen between chiggers and mammal hosts (including humans) is an important area for future research.

#### Criteria for vectorship

To definitively establish vectorship, several criteria should be met. The vector must be naturally infected with the pathogen and it must be able to transmit it to a host. The vector should be prevalent in the place where infection occurs and naturally infected hosts should be confirmed. Finally, evidence should be found of the vector feeding on a host, including humans [9]. Due to the size of chiggers, the latter can be challenging, although has been reported [88, 110].

It is possible that different species of chigger have different propensities for biting certain vertebrates. Traub & Wisseman [9] suggested that intrazootic chigger species may exist that maintain *O. tsutsugamushi* among, for example rodents, but are not predisposed to bite humans. In a human volunteer study, *L. fletcheri*, a well-established vector species in Malaysia, readily attached to humans, whereas *L. arenicola* did not, even when kept on the skin in a capsule for 24 hours [111].

#### Habitats and microhabitats

The Oxford English Dictionary defines scrub as: “[ME, var. of shrub], (a) vegetation consisting mainly of brushwood or stunted forest growth, (b) land covered with this”. Evidence suggests that scrub typhus is present in a far greater range of habitats than that described by the word “scrub”. *Orientia tsutsugamushi* in vectors and hosts has been found on sandy beaches in Malaya [112], in deep jungle [113], in semi-urban or peri-urban environments [39, 114], in localized areas within semi-desert, alpine meadows and subarctic glacial moraine at 3200 m above sea level in West Pakistan [24].

Chiggers appear to be relatively habitat specific, although some key vector species, such as *L. deliense*, seem to be able to colonise a greater range of habitats. *Leptotrombidium deliense* is frequently found in scrub and forest, while *L. fletcheri* may be collected from certain grassy areas and *L. arenicola* from vegetation alongside beaches [115, 116].

At the larger scale, certain general types of habitats seem to favour the presence of scrub typhus. However, it must be remembered that the presence of sufficient numbers of a maintaining host animal is inextricably linked to the presence of the vector and therefore the disease. Neither chiggers nor vertebrates they feed on are uniformly distributed in any environment. During WW2, Audy et al. [31] conducted detailed investigations into the association of both human cases and vector chigger species with different habitats. This is chronicled in the 403-page report of the Scrub Typhus Research Laboratory, South East Asia Command published in 1947 [31]. Three key risk habitats were identified: (i) artificial wasteland as a result of (a) rural abandoned clearings due to shifting cultivation practices, (b) domestic or suburban neglected areas or (c) neglected gardens and plantations; (ii) water meadows including the grassy edges of water bodies and seepages in drier areas; and (iii) hedgerows or fringe habitats, typically where two types of habitat meet such as forest edges (ecotones) [54, 117]. It might be expected that areas with chiggers would become gradually confluent, except for unsuitable lacunae of terrain, thus forming an endemic area or region [116]. In a recent extensive survey in Northwest Yunnan, China, chigger diversity was lower in the flatlands, but mean abundance and intensity was higher than in the mountains. *Leptotrombidium deliense* predominated in flatlands, and *L. scutellare* in mountains [118].

Ecotones may provide the conditions to allow both rodents and chiggers to thrive. One study of forest and open scrubland transects found the highest numbers of chiggers attached to rodents trapped in ecotones (three times more *L. deliense* than away from the ecotone) [102, 116]. Goff [119] in Papua New Guinea reported an abundance of *L. deliense* in disturbed habitat but not in undisturbed areas. A more detailed assessment collecting free-living chiggers using black plates placed on the ground, found chiggers to be more commonly associated with cleared areas in scrub habitat, along paths, fringes of scrub habitat and under trees and bushes [120]. Using a similar method on Hachijo Island, Japan, chiggers were located in damp areas in the transition between hills and flat areas and in forests near flat areas [93]. In Taiwan, a detailed assessment of ploughed and fallow habitats found three times more chiggers in the fallow fields. There was no association with rodent density or species, suggesting that the microhabitats of the fallow field, with more shade, leaf litter and shrubs provided more suitable habitat for chigger survival [121]. Porous, well drained, moist soil appears to be most suitable, but no detailed studies have been performed [56].

Certain plant species have often been cited as associated with scrub typhus, such as grasses including *Imperata cylindrica* (kunai grass in New Guinea, kogan grass in the Philippines, lalang grass in Malaya), *Saccharum spontaneum*, *Eleusine indica*, *Cyperus iria* and *Paspalum conjugatum* [53, 99–101, 122]. In the “*yudokuchi*” or noxious areas of northwest Honshu along riverbanks, *Miscanthus sinensis* and *Phragmites communis* are common [123]. However, Audy [116] concluded that detailed botanical surveys did not prove useful as no clear correlation between plant species and the disease emerged. He proposed a more synecological picture using the broader habitat groups described above and it is from here that the term “scrub” became synonymous with the disease.

Whether scrub typhus occurs in primary forest and has a sylvatic cycle that can “escape” to infect bordering chigger-mammal-chigger cycles and humans has been much debated [54]. Almost no studies of vectors and hosts have been carried out in what can be described as true primary forest. Traub et al. [113] reported positive isolation of *O. tsutsugamushi* from three rodents (*Rattus mulleri*, *R. edwardsi* and *R. rajah* group) as well as a pool of *Ascoschoengastia audyi* chiggers collected from a *Callosciurus* squirrel in primary Malayan jungle. Although Muul & Liat [124–126] also reported *O. tsutsugamushi* by isolation and serologically in forest rodents (*Rattus sabanus*) and a single squirrel *C. notatus*, the forest at Bukit Lanjan near Kuala Lumpur was not strictly primary. Even within rainforests, habitat can vary with small clearings due to fallen trees, paths and along the banks of rivers and streams [98]. These may provide the opportunity for increased densities of rodents and chiggers [116]. Forests tend to have greater small mammal diversity, but lower density, with each mammal associated with particular chigger species [55]. There is no definite evidence of humans acquiring scrub typhus in primary forest, given that very few humans live completely in undisturbed forest without altering it. A study of antibodies to scrub typhus in the Orang Asli tribes of Malaysia, found higher levels in those living in deep forest clearings compared to those on the forest edge or in villages [127]. However, it was impossible to say where the disease was acquired and whether the fringe habitats within the forest were important.

#### Seasonality: temperature, rainfall and humidity

The seasonality of human scrub typhus has been well described in several countries across Asia. In Japan, South Korea, Taiwan and northern parts of China the infection presents almost exclusively from spring until early winter. In Thailand, Burma and India disease has been described as most common from June until November, but present throughout the year. In Malaysia, the island of New Guinea and the Pacific Islands seasonality has been less clearly defined. To what extent this pattern is dependent on temperature, rainfall, humidity and resultant numbers of vector chiggers remains unclear. Traub & Wisseman [9] noted that all cases have occurred in either tropical or subtropical conditions and that no significant outbreaks have been reported during the dry season in India or central/southern Burma.

In more northern latitudes, some seasonality is to be expected, with winter temperatures dropping too low for chiggers to feed or chiggers overwintering on their hosts and hence not available to attach to humans [105]. Several studies have linked the disease to the presence of different species of chiggers at different times of the year, in different parts of a particular country. In South Korea, for example, *L. scutellare* numbers peak in autumn corresponding to the highest rates of human cases [128–133], although this was not seen in all investigations [134]. *Leptotrombidium pallidum* is more common in northern and eastern areas, where fewer cases are seen and thus *L. scutellare* is believed to be the key vector [135]. A similar pattern is reported from Japan, with *L. scutellare* and *L. pallidum* causing autumn-winter cases and *L. akamushi* summer cases [136–140]. Outbreaks in soldiers training on Mt. Fuji’s foothills were seasonal, despite the soldiers’ presence year round [114]. A recent summary of human scrub typhus in Japan over a 59-year period demonstrated major outbreaks in October to December, with smaller case numbers in May to June in southern prefectures, whereas northern and northeastern areas had highest rates in May-June and moderate numbers in October to December [141]. Similarly, in Shandong, China peak human cases corresponded to peak *L. scutellare* numbers [142]. In the Primorye region of Russian Siberia, *L. pavlovskyi* peaked in summer and is implicated in human cases [143]. In lower Burma and Manipur (India) the seasonal variation in numbers of *L. deliense* was reportedly similar to that of human cases [53, 54, 144] and in Thailand *L. deliense* was most abundant during the rainy season from April to December [145]. In Tropical North Queensland cases peaked from March to July, coinciding with the rainy season and the period immediately after the rains [146]. In Tamil Nadu, India, the highest incidence occurred from October to December, coinciding with peak chigger numbers, although chigger numbers did not fall greatly at other times of the year [147]. In Maharashtra, only minor seasonal variation was seen in *Suncus murinus* and *Rattus blandfordi* chigger indices, whereas *Rattus rattus rufescens* had lower rates overall and near absence of chiggers from April to June [148]. In Malaysia, however, no marked seasonality in either human or rodent infections was seen, with only a small decrease during the dry season [30, 125]. In the Pescadores Islands, Taiwan, many cases presented in military personnel from April until November. Here, *L. deliense* is the vector and chigger numbers fall to nearly zero in winter and a close correlation of chigger abundance with human infection was reported [149, 150]. Olson et al. [149] estimated a minimum requirement of 0.69 chiggers per shrew as the critical abundance needed to result in 1 human case per month.

The importance of temperature was investigated on the Pescadores Islands. Chiggers were recorded on rodents 12 days after the first 30 °C daytime temperature of the year and the first human case occurred 10 days later, although this varied year-to-year dependent on cold spells [151]. Others also reported a close correlation on the Pescadores between mean monthly temperature, chigger abundance and human cases, but not so with rainfall [152, 153]. In a more widespread study across Taiwan, human case incidence correlated well with overall chigger abundance, although surprisingly not with *O. tsutsugamushi* infected chigger abundance [154]. In Guangzhou, China, each 1 °C temperature rise corresponded to a 14.98% increase in the monthly number of human cases [155] or an odds ratio of 3.8 [156]. However a clear correlation of cases with temperature was not seen in two studies from South Korea and India [157, 158]. In both Japan and South Korea it has been clearly demonstrated that the monthly distribution of cases becomes more evenly distributed at more southerly latitudes [131, 159].

Audy’s [144] extensive investigations in India and Burma revealed that chigger abundance falls during the dry season. The proportion of rodents carrying *L. deliense* rose after rains began, but the mean number per rodent (chigger index) lagged behind by a few weeks [55]. In Malaya, using bait animals in a hyperendemic area, 10 times fewer chiggers attached to rodents during dry periods than wet [80]. In the same study, but using human volunteers, 70% became infected during wet periods compared to 5–29% in dry spells. However the picture was somewhat confused as more chigger pools tested positive during the period with the lower chigger index (of 12) compared to a high index of 304 [80]. In Thailand, chigger species diversity was higher in the dry season and human scrub typhus incidence correlated strongly with chigger diversity [160]. In more tropical climates, annual temperature variation is less marked and here rainfall may be more critical to chigger abundance and human disease [89, 150]. Where and how these two factors converge and interact in different regions is not fully understood. In Tropical North Queensland all but 1 human case was reported east of the 60 inch isohyet (where 1500 mm or more rain falls annually) [146, 161] and in Thailand chigger abundance and human cases are highest during the rainy season [59]. In a study transect in Malaya, chigger abundance could be maintained by sprinkling the ground with water after the rains had ceased [162].

Temperature and humidity are certainly important factors in chigger development. A minimum temperature is required for eggs to hatch and in hotter climates; chiggers are more active in cooler damp morning conditions and seek refuge from very high temperatures by entering soil as deeply as 18 cm below the surface [55, 89, 163–165]. Scrub typhus risk has also been associated with hours of sunshine, lower atmospheric pressure (associated with rainfall) and in some studies humidity [142, 155, 166].

The importance of a time lag between weather events (such as exceptionally heavy monsoonal rains) and human cases should not be underestimated and may reflect both rodent breeding success and the chigger life-cycle. In Guangzhou, after 4 months lag, every 10% increase in relative humidity was associated with 8.5% (95% CI: 2.7–14.5%) increased odds for infection, and a 1-unit increase in multivariate El Niño Southern Oscillation (ENSO) index between 2006 and 2014 was associated with a 23.6% increased odds of scrub typhus cases after a 5-month lag [156].

### Vectors

Trombiculid mites are considered to be the major vector of scrub typhus. The term chigger probably derives from the Spanish *chico* meaning small, and initially referred to the scrub-itch trombiculid mites of North America. Later the term became synonymous with all trombiculid mite larvae [167]. Chiggers belong to the family Trombiculidae, subclass Acari, class Arachnida and phylum Arthropoda [51]. There are over 3000 species of Trombiculidae present across almost the entire world. The identification of chiggers to the species level is technically challenging given their small size and the lack of accessible and updated taxonomic keys. Indeed, there has been much confusion over identification, with many genera and species names changing over time. Recent advances in identification using autofluorescence techniques and genetic barcoding of conserved *18S* rDNA or mitochondrial cytochrome *c* oxidase subunit 1 genes may pave the way to greater taxonomic clarity [39, 168]. Over 50 species of chiggers are known to bite humans and of these, 10 species have good evidence of transmitting *O. tsutsugamushi* to humans and a further 5 are possible vectors [51]. Only members of the genus *Leptotrombidium* are confirmed vectors to humans; among these *L. deliense*, *L. akamushi*, *L. arenicola*, *L. imphalum*, *L. scutellare*, *L. pallidum* and *L. pavlovskyi* are the most important [51].

Chiggers may be collected from hosts, using black plates or other objects placed on the ground, from the surface of boots of a standing person and from soil surface matter using Berlese or Tullgren funnels [56, 93, 163].

The prevalence of *O. tsutsugamushi* in chiggers is low, with a median per site reaching 18% in *L. pallidum*, but less than 10% in other key species using molecular diagnostic tools (Table 3). Free-living chiggers generally have lower percentage infection rates compared to engorged chiggers (Table 3). In one study, for example, 2.6% of engorged *L. deliense* collected from wild rodents were positive by DIF while just 1.1% of offspring from the same collection were positive (i.e. naturally infected) [59].

Santibáñez et al. [51] provides a recent detailed update on the role of chiggers as vectors of human pathogens and Stekolnikov [169] published an updated key to the genus *Leptotrombidium* in 2013.

It is likely that Trombiculidae are divided into the following groups: those that do not bite humans or carry *Orientia*; those that bite humans but do not carry *Orientia* (scrub-itch mites); those that carry *Orientia* but do not bite humans (possibly intrazootic); and those that both carry *Orientia* and bite humans [159]. Of the latter, there are few common species, perhaps due to their habitat preferences and likelihood of encountering humans. Nadchatram [170] attempted to classify chiggers into 7 ecological groups, of which group 1 are the red-orange coloured soil surface dwellers with a broad range of hosts including humans.

The possibility of other vectors of scrub typhus should not be ignored, particularly in areas distant to the classical scrub typhus “triangle”, including the United Arab Emirates and Chile. Several reports of ticks testing positive for *O. tsutsugamushi* have been published [45, 46] and recently for a non-chigger mite, *Ornithonyssus bacoti* [47]. *Orientia tsutsugamushi* has been shown to multiply in inoculated ixodid ticks [171]. Traub & Wisseman [9] cited Russian research that suggested *O. tsutsugamushi* survived in fleas for 11 days and could be transmitted by the flea bite, but details were lacking. The history of leech bites at the site of eschars in scrub typhus cases in Chile has prompted further investigation but no current evidence of leeches being vectors exists [19, 172]. Chiggers have been found to carry novel *Rickettsia*, *Anaplasm*a and *Borrelia* species, but to what extent these are pathogens or transmitted to humans is unknown [173, 174]. Evidence from China implicates trombiculid mites in the transmission of Hanataan virus [175]. It seems probable that arthropods that feed on rickettsiaemic hosts may be able to acquire *Orientia*, but the bacteria probably cannot cross the gut wall and onward transmission has not been documented.

### Hosts

There are two major groups of vertebrates that host chiggers; the “maintaining hosts” which comprise small mammals (rodents and shrews), ground-dwelling birds and “incidental hosts” (other birds and larger mammals including humans). Several reports provide detailed lists of animal hosts that reveal the enormous range of species that can be parasitized [9, 55, 176, 177]. Harrison & Audy [176] reported *L. deliense* from 87 species of host. Only monkeys, gerbils, hamsters and humans are thought to suffer clinically with scrub typhus [55, 178].

The patchy distribution of chiggers in the environment has already been alluded to, and will be discussed further. Maintaining hosts are able to acquire chiggers and either re-deposit them at the same site or a nearby site, whereby the intensity of this interaction (i.e. number of hosts) contributes to the abundance of mites and where *O. tsutsugamushi* is present, the risk of disease [55, 144]. Incidental hosts, such as birds and monkeys, may play a role in transporting chiggers to more distant sites and setting up new foci of infection.

It has been estimated that in ideal conditions, a single small mammal host could support four generations of chiggers per year, with a total of 10,000 feeding on that individual [55]. In Peninsular Malaysia, one *Rattus argentiventer* was estimated to support 4000 chiggers/month [179]. Individual rodents can host over 1000 chiggers at one time, tree shrews 5000 and ground-dwelling birds as many as 11,000 [55]. The rate of turnover of chiggers is an important factor for vector competence [102].

The pattern and degree of infestation of hosts is dependent on several factors. Host behaviour and habitat exploitation is of great importance. Terrestrial and scansorial mammals are more heavily infested and frequently infected with *O. tsutsugamushi* than arboreal mammals [9, 126, 180, 181]. More generalist rodents that exploit different types of habitats, such as *Rattus* species are often implicated in scrub typhus. Indeed, Traub & Wisseman [98] postulated that scrub typhus distribution closely mirrors the distribution of *Rattus* and *Leptotrombidium* species, with the place of *Rattus* taken by voles in Korea and Japan.

Vertebrate home range size may be important, with greater exposure to chiggers and the ability to host large numbers of ectoparasites with more extensive ranging [54, 59, 179]. However, data on home range sizes for rodents are relatively few. In Taiwan, studies of larger species (*Bandicota indica* and *Rattus losea*) reported not more than 500 m ranges [121]. At Changi Camp, Singapore, as much as 640 m was recorded for *Rattus* species [182]. On Shichito Island, Japan, re-trapping of *Rattus norvegicus* took place up to 50 m away, 50–60 m for mice and 25–30 m for voles [183]. In Malaysia, one *Rattus tiomanicus* was re-trapped 560 m away [184]. Vertebrate body size may also be important but data are inconsistent. Traub & Wisseman [52] report less than 20 chiggers attached to individual tigers, leopards, civets and deer, whereas small rodents and shrews may have thousands. Small terrestrial mammals may be best suited to acquiring chiggers, as they forage on and in the ground where their backs and ears are at the height of many species of questing chiggers. Small mammal burrows and nests may also be suitable locations for chiggers to attach.

The site of chigger attachment varies in different hosts. In rodents the ear fossae are typically parasitized, in the Tupaidae the midline and inguinal region, in shrews the perianal area and in macaques the eyelids and eyebrows [52]. Traub & Wisseman [52] suggested that host grooming efficiency may explain why mice frequently have few chiggers, whereas shrews have many. However, there appear to be few studies on grooming efficiency between different small mammal species. Other possible factors affecting attachment site include skin thickness, hair type, skin immune response and microclimatic conditions [170, 185, 186]. Some chigger species attach to specific areas of a host’s body, for example *Schoengastia schuffneri* has a predilection for rat groins [119, 122]. In humans, the attachment site is often associated with areas of pressure, for example the axillae or around the waistband [9].

There is some degree of host specificity among chiggers, although to what extent this is dependent on host contact with habitat-specific chigger species is unclear [187]. Some chigger species when presented with a human finger refuse to attach, but when presented with a bird readily attach and engorge [89].

Rodents, like humans in endemic areas, probably become infected repeatedly with *O. tsutsugamushi* during their lifetime. In the wild, captured *R. tiomanicus* were rickettsaemic for a mean of 97 days [184]. In the laboratory, rickettsaemia was recorded in *R. rattus* after a *Leptotrombidium* bite from day 7 until 8 weeks later [188]. In another study, viable *O. tsutsugamushi* were recovered from the kidney of a *Rattus annandalei* 4 months after being infected [181]. In an endemic setting, at least a 50% lifetime infection rate in rodents has been estimated [184]. *Orientia tsutsugamushi* yields from tissue are greatest from spleen, followed by liver and kidney. Lung tissue is also frequently positive, but results are inconsistent [78, 181, 189, 190].

The role of birds in the ecology and distribution of scrub typhus has been almost completely ignored. Several bird species have been reported as efficient hosts of vector chigger species. Ground-living birds such as quail are frequently heavily colonised, with 18,500 *T. akamushi* removed from 9 birds in Malaysia [54]. Others reported *L. deliense* and *T. pseudoakamushi* on Chinese quail (*Coturnix chinensis)*, Greater coucal (*Centropus javanicus* or *C. sinensis*), Raffles’ malkoha (*Rhinortha chlorophaea*) and Oriental reed warbler (*Acrocephalus orientalis*) [191, 192]. In Japan the green pheasant (*Phasianus colchicus tohkaidi* or *P. versicolor*) harboured *L. pallidum* [193]. Japanese scientists reported 72 species of birds carrying trombiculid mites, including *L. pallidum* and *L. scutellare*, but all attempts at isolating *O. tsutsugamushi* from these failed [194].

Kitaoka et al. [194] showed that chickens and pigeons could both be experimentally infected and remain rickettsaemic for as long as 42 days. The domestic chicken was reported to harbour both *L. scutellare* and *L. pallidum*. Kitaoka et al. [195] also exposed chickens to areas of high disease endemicity and was able to recover *O. tsutsugamushi* from a liver and spleen pool of 1 chicken and from *L. pallidum* attached to the chickens. The only records of wild birds carrying *O. tsutsugamushi* come from Khasansky District, Primorye, Russia and Xinjiang Province, China. In the Russian Far East, these were black-faced bunting (*Emberiza spodocephala*) and long-tailed rosefinch (*Carpodacus sibiricus*) tested using xenodiagnosis [196]. In China, 5/91 house sparrows (*Passer domesticus*) and 3/16 grey wagtails (*Motacilla cinerea*) tested positive from spleen tissue by PCR [41, 42].

These species (except the resident *Passer domesticus*) breed in Russia and northern China and migrate to South Korea, southern Japan, southern China, South and East Asia depending on the species [197]. Long-distance migrants arriving in Europe from Africa have been found to be carrying non-European mites [198]. Chiggers may be transported by birds in both local areas (ground-living birds) and over long distances (migrants). These could potentially set up new foci of disease and explain the extension of scrub typhus along island chains, for example East to the Solomon Islands and southwest to the Chagos Islands. This also raises the intriguing potential for further extension towards Madagascar and the African continent. However, recent reports of humans and dogs infected with *O. tsutsugamushi* in Chile would suggest a different mechanism of spread, as there are no known bird migration routes between Asia/Australia and Chile [199]. Human transportation of rodents through international trade and shipping has been implicated in the spread of many infectious diseases.

### Immunity and strain diversity

There has been very little research into the dynamics of antibodies to scrub typhus in either animal or human hosts. One study of rodents in Thailand demonstrated that IgG persisted for 10 months in animals removed from the wild and kept in captivity [59]. IgM was detected until week 6 in naturally infected *R. rattus* [188]. Studies in humans in the early 1950s clearly showed that even in those with recent natural scrub typhus infection, there was no significant protective effect to re-exposure by a different strain [200]. Homologous immunity was stronger, providing complete protection for at least 3 years [201]. Similarly, in cynomolgus macaques (*Macaca fascicularis*), homologous immunity was evident for at least 8 months, but not after 5–6 years [202].

Multiple strains may be present in a single colony of chiggers (*L. pallidum*) established from one individual [203]. Additionally, multiple strains can be maintained in an *L. imphalum* colony by transovarial transmission [57].

In humans, mixed infections identified by multi-locus sequence typing (MLST) comprised as much as 25% of cases in Thailand and 8.6% in Laos [204, 205]

Laboratory studies showed that transmission of strains from chiggers to rodents was variable, in that only the Karp strain was found in rodents fed upon by Karp and Gilliam-infected chiggers [206]. In Shandong Province, China, sequencing of the 56 kDa gene from rodents, chiggers and humans in one area suggested some consistency in key genotypes [207]; whereas in a study from 6 Thai provinces, a large diversity of genotypes was seen in humans with only 2 genotypes commonly distributed throughout the country [208].

### Mite islands

The concept of “mite islands” was first put forward by Audy [167] in the 1940s. The idea was based on the patchy distribution of human cases and of chiggers in the environment. The size of mite islands should be considered on two scales. At the largest scale, islands consist of endemic areas, ranging from a few square miles, to thousands of square miles, often bounded by major ecological barriers. At the small scale, an “island” of mites may be less than 30x30 cm and patchy over an area of hundreds of metres. The Japanese were well aware of this, describing localised high-risk areas as *yudokuchi* (“noxious area”). The patchiness may be more marked in drier climates, where mites are associated with damper areas. The islands may expand and contract over time, dependent on a myriad of factors [55, 116, 117, 159, 209]. Mite islands have been recorded in an enormous range of habitats from man-made wasteland, neglected cultivation and plantations to water meadows and forest edge [55, 117]. More unusual habitats include parks [210], walled vegetable gardens [211] and subalpine meadows [24]. There have not been reports from very arid habitats or deep forest.

The presence of a mite island is probably dependent on factors such as suitable climatic conditions, species composition and stability of the vertebrate population, intensity of the vertebrate–chigger–host interactions, food supply (for larvae and adults), soil, microclimate and chigger predators [54, 55]. The role of small mammals in the small-scale dynamics of mite islands is of importance. Rodents probably forage on similar routes and thus chiggers can be picked up and returned to suitable habitats on a regular basis. Even within the same areas, two rodents of the same species can have markedly different numbers of chiggers attached. Additionally, a rodent removed from its home range for three days and then replaced will very quickly acquire the chiggers that would have attached in the intervening time [212]. Rodent burrows have been reported to yield large numbers of free-living chiggers [56].

Several studies demonstrate the patchy distribution of chiggers in nature. In a transect through *Imperata cylindrica* grass in Malaysia using rabbits or rats as bait animals or black plates to collect free living chiggers, numbers varied at different points along the transects. Variation also occurred over time and the highest numbers were seen at the start of the rainy season [80, 88, 213]. In these studies, no clear link to habitat type was identified, although in another study negative areas were more frequently on clay soil [214]. Another study collecting wild rodents in similar habitat in an area of 1300 m^2^ could find no clustering of *O. tsutsugamushi* infected rodents [215]. In Japan, studies using sentinel voles (*Microtus montebelli*) also reported the patchy distribution of *O*. *tsutsugamushi* infected voles and a changing pattern over time [210, 216, 217]. Most positive sites were positive on more than one occasion over the course of 3 years [218]. In chemoprophylaxis studies with humans, individuals seated on grass 45 cm apart had significantly differing numbers of chiggers attached, which was consistent to the location if two people changed position [88].

The differences between endemic and non-endemic areas have also been examined in a few cases. In Japan, 50% *L. pallidum* tested positive for *O. tsutsugamushi* in an endemic area (of human disease) and only 3.8% in a non-endemic area [219]. At the site of an intense outbreak of disease in plantation workers on the Goodyear Estate, Deli, Sumatra, 50.5% of rats were infested with mites with a chigger index of 104, whereas on two nearby estates without outbreaks, 6.3% and 2.3% of rats were infested with indices of 7 [192]. The study by Ishikura et al. [219] certainly suggests that even areas thought to be non-endemic for scrub typhus, but occurring within an endemic region, may not be completely free of risk. Our understanding of this remains poor.

The dynamics of mite island size and distribution over time are also not well understood. Some hyperendemic sites have become low to no-risk sites over the course of several years [54, 220]. It is likely that the factors mentioned above as important for the establishment of a mite island may change as, for example, a plantation matures resulting in less rodent food availability and different microclimatic conditions less suitable for vector chigger species [9].

### Land use, climate change and disease risk

The importance of changing climate, habitats and environment on the risk of infectious diseases is becoming increasingly recognised [221–224]. There has been little research into these impacts for scrub typhus. The multiple impacts of humans on habitat, such as through shifting cultivation and clearing of forests, has been linked to the creation of fringe and scrub type habitats that are thought to favour more intense host–chigger–host cycles. Some countries with the infrastructure to record scrub typhus incidence have reported declines in human cases (Japan [225] and Taiwan [226]), whereas others have seen increases (South Korea [227] and China [228]). The reasons behind these changes are not well understood. Saito et al. [229] suggested that the fall in incidence in Japan could be partly due to less river flooding as a result of widespread dam building. Kuo et al. [121] in Taiwan reported that the abandonment of rice growing fields following Taiwan’s joining of the World Trade Organisation in 2001, led to an increase in suitable chigger habitat which had been controlled by regular ploughing. Surprisingly, however, the rates of *O. tsutsugamushi* infection in small mammals in fallow and ploughed fields were not statistically different despite much higher numbers of chiggers in fallow areas. In South Korea, a recent study suggested that climate change might be partly responsible for the northern expansion of the key vector *L. scutellare* [39].

### Humans

#### Focalization of human cases

The concept of mite islands as described above first came about as a result of many observations of focal outbreaks among soldiers in WW2. Classic examples include an outbreak of 756 cases in Ceylon after 4 days’ exposure [114] and in Sansopor, Dutch New Guinea, in which 931 cases with 34 deaths occurred over 1 month, with almost all cases linked to abandoned village gardens and plantations [122, 230]. In Finschhafen, New Guinea, reoccupation of a camp abandoned just 6 weeks previously resulted in 16 cases and 6 deaths, all of who were involved in clearing grass to make an outdoor cinema [100]. Plantation workers were also affected in well-described focal outbreaks. Cases often occurred in groups of workers detailed with clearing specific areas of the plantation [191, 231, 232]. The importance of shifting cultivation and the resultant exposure to potentially high risk habitats was also noted [116]. There has been a steep rise in reports published of scrub typhus outbreaks in the last decade, particularly in India and China [1]. This is most likely due to a combination of increasing awareness, land use changes from expanding human populations and climate change with more extreme weather events. A striking aspect of many of the reports on outbreaks is that those exposed (e.g. military personnel, plantation workers) were immunologically naïve to scrub typhus [232–235].

#### Human behaviour and disease risk

In scrub typhus endemic areas, disease risk varies over the course of the year, with fewer human cases during winter periods in northern latitudes and dry periods in tropical latitudes. To acquire a chigger bite, exposure to suitable habitat is required. Occupational risk is important, with the main burden of disease in farmers, plantation workers and the military [53, 55, 146, 231]. There have been increasing cases due to recreational exposure in suburban parks and gardens [39, 236].

Certain activities, such as lying or sitting directly on the ground are particularly hazardous [117]. A recent detailed analysis of farming practice between a high incidence area in South Korea and a low incidence area of Japan with similar environmental and climatic features and the same vector (*L. scutellare*) identified certain risk factors. Working longer hours in the field, more bending forward or squatting at work, resting on the ground, lack of protective clothes and less washing of clothes and skin were all statistically significantly associated with scrub typhus infection [237, 238]. A separate study of farmers in South Korea identified water around the home, dry field farming and working with livestock as significant risk factors [238]. Ecological niche modelling using maximum entropy algorithm has recently been used to predict scrub typhus occurrence across China, based on data from Jiangsu Province [239].

#### Interventions to reduce risk

No vaccine currently exists to prevent scrub typhus. Vaccine development was first attempted and trialled at the end of WW2, with some protection provided to naturally acquired scrub typhus in a small number of test cases [240, 241]. However, fully vaccinated military personnel were not prevented from acquiring severe disease [242]. Further attempts in Japan in the 1940s also failed to provide lasting immunity [243]. The Japanese successfully used the mild Pescadores strain of *O. tsutsugamushi* to demonstrate protection from a virulent strain [244]. Valbuena et al. [245] provide a more detailed account of the history of vaccine development. However, a number of other preventive measures can be taken and these have been summarised recently by Xu et al. [1]. Avoiding high-risk areas and reducing the chance of a mite bite by avoiding sitting or lying directly on the ground are important. The use of insecticides on skin and clothing has been shown to be effective in several studies during WW2 [56, 93, 99, 101, 117]. Physical removal of attached mites is impractical due to their small size, although Malay mothers were reported to remove chiggers from their children with a needle [178]. Thorough washing of skin and clothes with a detergent after outdoor activities is recommended. Clearing suitable habitat for chiggers may reduce risk in the long term. Similarly, rodent control, although challenging to accomplish in practice, can reduce disease risk, although conversely the immediate disease risk may increase, with a large population of chiggers suddenly without their usual food source. For short-term, high-risk activities such as soldiers on operation or field workers, there is some evidence that weekly doxycycline may be effective prophylaxis [246]. Public engagement with regard to the measures described above is likely to be very useful. The application of mathematical modelling to strategies to reduce scrub typhus risk in humans by Min et al. [247] suggested that reducing contact between humans and chiggers is more effective than attempting to either control rodents or chiggers.

## Discussion

Studies investigating *O. tsutsugamushi* in vectors and non-human hosts span almost a century and nearly 30% of the literature is published in a language other than English. Prior to 1924, there were a few studies in Japan where mites were allowed to feed on monkeys and an eschar and illness compatible with scrub typhus was recorded; however, no laboratory tests were performed to identify the pathogen in these studies [248–250].

Studies were performed in 30 countries and at least 793 locations. Approximately 40 different types of laboratory tests were used to identify *O. tsutsugamushi*. These tests were combined into eight categories to analyse available data. Seventy-four “vector” species were tested and 46 were positive, including Ixodida (ticks), Macronyssidae and Laelapidae (mites). Depending on the laboratory test category, overall positivity ranged from 0.6 to 5% for individual vectors and 9.6 to 56% for pooled vectors. *Leptotrombidium deliense* was most frequently positive across South and Southeast Asia, while *L. pallidum* and *L. scutellare* were key vector species in South Korea and Japan. Among free-living chiggers, 31 species were tested with 23 species positive. Almost all of these were *Leptotrombidium* species. It is assumed that many species of ectoparasite that feed on a host with active *O. tsutsugamushi* infection may be capable of acquiring the pathogen. However, as trombiculid mites usually feed just once in their life-cycle, successful transovarial and transstadial transmission is required to act as a vector. No evidence exists to suggest that Ixodida or other non-trombiculid mites are capable of onward transmission. Thus, *Leptotrombidium* species appear to be best equipped to act as vectors, although the reasons for this are unknown. A large number of hosts were tested, amounting to 234 species that included mostly small mammals, but also Artiodactyla, birds and lizards. In all 122 species tested positive, ranging from 2 to 40% positive overall depending on the laboratory test category. The Muridae were both the most frequently tested and *O. tsutsugamushi-*positive.

In addition to the 276 articles included in the systematic review, 145 other articles were reviewed to summarise knowledge of key themes in scrub typhus ecology. Topics reviewed include the chigger life-cycle, feeding behaviour and role of hosts including birds in the disease ecology. Although the disease is termed “scrub” typhus, data are lacking to clearly link the risk of human infection to this somewhat vague habitat type. Higher risk areas within endemic areas probably exist and are likely to be related to the presence of suitable habitat for chigger species that transmit the pathogen to humans together with sufficient maintaining hosts. Our understanding of the extent of such areas and dynamics over time is very limited.

The findings of Audy and colleagues [31, 54, 116, 144] on the Indo-Burma border 70 years ago and their description of mite islands reflected an unusual relationship between humans and the ecology of *O. tsutsugamushi* and this may have led to an overemphasis on the importance of mite islands in the subsequent literature. In WW2, many young soldiers with zero previous exposure to scrub typhus were suddenly exposed at high human density to small areas of territory in extremely basic living conditions, a form of ‘rural mass gathering’. In today’s world, such mass short-term naïve human invasions of small areas of rural tropical environments are rare and typically occur in immunologically naïve military personnel engaged in activities with high exposure risk.

If military tactical decisions led to deposition of these high densities of naïve humans in areas of high *O. tsutsugamushi*-infected chigger density, the heterogenous distribution of *O. tsutsugamushi* would have highlighted the importance of mite islands that may be less important in stable dispersed rural communities. Hence, mite islands may be of less public health importance today, even though they may still exist in some biotypes, as large numbers of people are not deposited in them and people may pass through mite islands relatively infrequently. The lack of rural mass gatherings such as occurred in WW2 would also make it much harder to detect putative mite islands today as relatively few people would be exposed and their ranging patterns are likely to be large. This does not mean that mite islands are not important as greater understanding may help prevent the severe public health issues that occurred in the 1940s, in case of future ‘rural mass gathering’ arising from military action and training, civilian camping and orienteering, and refugee crises with urban people seeking shelter in rural environments.

### Study limitations

Articles included in this review are of widely varying quality. Very few articles provide a comprehensive account of all potentially useful data collected (Table 6). Some studies chose to focus on either vector or host, providing minimal details on the group not under investigation. Trombiculid mites are notoriously difficult to identify to species level, with limited and often out-of-date taxonomic keys available. Many authors identified trombiculid mites to genus only, and it is likely that misidentification was frequent. Furthermore, much of the taxonomy of vectors (and some hosts) has changed over the long time-course of scrub typhus research. For this review, species names known to be the same were combined and the current taxonomy used.

**Table 6:** Summary of proposed minimum data recording and reporting standards for studies investigating *O. tsutsugamushi* in vectors and non-human hosts

Topic	Checklist item	Notes
Dates	Vector and host collection dates	Month(s) and year(s). Where collections are made monthly, data should be reported monthly to inform seasonality of disease
Location	Study site GPS coordinates and administrative divisions 1 to 4^a^	Spelling, transliteration and changes over time can be avoided by using GPS coordinates
Sample collection and storage method	Hosts trapping technique. Vectors collection method. Sample storage medium and conditions	For collection of free-living vectors, provide details on methods^c^
Host species	All hosts should be identified to genus and species	Reference up to date nomenclature
Vector species	All vectors should be identified to genus and species where possible^b^	Record identification method and reference up to date nomenclature
Laboratory methods	Provide details of laboratory methodology	Where several methods are used, ensure data clearly separated by test
Sample type	List	Where several sample types tested, separate results. Where tissues are pooled, provide details
Testing hosts	Numbers tested and numbers positive	Avoid pooling samples from different hosts (and species). Avoid pooling samples from different sites
Testing vectors	Numbers tested and numbers positive. If pooled give approximate number or range per pool	Avoid pooling samples from different sites
Host vector infestation rate	Proportion of hosts infested by the vector. Divide by host species and location	If possible, provide vector species composition
Host vector index (chigger index: average number of chiggers/host)	Vector (chigger) index. Divide by host species and location	If possible, provide vector species composition
Ecology	Habitat description; mean, minimum and maximum temperature; rainfall; humidity; soil type	Note differences between study sites where applicable
Study ethics	Provide details of appropriate animal handling and euthanasia (if used) protocol and approval	Local, national and international standards may apply

^a^Administrative division categorization varies widely from country to country. The equivalent of province, district, sub-district and town or village should be given

^b^As a minimum, genus must be recorded. For pooled vectors, the majority of constituent genus/species should be recorded

^c^Black plate, Berlese or Tullgren funnels or other techniques

An enormous number of combinations of data were presented in the literature. For example: vectors and hosts collected from different locations were combined for laboratory analysis; multiple host and vector species were pooled for testing; several laboratory tests and multiple samples types were combined for diagnosis. It was not practical to extract and analyse the same data in different combinations. Many studies presented some results without a denominator (i.e. number positive given without number tested). In the analysis, the total number tested obviously excludes studies where the number was not provided; however, the number testing positive was included. Therefore, slightly lower positivity rates should be expected. Where data was available for study location, but species not provided, this was collected in favour of the species names if the locations were combined. The location of positives was considered a more useful outcome measure as almost any animal host may become infected when bitten by an infected chigger.

In order to identify articles for inclusion, very broad search terms to account for the terminology of scrub typhus and ecology were required. Additionally, many references were cross-checked. It is possible that some articles were not captured. Only a single person performed article selection and data extraction for most articles. For non-English articles, extraction was done together with native speakers, to ensure all data was extracted as accurately as possible. Only six articles were excluded, as the full-text could not be found. An article reviewing Chinese research into scrub typhus and two papers from Japan, including Tamiya’s 300-page “Recent advances in studies on Tsutsugamushi disease in Japan” reveal that there are additional local, governmental and military reports and monographs that were not identified through our extensive literature search [26, 136, 251].

### Proposed minimum data recording and reporting standards

As described in previous sections, many articles reporting the presence of scrub typhus in vectors and non-human hosts fail to provide many useful data and other key pieces of information. It is acknowledged that for some studies, certain information may not be available, or may be beyond the scope of the study.

As with other branches of medical research (https://www.ndorms.ox.ac.uk/research-groups/equator-network), in order to improve our understanding of the ecology of scrub typhus it will be vital that future research becomes more standardised in design and reporting. Table 6 lists all recommended information to be recorded in all studies, where relevant. This checklist may also be applicable to studies collecting vectors and hosts for laboratory testing of other pathogens.

Future studies should also ensure that they make use of the “Strengthening of reporting of observational studies” (STROBE) checklist [252] and “Checklist for One Health Epidemiological Reporting of Evidence” (COHERE) [253].

### Major gaps in our knowledge of scrub typhus ecology

Although useful research and important advances in our understanding of the ecology of scrub typhus has taken place, much of this took place prior to the 1970s and little research has re-examined existing dogma.

The life-cycle of trombiculid mites suggests that chiggers act as both vector and host. However, whether these laboratory studies are applicable in nature is not well understood. How *O. tsutsugamushi* may influence the biology of the vector and the dynamics of the pathogen through the life stages are similarly poorly understood. The hypothesis that chiggers infected by feeding on bacteraemic hosts apparently do not effectively pass on the pathogen transovarially raises important questions about the reservoir of the disease, the population genetics and impressive strain diversity that is well recognized. This may be explained, to some extent, by the existence of co-feeding chiggers, but very few studies have investigated this phenomenon. Further attempts at the challenging studies required to establish whether chiggers feed more than once in nature during their life-cycle are also much needed. The mechanisms behind the apparent ability of *O. tsutsugamushi* to manipulate the sex ratio of offspring, with very high proportions being female allowing onward transovarial transmission is poorly understood. Could *O. tsutsugamushi* be improving its chances of survival by increasing the proportion of females in the population? This phenomenon is well recognised in *Wolbachia* and in some *Rickettsia* species (all α-proteobacteria) [66].

*Leptotrombidium* species appear to be the major vector of scrub typhus, but to what extent other genera or even other ectoparasites could be involved is unknown. Why are *Leptotrombidium* species the key vector? Recent identification of scrub typhus in Chile, parts of Africa and the Middle East suggest that *Orientia* species are present across much of the tropics and subtropics. Why is human disease limited to a few case reports across this region? The disease frequently presents as an undifferentiated fever and may be overlooked, particularly in regions where it is little known. In areas where the disease is emerging, local strains may cause mild or sub-clinical illness or perhaps competent vector species are not present.

The term “scrub typhus” suggests a particular habitat type. However, it is clear that the infection can be found in many different habitat types. Mite islands were well described during WW2, but this was mostly based on human outbreaks rather than systematic survey of vectors and hosts. As revealed above, there were very few study sites reporting no positives, and most of these (except Brazil) were close to sites with positives. What factors govern the presence of the disease in an area? Is the disease widely distributed in endemic areas with hot spots related to suitable habitat for vector chigger life-cycle and a sufficiently high small mammal population? The scale and dynamics of any such hotspots or “mite islands” is not understood. What is the role of birds in scrub typhus ecology? Birds are known to harbour vector chigger species and wild birds have tested positive for *O. tsutsugamushi*. It seems possible that birds may allow the disease to be distributed over wider areas and potentially for new hot spots to be set up. Which bird species are most important? It is likely that ground dwelling birds with large home ranges or migrant species that spend time foraging on the ground are most suited to distributing infected chiggers. The role of migratory birds in distributing ticks carrying infectious diseases has been widely reported [254, 255].

The seasonality of the disease in humans is well described. The factors that control this are less clear. It is expected that beyond an uncertain northern latitude, the influence of temperature is most important, with winters too cold for chiggers to be active. In more southern latitudes, rainfall is probably more critical with chigger numbers being higher after the rains have started. Human activity and exposure risk should not be underestimated. Chiggers will not attach to humans unless given sufficient opportunity. Some recent studies have begun to investigate farming practices and behaviours associated with the risk of disease.

### Possible tools to begin to unravel these

Advances in molecular tools over the past three decades, including quantitative PCR and nested PCR, allow more robust diagnostics with high specificity. Whole-genome sequencing will provide invaluable insight into the complex population genetics and structure of *O. tsutsugamushi* in vectors, hosts and humans that may help understand both the pathogenicity and ecological interactions. The same technologies could also much improve our taxonomic understanding of trombiculid mites. Regrettably there are very few and decreasing number of experts in the morphological taxonomy of trombiculid mites. To our knowledge, only a single functional chigger colony currently exists for the laboratory study of many of these important and unanswered questions and developing new colonies would be of great benefit.

Access to powerful geospatial data to map infected and uninfected vectors and hosts and link these with habitat, land use and the environment will help develop our understanding of the geographical extent of the disease and the factors that influence it. Niche modelling methods could be used to predict regions where *O. tsutsugamushi* may be unrecognised as has been performed for other infectious diseases [256]. Long-term climate and climate change data may allow us to predict the risk of disease and the factors that govern this. Studies on migrating birds could provide useful data on the dynamics of infection and establishment of new risk areas. All these in combination with computational power and statistical modelling techniques will advance our understanding of the ecology and disease risk.

## Conclusions

The known global distribution of scrub typhus is expanding into new regions and human activity is having a profound impact on the globe. Understanding the ecology of this important neglected disease has crucial implications for our development of strategies to mitigate human infection. This systematic review provides a comprehensive assessment of current knowledge and highlights key areas for future research.

## Supplementary information

**Additional file 1: Table S1.** PRISMA checklist. **Table S2.** Laboratory tests and test categories. **Table S3.** List of study sites, GPS coordinates and administrative level. **Table S4.** Numbers of vector genera and species tested for *O. tsutsugamushi* by combined laboratory tests. **Table S5.** Complete list of trombiculid mites and other Acari tested for *O. tsutsugamushi*, with GPS coordinates, laboratory test used and reference. **Table S6.** Numbers of host genera and species tested for *O. tsutsugamushi* by combined laboratory tests. **Table S7.** Total and median testing *O. tsutsugamushi* positive by laboratory test category for the most frequent host species. **Table S8.** Median chigger index by host species. **Table S9.** Median percentage host chigger infestation by host species.

## Data Availability

All data generated or analysed during the present study are included in this published article and its additional file.

## Abbreviations

DALYs: disability adjusted life years
rRNA: ribosomal ribonucleic acid
DIF: direct immunofluorescence
IIF: indirect immunofluorescence
CF: complement fixation
ELISA: enzyme-linked immunosorbent assay
PCR: polymerase chain reaction
WW2: World War 2
kDa: kilo Dalton
ENSO: El Nino-Southern Oscillation
rDNA: ribosomal deoxyribonucelic acid
MLST: multi-locus sequence typing
IgM: immunoglobulin M
IgG: immunoglobulin G
