# Supplementary material for: Scrub typhus ecology: a systematic review of Orientia in vectors and hosts
Source: Parasit Vectors. 2019 Nov 4;12:513. doi: 10.1186/s13071-019-3751-x (PMC6829833; doi:10.1186/s13071-019-3751-x)
Supplement: Supplementary file 1 — Additional file 1: Table S1. PRISMA checklist. Table S2. Laboratory tests and test categories. Table S3. List of study sites, GPS coordinates and administrative level. Table S4. Numbers of vector genera and species tested for O. tsutsugamushi by combined laboratory tests. Table S5. Complete list of trombiculid mites and other Acari tested for O. tsutsugamushi, with GPS coordinates, laboratory test used and reference. Table S6. Numbers of host genera and species tested for O. tsutsugamushi by combined laboratory tests. Table S7. Total and median testing O. tsutsugamushi positive by laboratory test category for the most frequent host species. Table S8. Median chigger index by host species. Table S9. Median percentage host chigger infestation by host species. [file 13071_2019_3751_MOESM1_ESM.pdf]

PRISMA 2009 Checklist

Section/topic	#	Checklist item	Reported on page #
**TITLE**			
Title	1	Identify the report as a systematic review, meta-analysis, or both.	1
**ABSTRACT**			
Structured summary	2	Provide a structured summary including, as applicable: background; objectives; data sources; study eligibility criteria, participants, and interventions; study appraisal and synthesis methods; results; limitations; conclusions and implications of key findings; systematic review registration number.	1-2
**INTRODUCTION**			
Rationale	3	Describe the rationale for the review in the context of what is already known.	2-3
Objectives	4	Provide an explicit statement of questions being addressed with reference to participants, interventions, comparisons, outcomes, and study design (PICOS).	3
**METHODS**			
Protocol and registration	5	Indicate if a review protocol exists, if and where it can be accessed (e.g., Web address), and, if available, provide registration information including registration number.	3-6
Eligibility criteria	6	Specify study characteristics (e.g., PICOS, length of follow-up) and report characteristics (e.g., years considered, language, publication status) used as criteria for eligibility, giving rationale.	3-4
Information sources	7	Describe all information sources (e.g., databases with dates of coverage, contact with study authors to identify additional studies) in the search and date last searched.	4
Search	8	Present full electronic search strategy for at least one database, including any limits used, such that it could be repeated.	4-5
Study selection	9	State the process for selecting studies (i.e., screening, eligibility, included in systematic review, and, if applicable, included in the meta-analysis).	5
Data collection process	10	Describe method of data extraction from reports (e.g., piloted forms, independently, in duplicate) and any processes for obtaining and confirming data from investigators.	5-6
Data items	11	List and define all variables for which data were sought (e.g., PICOS, funding sources) and any assumptions and simplifications made.	5-7
Risk of bias in individual studies	12	Describe methods used for assessing risk of bias of individual studies (including specification of whether this was done at the study or outcome level), and how this information is to be used in any data synthesis.	6
Summary measures	13	State the principal summary measures (e.g., risk ratio, difference in means).	6
Synthesis of results	14	Describe the methods of handling data and combining results of studies, if done, including measures of consistency (e.g., I^2) for each meta-analysis.	6-8

PRISMA 2009 Checklist

Section/topic	#	Checklist item	Reported on page #
Risk of bias across studies	15	Specify any assessment of risk of bias that may affect the cumulative evidence (e.g., publication bias, selective reporting within studies).	6
Additional analyses	16	Describe methods of additional analyses (e.g., sensitivity or subgroup analyses, meta-regression), if done, indicating which were pre-specified.	6-8
**RESULTS**			
Study selection	17	Give numbers of studies screened, assessed for eligibility, and included in the review, with reasons for exclusions at each stage, ideally with a flow diagram.	8
Study characteristics	18	For each study, present characteristics for which data were extracted (e.g., study size, PICOS, follow-up period) and provide the citations.	8-11
Risk of bias within studies	19	Present data on risk of bias of each study and, if available, any outcome level assessment (see item 12).	-
Results of individual studies	20	For all outcomes considered (benefits or harms), present, for each study: (a) simple summary data for each intervention group (b) effect estimates and confidence intervals, ideally with a forest plot.	8-27
Synthesis of results	21	Present results of each meta-analysis done, including confidence intervals and measures of consistency.	8-27
Risk of bias across studies	22	Present results of any assessment of risk of bias across studies (see Item 15).	8-27,61
Additional analysis	23	Give results of additional analyses, if done (e.g., sensitivity or subgroup analyses, meta-regression [see Item 16]).	
**DISCUSSION**			
Summary of evidence	24	Summarize the main findings including the strength of evidence for each main outcome; consider their relevance to key groups (e.g., healthcare providers, users, and policy makers).	58-68
Limitations	25	Discuss limitations at study and outcome level (e.g., risk of bias), and at review-level (e.g., incomplete retrieval of identified research, reporting bias).	61-62
Conclusions	26	Provide a general interpretation of the results in the context of other evidence, and implications for future research.	58-68
**FUNDING**			
Funding	27	Describe sources of funding for the systematic review and other support (e.g., supply of data); role of funders for the systematic review.	68

From: Moher D, Liberati A, Tetzlaff J, Altman DG, The PRISMA Group (2009). Preferred Reporting Items for Systematic Reviews and Meta-Analyses: The PRISMA Statement. PLoS Med 6(7): e1000097. doi:10.1371/journal.pmed1000097

For more information, visit: **www.prisma-statement.org**.

Additional file 1: Table S2. Summary of laboratory test categories and list of tests for each category

Molecular	Serological	Combination serological & molecular	Culture +- microscopy	Combination culture & serological	Combination culture & molecular	Microscopy
56 kDa PCR	Direct immunofluorescence (DIF)	ELISA & PCR	Xenodiagnosis	Xenodiagnosis & complement fixation	Xenodignosis & PCR	Organ impression smears
47 kDa PCR	Indirect immunofluorescence (IIF)	56 kDa, nested PCR & sequencing	Xenodiagnosis & microscopy	Xenodiagnosis & OXK/Weil Felix	Xenodiagnosis & L929 cell culture & PCR	Giemsa stain or unspecified
GroEL PCR	ELISA	IF & PCR		Xenodiagnosis & DIF/IIF	L929 cell culture & PCR	
Nested PCR	OXK/Weil Felix test			Xenodiagnosis & L929 cell culture & IIF		
16s rRNA sequencing	Complement fixation			Xenodiagnosis & unspecified serological method		
Unspecified molecular method	Passive haemagglutination assay					
	Indirect Immunoperoxidase					
	Unspecified serological (IgM/IgG) method					
	Unspecified antigen-based method					

Additional file 1: Table S3. List of all study sites and administrative level (accuracy score)

Site id	Province name	Latitude	Longitude	Country	Site name	Administrative level§
1	Addu Atoll	-0.6333	73.1667	Maldives	Port X	2
2	Unknown	14.0583	108.2772	Vietnam	Unknown	0
3	Akita	39.2106	140.5147	Japan	Omono River, Jumonji	2
4	Shizuoka	35.2944	138.8536	Japan	East Fuji Maneuvre Area, Kitago 5km NE Higashi-Fuji, Oshima 6km NE Higashi-Fuji	2
5	Quebec	47.0320	-70.6280	Canada	Grosse Isle	3
6	Anhui	32.5746	118.1267	China	Sanjie	4
7	Fujian	26.6628	118.1926	China	Ningde, Nanping, Sanming, Pucheng	1
8	Guangdong	21.6635	110.9242	China	Maoming	2
9	Henan	32.1470	114.0910	China	Xinyang City	2
10	Shandong	36.7270	117.1502	China	Jinan Suburbs	2
11	Hebei	36.9942	113.9278	China	Taihang Mountains	3
12	Shandong	35.8940	117.9249	China	Unknown	1
13	Fujian	26.6656	119.5479	China	Ningde	2
14	Inner Mongolia	49.2009	119.7646	China	Hulun Buir area	2
15	Hebei	38.0371	114.4687	China	Unknown	1
16	Xinjiang	44.8539	82.0510	China	Bole	3
17	Xinjiang	43.4329	83.3803	China	Nalati Grassland	4
18	Jiangsu and Shandong	35.0480	118.6540	China	Multiple	0
19	Guangdong	23.2573	117.2880	China	Nanpeng	4
20	Shandong	35.2660	117.9773	China	Fei County	3
21	Jilin	42.8628	130.3660	China	Hunchun	3
22	Shandong	36.2558	117.1056	China	Mount Tai	4
23	Fujian	25.5441	118.7595	China	Unknown	1
24	Zhejiang	30.5833	119.8333	China	Hangzhou	2
25	Shandong	36.6686	117.0204	China	Fei County	3
26	Beijing	39.9040	116.4075	China	Beijing	1
27	Xinjiang	44.0221	89.5940	China	Changji	3
28	Fujian	24.4400	118.3300	Taiwan	Kinmen County	3
29	Guangdong	20.9167	110.6000	China	Naozhou	4
30	Oro	-8.7620	148.3666	Papua New Guinea	Dobadura	4
31	Queensland	-16.3830	145.3350	Australia	Whyanbeel Parish, west of Dayman Point state school	4
32	Queensland	-16.4190	145.3900	Australia	Portion 252 east of road to Daintree	4
33	Queensland	-16.3610	145.3560	Australia	Portion R100 northern boundary of Portion 14v	4
34	Maharastra	18.7546	73.4062	India	Lonavala	4
35	Maharastra	18.4454	73.7801	India	Khadakvasla	4
36	Maharastra	18.3839	73.6036	India	Panchet	4
37	Maharastra	18.3663	73.7559	India	Singhgadh	4
38	Maharastra	18.5699	73.8506	India	Khadki	4
39	Maharastra	18.4771	73.8907	India	Kondhwa (Khurd)	4
40	Maharastra	18.5911	73.9188	India	Lohegaon	4
41	Maharastra	18.5580	73.8075	India	Aundh	4
42	Maharastra	18.5024	73.8785	India	Pune Cantt	4
43	Maharastra	18.4923	73.8703	India	Ram Tekri	4
44	Fujian	25.4790	119.7429	China	Beicuo, Pingtan Island	4
45	Anhui	32.7771	117.9892	China	Mingguang	3

46	Unknown	35.8617	104.1954	China	Unknown	0
47	Udon Thani	17.3647	102.8159	Thailand	unknown	1
48	Chiang Mai	18.7061	98.9817	Thailand	Unknown	1
49	Chiang Rai	19.9105	99.8406	Thailand	Unknown	1
50	Nonthaburi	13.8591	100.5217	Thailand	Unknown	1
51	Khon Kaen	16.4322	102.8236	Thailand	Unknown	1
52	Prachaup Kirikhan	11.8124	99.7973	Thailand	Unknown	1
53	Sa Kaeo	13.8240	102.0646	Thailand	Unknown	1
54	Tak	16.8840	99.1259	Thailand	Unknown	1
55	Ubon Ratchathani	15.1264	105.4302	Thailand	Chong Mek	4
56	Petchaburi	12.9649	99.6426	Thailand	Unknown	1
57	Guangdong	22.0371	113.9172	China	Wanshan Archipelago	4
58	Guangdong	23.4308	117.0925	China	Nan'ao	3
59	Guangdong	20.9142	110.0966	China	Leizhou	3
60	Xinjiang	43.8347	90.2860	China	Mori Kazakh Autonomous County	3
61	Fujian	25.2211	119.4754	China	Nanri	4
62	Yunnan	25.6391	101.0065	China	NE of Xundian Hui & Yi counties, NW of Yulong Naxi county & south of Simao district	1
63	Queensland	-16.4500	145.3733	Australia	Mossman	4
64	Taiwan strait	24.6581	119.6979	Taiwan	Pescadores Islands	1
65	Ardennes region	49.6807	4.7961	France	Unknown	2
66	Unknown	16.1534	-14.9208	Senegal	Senegal River	1
67	Loei, Nan, Buriram	17.1430	101.8700	Thailand	Multiple	0
68	Chiang Rai	20.4414	99.9289	Thailand	Viang Hom	4
69	Chiang Rai	20.4166	99.9911	Thailand	Rong	4
70	Chiang Mai	19.7370	98.9734	Thailand	Aruno Tai	4
71	Chiang Mai	19.7422	98.9370	Thailand	Muang Na	4
72	Mae Hong Son	18.1702	97.9305	Thailand	Mae Sa Riang	4
73	Mae Hong Son	18.8093	98.0326	Thailand	Mae Ja Ton	1
74	Tak	16.6898	98.5177	Thailand	Rim Mei	4
75	Tak	16.7096	98.5054	Thailand	Wang Ta Kian	4
76	Ratchaburi	13.5334	99.5809	Thailand	Bo Moo	1
77	Ratchaburi	13.6545	99.2103	Thailand	Tago Lang	4
78	Phetchaburi	12.9465	99.6194	Thailand	Teng Nuea	1
79	Phetchaburi	12.9465	99.6194	Thailand	Suan Yai Pattana	1
80	Prachuap Khiri Khan	11.7758	99.6911	Thailand	Dan Sing Khon	4
81	Prachuap Khiri Khan	11.2938	99.3765	Thailand	Klong Loi	4
82	Kanchanaburi	14.5822	99.0501	Thailand	Tai Mhuang	1
83	Kanchanburi	14.5822	99.0501	Thailand	Pra Chum Mai	1
84	Chumphon	10.9192	99.2214	Thailand	Ran Tad Phom	4
85	Chumphon	10.8415	99.0386	Thailand	Santi Nimitr	4
86	Ranong	9.9666	98.7005	Thailand	Nai Krung	1
87	Ranong	10.5980	98.8101	Thailand	Had Tun	4
88	Chiang Rai	19.8480	99.7070	Thailand	Ban Mae Sad	4
89	Xinjiang	42.5246	87.5396	China	Unknown	1
90	Jiangsu	32.8684	120.3203	China	Dongtai	3
91	Fujian	24.4456	118.0827	China	The Wan Shi Botanical Gardens, Siming, Xiamen	4
92	Samut Prakan	13.5991	100.5998	Thailand	Unknown	1
93	Bangkok	13.7563	100.5018	Thailand	Unknown	1
94	Nonthaburi	13.8136	100.4227	Thailand	Amphoe Bang Kruai	2

95	Samar	12.2446	125.0388	Philippines	Unknown	1
96	Phit Sanulok	16.7512	100.3114	Thailand	31st Border Patrol Police Base	4
97	Nonthaburi	13.8333	100.5167	Thailand	Tambon Tha Khanun, Bangkruai	4
98	Shizuoka Prefecture	35.3219	138.8817	Japan	Takigahara Army Base, Gotemba	4
99	Pahang	3.1706	102.3192	Malaysia	Bukit Mendi	4
100	Jakarta	-6.1330	106.8267	Indonesia	Ancol	4
101	Guangdong	23.6818	113.0560	China	Qingyuan	2
102	Sonsorol	5.3268	132.2239	Palau	Unknown	1
103	Koror Island	7.3411	134.4772	Palau	Unknown	1
104	Fengping	23.5849	121.5026	Taiwan	Unknown	3
105	Fujian	25.4614	119.7204	China	Pingtan Island	4
106	Biak Island	-1.2000	136.0600	Indonesia	Sorido	4
107	Baik Island	-1.1500	136.0300	Indonesia	Insrom	4
108	Biak Island	-1.1500	136.2160	Indonesia	Adibat	4
109	Biak Island	-0.9330	136.0000	Indonesia	Sarwom	4
110	Biak Island	-0.9166	136.0500	Indonesia	Korem	4
111	Owi Island	-1.2500	136.2100	Indonesia	Unknown	4
112	Unspecified	31.9162	59.6281	Iran	Multiple	0.5
113	Shizuoka	35.2941	138.8537	Japan	Mt. Fuji	2
114	Queensland	-16.9260	145.7280	Australia	Cairns	3
115	Queensland	-17.0860	145.8290	Australia	Unknown (Near Cairns)	2
116	Hanoi	20.9938	105.8467	Vietnam	Truong Dinh	4
117	Hanoi	21.0039	105.8403	Vietnam	Dong Da	4
118	Hanoi	21.0220	105.8486	Vietnam	Ha Dong	4
119	Hanoi	20.9909	105.8650	Vietnam	Mai Dong	4
120	Hanoi	20.9836	105.8422	Vietnam	Giap Bat	4
121	Hanoi	21.0133	105.8657	Vietnam	Huu Nghi	4
122	Selangor	3.1833	101.6000	Malaysia	Bukit Lanjan	4
123	Selangor	2.9411	101.5264	Malaysia	Bukit Kemandul	4
124	Selangor	3.1517	101.9097	Malaysia	Ulu Lui	4
125	Selangor	3.3639	101.6029	Malaysia	Serendah	4
126	Selangor	2.8469	101.6725	Malaysia	Bukit Tampoi	4
127	Selangor	2.8896	101.7440	Malaysia	Bukit Tunggal	4
128	Selangor	2.9048	101.7732	Malaysia	Kampung Bahagia	4
129	Selangor	2.8092	101.6267	Malaysia	Bukit Tadom	4
130	Selangor	3.2106	101.5052	Malaysia	Elmina Estate	4
131	Selangor	3.0742	101.5703	Malaysia	Kuala lumpur metroplitan	4
132	Selangor	3.1725	101.7085	Malaysia	Jalan Fletcher	4
133	Selangor	3.1279	101.5945	Malaysia	Petaling Jaya	4
134	Selangor	3.1384	101.7668	Malaysia	Ampang New Village	4
135	Selangor	3.0886	101.7083	Malaysia	Salak South New Village	4
136	Selangor	3.1980	101.5682	Malaysia	Jinjang North New Village	4
137	Selangor	3.2452	101.6586	Malaysia	Selayang Baharu	4
138	Toyama	36.8177	137.5980	Japan	A (Kurobe River)	4
139	Toyama	36.8377	137.5857	Japan	B (Kurobe River)	4
140	Toyama	36.9571	137.5404	Japan	C (Kurobe River)	4
141	Toyama	36.8942	137.4852	Japan	D (Kurobe River)	4
142	Toyama	36.9154	137.4235	Japan	E (Kurobe River)	4
143	Toyama	36.8637	137.6046	Japan	F (Kurobe River)	4

144	Toyama	36.6656	137.1989	Japan	G (Zinzu River)	4
145	Toyama	36.6494	137.1416	Japan	H (Yamada River)	4
146	Toyama	36.7814	137.0570	Japan	I (Oyabe River)	4
147	Toyama	36.7493	137.1101	Japan	J (Gejo River)	4
148	Gifu	35.4387	137.0446	Japan	Kani City to Sakahogi (Kiso river)	3
149	Gifu	35.5558	137.0446	Japan	Kuze town (Ibi river)	4
150	Gyeonggi-do	35.5558	136.5071	South Korea	Chipo-ri	4
151	Gyeonggyi-do	38.2496	127.4197	South Korea	Kumhwa	4
152	Gyeonggyi-do	37.8321	127.1390	South Korea	Songu-ri	4
153	Gyeonggyi-do	38.1817	127.3523	South Korea	Monkey-ri	4
154	Gyeonggyi-do	38.0964	127.0748	South Korea	Yonchon	3
155	Gyeonggyi-do	38.0988	126.9070	South Korea	Ko-wang ni	4
156	Gyeonggyi-do	38.1014	126.9842	South Korea	Unspecified near 38th parallel	3
157	Pahang	4.4430	102.3848	Malaysia	Lata Berkoh, Taman Negara National Park	4
158	Suraburi	14.5289	100.9101	Thailand	Unknown	1
159	Chaiyaphum	15.8068	102.0315	Thailand	Unknown	1
160	Bangkok Metropolitan	13.7664	100.5486	Thailand	Unknown	1
161	Chantaburi	12.6112	102.1038	Thailand	Unknown	1
162	Assam	27.3284	95.8352	India	Stilwell Road, 9 sites between 4.6 and 22 miles	3
163	Kachin	25.4423	97.3517	Myanmar	Camp Landis	3
164	Kachin	25.3948	97.2031	Myanmar	Myitkyina, 15mi West	3
165	Manipur	24.6640	93.9063	India	Unknown	1
166	Uttarakhand	29.6817	79.8588	India	Kumaon	2
167	Jammu & Kashmir	34.3916	73.7728	India	Teetwal	4
168	Shizuoka	35.3218	138.8971	Japan	Gotenba-Oyama district (11 sites rodents, 17 sites free-living chiggers)	1
169	Chiang Rai	20.2244	99.9481	Thailand	Ban Pa Gook (4 sites around village)	4
170	Chiang Rai	19.8480	99.7070	Thailand	Ban Mae sad (4 sites around village)	4
171	Seoul	37.5333	126.9833	South Korea	Yongsan Garrison	4
172	Unknown	-2.5489	118.0149	Indonesia	Unknown	0
173	Unknown	4.4106	109.6181	Malaysia	Unknown	0
174	Gyeonggi-do	38.0781	127.0769	South Korea	Firing Point 10, Yeoncheon-gun	3
175	Akita	39.4370	140.4970	Japan	Omagari area	2
176	Hokkaido	43.0583	141.4764	Japan	Nopporo Area	4
177	Akita	39.2148	140.5213	Japan	Jumonji area along Omono river	3
178	Selangor	2.9411	101.5264	Malaysia	Bukit Kemandul	4
179	Niigata	37.7445	139.1826	Japan	Gosen	3
180	Kanagawa	35.1442	139.6208	Japan	Miura	3
181	Shizuoka	35.3606	138.7278	Japan	Mt Fuji foothills	3
182	Shizuoka	34.6511	138.8585	Japan	Minami-Izu	4
183	Oita	33.3036	131.2678	Japan	Hijudai	3
184	Oita	33.5633	131.7323	Japan	Kunisaki	3
185	Mpumalanga	-24.6500	31.3330	South Africa	Bushbuckridge Municipal Area	3
186	Bengal	22.7674	88.3883	India	Barrackpore	4
187	Selangor	3.1891	101.5585	Malaysia	Oil Palm Estate	1
188	Chiang Rai	22.2245	99.9481	Thailand	Ban Pa Gook	4
189	Chiang Rai	20.2106	99.9302	Thailand	Ban Nongkroung	4
190	Hua-lien	23.8085	121.4848	Taiwan	Shou-feng & Fong-lin lowlands	2
191	Unknown	23.6958	121.0940	Taiwan	9 sites around Taiwan	0
192	Yilan	24.5656	121.6425	Taiwan	Unknown	1

193	Hualien	23.7479	121.3813	Taiwan	Unknown	1
194	Lanyu Island	22.0436	121.5484	Taiwan	Unknown	3
195	Taitung	22.8567	121.0407	Taiwan	Unknown	1
196	Taoyuan	24.9004	121.2627	Taiwan	Unknown	1
197	Taichung	24.2335	120.8977	Taiwan	Unknown	1
198	Kaoping	22.5779	120.4534	Taiwan	Unknown	1
199	Matsu	26.1846	120.0545	Taiwan	Unknown	3
200	Kinmen	24.4518	118.3681	Taiwan	Unknown	3
201	Hualien	23.9230	121.5660	Taiwan	Ji-an & Shou-feng villages	2
202	Singapore	1.3569	103.9721	Singapore	Changi camp	2
203	Gyeonggi	37.2340	127.7070	South Korea	Songsan, Hwaseong	4
204	Gyeonggi	37.2877	127.0181	South Korea	Jangan, Hwaseong	4
205	Gyeonggi	37.2980	127.6372	South Korea	Yeoju	2
206	Gyeonggi	37.7599	126.7802	South Korea	Paju	2
207	Gangwon	38.1466	127.3132	South Korea	Cheorwon	1
208	Gangwon	37.8813	127.7300	South Korea	Chuncheon	1
209	Chungbuk	36.9910	127.9259	South Korea	Chungju	1
210	Jeollanam	35.2025	127.4627	South Korea	Gurye	1
211	Gyeongnam	35.5666	128.1658	South Korea	Hapcheon	1
212	Jeollanam-do	35.6019	127.5021	South Korea	Hwangjeon-myeon, Suncheon-si	3
213	Gaafu Dhaalu atoll	0.2900	73.4571	Maldives	Gadhdhoo island	3
214	Selangor	3.1714	101.6188	Malaysia	Village, Bukit Lanjan forest reserve	4
215	Selangor	3.1752	101.6164	Malaysia	Lalang, Bukit Lanjan forest reserve	4
216	Selangor	3.1738	101.6152	Malaysia	Scrub/edge, Bukit Lanjan forest reserve	4
217	Selangor	3.1716	101.6117	Malaysia	Forest, Bukit Lanjan forest reserve	4
218	Tainan	23.1487	120.3324	Taiwan	Unknown	1
219	Shandong	35.2861	118.1846	China	Feixian County, Fangcheng town, along Jun river	4
220	Queensland	-17.1816	145.4551	Australia	Rocky Creek	4
221	Queensland	-17.3632	146.0184	Australia	Bramston Beach	4
222	Queensland	-17.5226	146.0285	Australia	Innisfail	4
223	Queensland	-17.6074	145.7812	Australia	Palmerston	4
224	Queensland	-17.5145	145.6044	Australia	Millaa Millaa	4
225	Queensland	-17.6092	145.4836	Australia	Ravenshoe	4
226	Queensland	-18.1924	145.5992	Australia	Kirrama	4
227	Queensland	-18.6494	146.1625	Australia	Ingham	4
228	Queensland	-21.1425	149.1821	Australia	Mackay	4
229	Himachal Pradesh	30.9009	76.9651	India	Kasauli	4
230	Himachal Pradesh	30.9079	76.9629	India	Pasteur Institute Estate	4
231	Himachal Pradesh	30.9021	76.9811	India	Garkhal	4
232	Himachal Pradesh	39.9762	76.9898	India	Subathu	4
233	Guangdong	23.3306	116.5557	China	Shantou	2
234	Unknown	15.0428	100.7789	Thailand	18 Provinces	0
235	Gyeonggi	37.8920	126.7223	South Korea	North Carolina Range: Jangdan-myeon, Paju-si	4
236	Gyeonggi	37.9208	126.7458	South Korea	Warrior Base: Gunnae-myeon, Paju-si	4
237	Gyeonggi	37.8956	126.8024	South Korea	Monkey range: Jindong-myeon, Paju-si	4
238	Gyeonggi	37.9541	126.8048	South Korea	Story range: Jindong-myeon, Paju-si	4
239	Gyeonggi	37.9673	126.8408	South Korea	Dagmar North: Jeokseong-myeon, Paju-si	4
240	Gyeonggi	38.0487	127.1050	South Korea	Firing Point 60: Yeoncheon-gun	4
241	Gyeonggi	38.0179	126.7195	South Korea	Rodriguez Range: Youngjung-myeon, Pocheon-gun	4

242	Unknown	23.7387	120.9489	Taiwan	Unknown	0
243	Puducherry & Tamil Nadu	12.0217	79.6672	India	12 villages	3
244	Khasan	42.4299	130.6776	Russia	River valleys & lakes	2
245	Taitung	22.0262	121.5526	Taiwan	Iratai village, Lanyu Island	4
246	Taitung	22.0503	121.5133	Taiwan	Ya-yu village, Lanyu	4
247	Taitung	22.0375	121.5664	Taiwan	Ivarinu village, Lanyu	4
248	Taitung	22.0278	121.5430	Taiwan	Imurud village, Lanyu	4
249	Taitung	22.0580	121.5644	Taiwan	Iraramuruk village, Lanyu	4
250	Taitung	22.0788	121.5273	Taiwan	Iraralai village, Lanyu	4
251	Primorye	42.8720	131.3654	Russia	Khasan & Slavyan districts	1
252	Khatlon	37.4707	69.3423	Tajikistan	Parhar, Kyzylzu river bank	2
253	Khatlon	37.2180	69.1062	Tajikistan	Panj, beside Panj river	2
254	Vahdat	38.7470	69.3004	Tajikistan	Romit Reserve along Sardai Miyona river	2
255	Heilongjiang	47.0571	127.8267	China	Qing'an county	3
256	Fujian	24.4028	117.7898	China	Longhai county	3
257	Guangdong	23.2322	113.3658	China	Guangzhou suburbs	2
258	Hainan	16.3298	112.0255	China	Xisha Islands	3
259	Da Nang	16.1246	108.2681	Vietnam	Tien Sha peninsula	3
260	Quang Nam	15.7555	108.1943	Vietnam	Hill 244	2
261	Manus	-2.8483	146.2316	Papua New Guinea	South Bat Island	4
262	Zhejiang	29.1795	120.0888	China	Unknown	1
263	Yunnan	24.9760	101.4894	China	Unknown	1
264	Tibet	29.3208	95.3250	China	Medog county	3
265	Hunan	28.6003	109.9955	China	Guzhang county	3
266	Maharashtra	21.1456	79.0855	India	Nagpur	3
267	Maharashtra	21.0779	78.9672	India	Raipur	4
268	Shandong	35.5934	119.5334	China	Huangdao, Qingdao County	3
269	Shandong	35.3619	118.0899	China	4 villages in NE Fei County	3
270	Shandong	35.9424	117.9120	China	4 counties in Shandong Province	2
271	Selangor	2.8968	101.7290	Malaysia	Jendaram	4
272	Selangor	3.1337	101.5528	Malaysia	Subang	4
273	Shandong	37.1668	117.8339	China	Zouping County	3
274	Shandong	35.8794	117.9282	China	Fei & Zouping counties	2
275	Chiba	34.9074	139.8991	Japan	Shirahama	4
276	Tokyo	33.1030	139.8036	Japan	Hachijo Island	3
277	Niigata	37.4463	138.8513	Japan	Nagaoka	3
278	Kochi	33.7200	133.5500	Japan	Ida	1
279	Kochi	33.7200	133.5500	Japan	Okata	1
280	Hokkaido	43.2212	142.8634	Japan	Unknown	1
281	Aomori	40.7932	141.2362	Japan	Tohoku	4
282	Unknown	36.1621	139.6951	Japan	Kanto Region	0.5
283	Unknown	36.1386	137.8299	Japan	Chubu Region	0.5
284	Unknown	34.7372	135.5941	Japan	Kinki Region	0.5
285	Unknown	34.7741	132.8621	Japan	Chugoku Region	0.5
286	Unknown	33.7525	133.6426	Japan	Shikoku Island	0.5
287	Unknown	32.6444	130.9559	Japan	Kyushu Island	0.5
288	Kyoto	35.5500	135.1300	Japan	Nodagawa river	3
289	Sabah	6.0495	116.6862	Malaysia	Poring, Primary Forest	4
290	Sabah	6.0498	116.7008	Malaysia	Poring, Secondary Forest	4

291	Sabah	6.0494	116.7035	Malaysia	Poring, Edge Habitat	4
292	Selangor	3.2501	101.6000	Malaysia	Bukit Lagong	4
293	Selangor	3.3548	101.8391	Malaysia	Janda Baik	4
294	Anhui	33.0323	117.0323	China	Huaiyuan county	3
295	Anhui	32.7880	116.5675	China	Fengtai county	3
296	Anhui	30.5984	117.8947	China	Qingyang county	3
297	Oro	-9.1313	148.6919	Papua New Guinea	Buna	3
298	Torres Islands	-13.2497	166.6167	Vanuatu	Unknown	2
299	Banks Islands	-13.5257	167.3267	Vanuatu	Ureparapara	4
300	Banks Islands	-13.6794	167.6708	Vanuatu	Mota Lava	4
301	Banks Islands	-13.8443	167.4472	Vanuatu	Vureas Bay, Vanua Lava	4
302	Gaua	-14.2810	167.5161	Vanuatu	Unknown	4
303	Espiritu Santo	-15.3004	166.9182	Vanuatu	Unknown	1
304	Guadalcanal	-9.5773	160.1456	Solomon Islands	Unknown	1
305	Makira	-10.5737	161.8097	Solomon Islands	Unknown	2
306	Ndende (Nendo Island)	-10.7685	165.8211	Solomon Islands	Head of Graciosa Bay	4
307	Ndende (Nendo Island)	-10.7238	165.7987	Solomon Islands	Government Station	4
308	Ndende (Nedno Island)	-10.7987	165.8395	Solomon Islands	Lueselemba Bay	4
309	Ndende (Nendo Island)	-10.7234	165.8336	Solomon Islands	East side of Graciosa Bay	4
310	Banks Islands	-13.8325	167.5833	Vanuatu	Grouped	3
311	Ndende (Nendo Island)	-10.7224	165.9234	Solomon Islands	Grouped	3
312	Delhi	28.4841	77.1879	India	Unknown	3
313	Unknown	36.2060	138.2530	Japan	Unknown	0
314	Mato Grosso do Sul	-18.9833	-56.6500	Brazil	Nhumirim ranch, Pantal region	4
315	Maharashtra	18.4560	73.5502	India	6 sites	1
316	Maharashtra	18.3716	73.7607	India	Sinhgarh	3
317	Maharashtra	17.9278	73.6493	India	Mahabaleshwar	3
318	Oita	32.9666	131.4000	Japan	Taketa	3
319	Oita	33.1990	131.5170	Japan	Hasama	3
320	Oita	32.2330	131.6060	Japan	Oita City	3
321	Fujian	26.6403	118.6822	China	Fu zhou, ping tan, putian and jian yang	1
322	Unknown	15.4993	102.6419	Thailand	Various sites	0
323	Da Nang	16.0782	108.0712	Vietnam	Unknown, Da Nang	1
324	Chittoor District	13.4566	79.0039	India	Chittoor district, Andhra Pradesh	2
325	North Andaman	13.2643	92.9827	India	Diglipur	4
326	South Andaman	11.7550	92.6558	India	Port Blair	3
327	Gyeonggi Province	37.9798	126.8512	South Korea	Dagmar North Training Area	4
328	Chonnam Province	35.1932	126.8291	South Korea	Gwangsangu and Bukgu, Gwangju Metropolitan area	1
329	Seoul	37.4615	126.9652	South Korea	Mt Gwanak	4
330	Selangor	3.0876	101.6146	Malaysia	Sungei Way Rubber plantation	3
331	Selangor	3.0801	101.5626	Malaysia	Subang at 7mile mark of Batu Tiga-Subang Road	3
332	Pampanga	15.1038	120.4450	Philippines	SW of Clarke Air Base	2
333	East Java	-7.9858	112.5943	Indonesia	Mulyorejo, Malang	4
334	East Java	-7.9995	112.6151	Indonesia	Bandungrejosari, Malang	4
335	East Java	-7.9745	112.6268	Indonesia	Klojen, Malang	4
336	Chiang Mai	18.9366	98.8229	Thailand	Ban Ponyeang	4
337	Chonburi	13.2445	101.5045	Thailand	6 sites in Bothong district	2
338	Chonburi	13.1869	101.5436	Thailand	Ban Vangri	4
339	Chonburi	13.2323	101.4560	Thailand	Ban Thaprang	4

340	Chonburi	13.2356	101.4592	Thailand	Ban Thapsung	4
341	Gifu	35.2831	136.5478	Japan	Yoro foothills	3
342	Phangnga	8.6710	98.4221	Thailand	unknown	1
343	Cheju Island	33.4805	126.3836	South Korea	Kumdogri, Aiwol-ub	4
344	Cheju Island	33.4876	126.4164	South Korea	Tonggui- ri	4
345	Cheju Island	33.4770	126.5520	South Korea	Ara-i-dong	3
346	Cheju Island	33.4477	126.5396	South Korea	Odong-dong	3
347	Cheju Island	33.3904	126.4854	South Korea	Eorimog	4
348	Cheju Island	33.4156	126.4336	South Korea	Kosong-ri	4
349	Cheju Island	33.5015	126.4974	South Korea	Unknown	1
350	Unknown	4.0448	102.1634	Malaysia	Peninsular Malaysia, site unknown	0.5
351	Khon Kaen	16.2127	102.5201	Thailand	Mancha Khiri	4
352	Khon Kaen	16.6164	102.0962	Thailand	Chum Phae	4
353	Khon Kaen	16.7191	101.8715	Thailand	Phu Pha Man	4
354	Khon Kaen	16.2025	102.7664	Thailand	Ban Haet	4
355	Khon Kaen	15.9238	102.4581	Thailand	Waeng Yai	4
356	Khon Kaen	16.4919	102.6181	Thailand	Ban Fang	4
357	Goyang-gun	37.6805	126.8391	South Korea	Dorai 5-ri - cannot find	2
358	Goyang-gun	37.6704	126.8415	South Korea	Goyang-ri	4
359	Goyang-gun	37.6496	126.8705	South Korea	Wonhung 2-ri	4
360	Goyang-gun	37.6278	126.8390	South Korea	Haingsin 1-ri	4
361	Paju-gun	37.7793	126.8502	South Korea	Kwangtan 3-ri	4
362	Gyonggi-do	37.5094	126.7666	South Korea	Bucheon-si	4
363	Yesan-gun	36.7018	126.6699	South Korea	Deogsan-ri	4
364	Cheongwon-gun	36.4897	127.4110	South Korea	Simog-ri	4
365	Chollanam-do	34.9972	127.0435	South Korea	Hwasun	2
366	Chollanam-do	35.2053	126.7138	South Korea	Won-dong, Kwangju	3
367	Chollanam-do	35.1904	126.9101	South Korea	Wuchi-dong, Kwangju	3
368	Chollanam-do	35.2053	126.7138	South Korea	Bia-dong, Kwangju	3
369	Chungchongnam-do	36.6111	129.2980	South Korea	Yongi	2
370	Chungchongnam-do	36.4645	127.1212	South Korea	Kongju	1
371	Chungchongnam-do	36.3968	127.3479	South Korea	Tan-dong, Taejon	3
372	Chon-nam	35.1732	126.8592	South Korea	Kwangju	2
373	Chon-nam	34.6725	126.9252	South Korea	Changhung	2
374	Chon-nam	34.7725	127.6621	South Korea	Yocheon	2
375	Chon-bug	35.9597	126.9954	South Korea	Iksan	2
376	Chon-bug	36.0287	126.9292	South Korea	Iri	2
377	Kyong-nam	34.8508	128.4287	South Korea	Tongyong	2
378	Kyong-bug	36.4537	129.3798	South Korea	Yongdeok	2
379	Kangwon	37.9272	127.7415	South Korea	Chunseong	2
380	Kangwon	37.4486	129.1646	South Korea	Samcheok	2
381	Kangwon	37.5364	129.1147	South Korea	Tonghae	2
382	Kyonggi	37.6829	126.8396	South Korea	Koyang	2
383	Niigata	37.9153	139.1468	Japan	Kawamura Nakasu	4
384	South	35.2137	128.5814	South Korea	Masan	2
385	Gyonggi-do	37.9056	127.0287	South Korea	Jeonggam-dong, Dongducheon-si	3
386	Gyonggi-do	37.5093	126.7669	South Korea	Bugog-dong, Bucheon-si	3
387	Kyonggi	37.9123	127.2065	South Korea	Pochon	3
388	Kyonggi	37.0126	127.1146	South Korea	Pyongtaek	2

389	Kyonggi	37.8065	127.0484	South Korea	Yangju	2
390	Kyonggi	37.8807	127.2060	South Korea	Yongjongdo	3
391	Kyong-buk	35.9040	129.2269	South Korea	Kyongju	2
392	Kyong-buk	36.6823	128.8704	South Korea	Kumrung	1
393	Kyong-buk	36.1749	128.1115	South Korea	Kimchon	2
394	Kyong-buk	36.4549	128.1717	South Korea	Sangju	2
395	Kyong-buk	37.0233	129.3883	South Korea	Uljin	3
396	Kyong-buk	36.6731	128.4585	South Korea	Yechon	3
397	Kyong-buk	36.6136	128.1937	South Korea	Munkyong	2
398	Kyong-buk	36.6180	128.7380	South Korea	Andong	3
399	Kyong-nam	35.5604	128.5022	South Korea	Changnyong	3
400	Kangwon	38.6686	128.3185	South Korea	Kosong	4
401	Kangwon	38.4542	128.4546	South Korea	Keojin	4
402	Choong-nam	36.2114	127.1027	South Korea	Nonsan	2
403	Choong-nam	36.9205	126.6337	South Korea	Dangjin	2
404	Choong-nam	36.6088	126.6634	South Korea	Hongseong	3
405	Choong-buk	36.4920	126.5537	South Korea	Cheongwon	3
406	Chollanam-do	35.2687	127.0237	South Korea	Damyang	2
407	Chon-nam	34.6417	126.7738	South Korea	Kangjin	2
408	Cheju	33.4623	126.3276	South Korea	Aewol	3
409	Cheju	33.4760	126.5450	South Korea	Ara	3
410	Niigata	38.1120	138.3930	Japan	Sado Island, Umezu district	2
411	Chon-buk	35.9367	127.2575	South Korea	Wanju	2
412	Kyongsangbuk-do	36.2967	128.0972	South Korea	Cho-o 1, Sangju-si	4
413	Kyongsangbuk-do	36.3056	128.0977	South Korea	Cho-o 2, Shangju-si	4
414	Kyongsangbuk-do	35.9520	128.0155	South Korea	Yobae, Kumrung-gun	4
415	Kyongsangbuk-do	36.0244	128.0457	South Korea	Mipyong, Kumrung-gun	4
416	Kyongsangbuk-do	36.1122	127.9946	South Korea	Unsu, Kimchon-si	4
417	Kyongsangbuk-do	36.5482	128.7296	South Korea	Jeokha, Andong-si	4
418	Kyongsangbuk-do	36.5186	128.8652	South Korea	Imha, Andong-si	4
419	Kyongsangbuk-do	36.7390	128.0858	South Korea	Jinan, Munkyong-si	4
420	Kyongsangbuk-do	36.7335	128.1557	South Korea	Koyo, Munkyong-si	4
421	Chungchongbuk-do	36.9487	127.9101	South Korea	Tanwol, Chungju-si	4
422	Chungju-si	36.9125	127.9618	South Korea	Sesong, Chungju-si	4
423	Kyongsangbuk-do	36.6177	128.5004	South Korea	Jiksan, Yechon-gun	4
424	Unknown	12.8890	121.7700	Philippines	unknown	0
425	Pahang	4.5020	101.3900	Malaysia	Cameron Highlands	2
426	Johor	1.6680	103.7850	Malaysia	Johor several sites	1
427	Negri Sembilan	2.5160	102.2390	Malaysia	Unknown	1
428	Selangor	3.2640	101.5880	Malaysia	6 sites	1
429	Nakhon Ratchasima	14.9573	102.1106	Thailand	Unknown	1
430	Ubon Ratchathani	15.1832	105.1129	Thailand	Unknown	1
431	Sialkot	32.4972	74.5361	Pakistan	Sialkot	2
432	Sakhalin Oblast	49.9402	143.6239	Russia	Sakhalin & Shikotan (Kuril Islds.)	1
433	Maharashtra	19.1275	72.8811	India	Bombay City	3
434	Sakhalin Oblast	43.7948	146.7470	Russia	Shikotan Island	3
435	Sakhalin Oblast	44.1499	145.8750	Russia	Kunashir Island	3
436	Choallanam-do	35.3490	126.4030	South Korea	Yongkwang	2
437	Chollanam-do	35.2950	127.2940	South Korea	Koksung	2

438	Queensland	-17.4151	145.4365	South Korea	Wondecla	4
439	Chollanam-do	34.8097	127.1826	South Korea	Posong	2
440	Chollanam-do	35.1017	126.5444	South Korea	Hampyong	2
441	Hong Kong	22.3224	114.1353	Hong Kong	Stonecutters Island	3
442	Osaka	34.7172	135.5056	Japan	Osaka City	2
443	Kerala	8.6622	76.8093	India	Khizuvalam	4
444	Kerala	8.6799	76.8240	India	Mamom	4
445	Kerala	8.6599	76.8217	India	Kochalummoodu	4
446	Kerala	8.4939	76.9536	India	Rajaji Nagar	4
447	Chollanam-do	35.0033	127.3139	South Korea	Cheogye	3
448	Gyeongnam-do	35.4541	128.5805	South Korea	Gangjeon	2
449	Chon-nam	35.2001	126.5018	South Korea	Yongwang (Chonbul)	3
450	Chon-nam	35.2178	126.3232	South Korea	Yongkwang (Wolya)	3
451	Chon-nam	35.2643	126.4764	South Korea	Yongkwang (Gunso)	3
452	Gyenoggi	37.8353	126.8894	South Korea	North, Twin Bridges Training Area, Paju county	3
453	Gyeonggi	37.8101	126.8793	South Korea	Twin Bridges Training Area, South, Paju county	3
454	Xinjiang	45.6730	84.5820	China	Altai & Yili areas	1
455	Shandong	35.7457	117.9245	China	Feixian, Junan & Jinan counties	2
456	Yamagata Prefecture	38.3090	140.1495	Japan	Asahi-Machi	4
457	Saitama	35.9846	139.0898	Japan	Hitsujiyama Park	4
458	Kagoshima	31.5830	130.4660	Japan	Uwatoko, Matsumoto Town	4
459	Kagoshima	31.7000	130.6160	Japan	Kamihazeyama, Aira Town	4
460	Kagoshima	31.5000	130.4330	Japan	Imakoba, Fukiage Town	4
461	Buri Ram	15.1024	103.0970	Thailand	Unknown	1
462	Chachoengsao	13.7860	101.0920	Thailand	Unknown	1
463	Chiang Rai	20.2500	99.9386	Thailand	Changchawa sub-district, Mae Chan district	3
464	Phitsanulok	16.7597	100.2713	Thailand	Ekatosort Army Base	4
465	Phitsanulok	16.7554	100.2913	Thailand	Combined data, Ekatorot & 31st Border police bases	3
466	Aichi Prefecture	35.2275	137.3027	Japan	Okura area, Obara Village	4
467	Aichi Prefecture	35.2252	137.3285	Japan	Okura and Hirahata area, Obara Village	3
468	Saitama Prefecture	36.1894	139.5183	Japan	Site number 1	4
469	Saitama Prefecture	35.8731	139.5749	Japan	Site number 2	4
470	Saitama Prefecture	36.0594	139.4739	Japan	Site number 3	4
471	Saitama Prefecture	35.9618	138.9787	Japan	Site number 4	4
472	Saitama Prefecture	35.9713	139.5303	Japan	Site number 5	4
473	Lan-Yu Island	22.0254	121.5394	Taiwan	Hungtou	4
474	Lan-Yu island	22.0788	121.5293	Taiwan	Langtao	4
475	Lan-Yu Island	22.0378	121.5665	Taiwan	Yehin	4
476	Penghu Island group	23.6062	119.5136	Taiwan	Hsi-Yu, Yuweng island	4
477	Taitung Prefecture	23.1576	121.3660	Taiwan	Chengkung	3
478	Sarawak	2.5293	112.3288	Malaysia	Selangau Health Center	4
479	Sarawak	2.5325	112.3248	Malaysia	Selangau	4
480	Muang Lampaya	13.8122	100.0248	Thailand	Nakhon Pathom	4
481	Selangor	3.2979	101.7793	Malaysia	Ulu Gombak Forest Reserve	4
482	West Bengal	26.8309	88.2353	India	3 villages in Kurseong subdistrict	3
483	Chieng Mai	18.8038	98.9963	Thailand	Muang	4
484	Chieng Mai	18.8745	99.0489	Thailand	Sansai	4
485	Chieng Mai	18.7390	99.1748	Thailand	Sankumpang	4

486	Chieng Mai	18.9488	98.8857	Thailand	Maerim	4
487	Chieng Mai	18.7206	99.0377	Thailand	Sarapee	4
488	Rajburi and Nakorn-pathom provinces	13.6486	99.7349	Thailand	Unknown	0.5
489	Perak	4.2283	100.5578	Malaysia	Pangkor Island	3
490	Punjab	32.4822	74.8575	Pakistan	Charwa, Sialkot	4
491	Punjab	31.2065	73.8318	Pakistan	Lahore area	2
492	Punjab	30.1834	71.5279	Pakistan	Multan, lower Indus	2
493	Khyber Pakhtnkhwa	34.1443	73.2331	Pakistan	Abbottabad	2
494	Khyber Pakhtunkhwa	33.9130	73.4000	Pakistan	Murree foothills	3
495	Gilgit-Baltistan	34.9070	73.6544	Pakistan	Kaghan valley forest & glacial till	3
496	Gilgit-Baltistan	34.9400	73.7932	Pakistan	Kaghan valley alpine meadow	3
497	Gilgit-Baltistan	35.6531	74.2793	Pakistan	Upper Indus	3
498	Gilgit-Baltistan	36.1974	73.1851	Pakistan	Gilgit Agency mountain desert	3
499	Gilgit-Baltistan	36.1678	74.1775	Pakistan	Nalta, isolated coniferous forest	3
500	Chiba	35.1349	139.8657	Japan	Awa district	3
501	Tokyo	35.5551	139.4428	Japan	Machida district	3
502	Tokyo	35.7408	139.3278	Japan	Fussa district	3
503	Tokyo	35.7290	139.2223	Japan	Itsukaichi district	3
504	Toyama	36.5705	137.1224	Japan	mountain village	1
505	Yamagata	38.1917	140.1001	Japan	Shirataka town	3
506	Yamagata	38.8062	140.2228	Japan	Sakegawa	3
507	Yamagata	38.7575	140.1464	Japan	Tozawa	3
508	Yamagata	38.7553	139.8305	Japan	Tsuruoka	3
509	Yamagata	38.2952	140.2627	Japan	Yamanobe	3
510	Kyoto Prefecture	35.5669	135.1526	Japan	Iwataki	4
511	Niigata Prefecture	37.8200	139.1150	Japan	Kawaguchi	4
512	Kraskino	42.7135	130.7845	Russia	Field	3
513	Taumi	42.5566	130.6947	Russia	Lake side	3
514	Hasan	42.4419	130.6446	Russia	Lake side	3
515	Fatashi	42.8650	130.9196	Russia	River side	3
516	Fadeevka	43.0571	131.1918	Russia	Lake side	3
517	Novo Georgievka	44.0316	131.4012	Russia	River side	3
518	Cherniytino	43.5532	131.8625	Russia	River side	3
519	Karnataka	13.1378	78.1332	India	Kolar, 13 sites in & around	2
520	Chonburi	13.2772	101.4793	Thailand	Bo Thong & Si Racha districts	1
521	Central Luzon	15.1500	120.4500	Philippines	Unknown	1
522	Keelung	25.1457	121.7904	Taiwan	Badouzih Fishing Harbor	4
523	Keelung	25.1389	121.7509	Taiwan	Keelung Harbor	4
524	Taoyuan	25.0797	121.2342	Taiwan	Taoyuan Airport	4
525	Taoyuan	24.9883	121.0145	Taiwan	Yongan Fishing Harbor	4
526	Hsinchu	24.9272	120.9713	Taiwan	Potou Fishing Harbor	4
527	Hsinchu	24.8383	120.9200	Taiwan	Nangliao Fishing Harbor	4
528	Hsinchu	24.8494	120.9188	Taiwan	Hsinchu Fishing Harbor	4
529	Hsinchu	24.7653	120.9034	Taiwan	Haishan Fishing Harbor	4
530	Yunlin	24.9413	121.9015	Taiwan	Dashi Fishing Harbor	4
531	Yunlin	24.5972	121.8576	Taiwan	Suao Harbor	4
532	Taichung	24.2612	120.5228	Taiwan	Taichung Wuchi Harbor	4
533	Yunlin	23.8003	120.1776	Taiwan	Mailiao Harbor	4
534	Tainan	22.9969	120.1556	Taiwan	Anping Fishing Harbor	4

535	Tainan	22.9813	120.2224	Taiwan	East District, Tainan city	4
536	Kaohsiung	22.8678	120.1932	Taiwan	Hsinda Fishing Harbor	4
537	Kaohsiung	22.8150	120.2097	Taiwan	Hsingang Fishing Harbor	4
538	Kaohsiung	22.7622	120.2362	Taiwan	Mito Fishing Harbor	4
539	Kaohsiung	22.7271	120.2547	Taiwan	Uhliao Fishing Harbor	4
540	Kaohsiung	22.6996	120.2727	Taiwan	Zhoying Military Area	4
541	Kaohsiung	22.5971	120.3147	Taiwan	Qianzhen District, Kaohsiung City	4
542	Kaohsiung	22.6483	120.3263	Taiwan	Sanmin District, Kaohsiung City	4
543	Kaohsiung	22.7824	120.3781	Taiwan	Yanchao District, Kaohsiung City	4
544	Kaohsiung	22.5992	120.2888	Taiwan	Kaohsiung International Harbor	4
545	Kaohsiung	22.4833	120.3998	Taiwan	Chungyun Fishing Harbor	4
546	Kaohsiung	22.5746	120.3448	Taiwan	Kaohsiung International Airport	4
547	Pingtung	22.4715	120.4360	Taiwan	Yanpu Fishing Harbor	4
548	Kaohsiung	22.4793	120.3977	Taiwan	Linyuan Fishing Harbor	4
549	Pingtung	22.4647	120.4437	Taiwan	Donggang Fishing Harbor	4
550	Pingtung	22.3730	120.5849	Taiwan	Shueidiliao Fishing Harbor	4
551	Pingtung	22.3631	120.5927	Taiwan	Fangliao Fishing Harbor	4
552	Hualien	24.2946	121.7549	Taiwan	Hoping Fishing Harbor	4
553	Hualien	23.9796	121.6242	Taiwan	Hualien Harbor	4
554	Taitung	22.7917	121.1921	Taiwan	Fugang Fishing Harbor	4
555	Shandong	35.2542	118.3479	China	Linyi	2
556	Shandong	36.2594	117.1021	China	Tai'an	2
557	Shandong	36.1032	117.4163	China	Xintai & Daiyue, Tai'an	2
558	Gifu	35.4146	137.0237	Japan	Kani river	2
559	Gifu	35.4286	137.0024	Japan	Kiso river	2
560	Shandong	35.9237	117.7644	China	Xintai, Daiyue & Yinan	2
561	Shandong	35.8386	118.3190	China	6 districts	1
562	Gifu	35.4730	137.4120	Japan	Ena	2
563	Gifu	36.1484	137.2525	Japan	Takayama	2
564	Okayama	35.0829	133.6920	Japan	Katsuyama	3
565	Okayama	34.9408	133.9614	Japan	Kumenan	3
566	Okayama	34.6900	133.7480	Japan	Souja	3
567	Okayama	34.8676	134.0989	Japan	Wake	3
568	Tokyo	34.0916	139.5248	Japan	Miyake Island, Izu Shichito	4
569	Saga	33.3259	130.2863	Japan	Saga	2
570	Saga	33.3666	130.3535	Japan	Kanzaki	2
571	Saga	33.3579	130.4193	Japan	Kamimine	3
572	Saga	33.4290	130.5123	Japan	Kiyama	3
573	Okayama	34.8814	133.8122	Japan	Kamogawa	3
574	Akita	39.4028	140.4719	Japan	Onakajima	4
575	Akita	39.5092	140.4947	Japan	Nagatoro	4
576	Aomori	40.8677	141.1290	Japan	Noheji	4
577	Aomori	40.7430	141.2650	Japan	Tohoku	4
578	Aomori	40.3890	141.2620	Japan	Sannohe	4
579	Aomori	41.1520	140.8250	Japan	Wakinosawa	4
580	Aomori	40.7101	140.5780	Japan	Namioka	4
581	Aomori	40.6639	140.5040	Japan	Fujisaki	4
582	Aomori	40.6350	140.5500	Japan	Inakadate	4
583	Aomori	40.6110	140.4650	Japan	Hirosaki	4

584	Aomori	40.4808	140.6214	Japan	Ikarigaseki	4
585	Aomori	40.5850	139.9250	Japan	Iwasaki	4
586	Fukui	36.0660	136.5000	Japan	Nomuki, Katsuyama	3
587	Ishikawa	36.5930	136.5830	Japan	Kenmin Park, Kanazawa	4
588	Gunma	36.4102	139.0583	Japan	Miyagi	3
589	Gunma	36.2611	139.3105	Japan	Kasukawa	3
590	Gunma	36.4934	139.0051	Japan	Fujimi	3
591	Gunma	36.4707	138.8480	Japan	Haruna	3
592	Gunma	36.4044	138.9513	Japan	Misato	3
593	Gunma	36.4320	138.7833	Japan	Kurabuchi	3
594	Gunma	36.4055	139.3307	Japan	Kiryu	3
595	Gunma	36.4311	139.2739	Japan	Omama	3
596	Gunma	36.5215	139.2382	Japan	Kurohone	3
597	Gunma	36.4224	139.2780	Japan	Seta Azuma	3
598	Mandalay	21.9660	96.0870	Myanmar	Mandalay	2
599	Manipur	24.4520	94.0260	India	Palel and surroundings	3
600	Bago	18.4970	95.5060	Myanmar	Paungde	4
601	Sagaing	23.2070	94.2980	Myanmar	Kalewa	4
602	Sagaing	24.2260	94.3110	Myanmar	Tamu	4
603	Uttarakhand	29.5920	79.6460	India	Almora	2
604	Manipur	24.9700	93.8800	India	Kanglatongbi	4
605	Manipur	25.0920	94.3600	India	Ukhrul Road	3
606	Manipur	24.8300	93.9400	India	Imphal	2
607	Addu Atoll	-0.6958	73.1560	Maldives	Gan Island	4
608	Uttarakhand	29.3480	79.5510	India	Bhimtal	4
609	Uttarakhand	29.3220	79.5810	India	Naukuchiatal	4
610	Uttarakhand	29.3490	79.5310	India	Sattal	4
611	Gunma	36.4186	139.2377	Japan	Niisato	4
612	Gunma	36.3044	139.1149	Japan	Tamamura	4
613	Gunma	36.2911	139.3755	Japan	Ota	4
614	Gunma	36.3588	139.3156	Japan	Yabuzuka honmachi	4
615	Gunma	36.4895	139.0005	Japan	Shibukawa	4
616	Gunma	36.4475	139.0098	Japan	Yoshioka	4
617	Gunma	36.5500	139.1833	Japan	Akagi	4
618	Gunma	36.5525	138.9222	Japan	Onogami	4
619	Gunma	36.3706	138.6695	Japan	Komochi	4
620	Gunma	36.4775	139.0443	Japan	Kitatachibana	4
621	Kagoshima	31.5860	130.9890	Japan	Ohsumi	4
622	Gunma	36.2585	139.0744	Japan	Fujioka	4
623	Gunma	36.2351	138.9765	Japan	Yoshii	4
624	Gunma	36.2929	138.7984	Japan	Nakazato	4
625	Gunma	36.0831	138.7774	Japan	Ueno	4
626	Gunma	36.2598	138.8896	Japan	Tomioka	4
627	Gunma	36.3008	138.7404	Japan	Myougi	4
628	Gunma	36.1585	138.7114	Japan	Nanmoku	4
629	Gunma	36.2430	138.9217	Japan	Kanra	4
630	Gunma	36.3264	138.8873	Japan	Annaka	4
631	Gunma	36.3131	138.8021	Japan	Matsuida	4
632	Gunma	36.5899	138.8410	Japan	Nakanojo	4

633	Gunma	36.6209	138.9434	Japan	Takayama	4
634	Gunma	36.5314	138.6389	Japan	Agatsuma	4
635	Gunma	36.5709	138.8255	Japan	Agatsuma Azuma	4
636	Gunma	36.6461	139.0442	Japan	Numata	4
637	Gunma	36.7863	139.1626	Japan	Tone	4
638	Gunma	36.7725	139.2252	Japan	Katashina	4
639	Gunma	36.6823	138.9929	Japan	Tsukiyono	4
640	Gunma	36.6950	138.9140	Japan	Niiharu	4
641	Gunma	36.6947	139.1065	Japan	Kawaba	4
642	Gunma	36.6646	139.1287	Japan	Shirasawa	4
643	Gunma	36.2448	139.5420	Japan	Tatebayashi	4
644	West Java	-7.0490	106.5760	Indonesia	Loji	4
645	West Java	-6.7900	106.5760	Indonesia	Cihamerang	4
646	West Sumatra	-0.9800	100.3600	Indonesia	Air Manis	4
647	West Sumatra	-0.2020	100.3410	Indonesia	Koto Rantang	4
648	North Sulawesi	1.4470	124.8130	Indonesia	Malalayang	4
649	North Sulawesi	1.3680	124.8320	Indonesia	Kinilow	4
650	East Kalimantan	-1.2030	116.9650	Indonesia	Manggar Baru	4
651	East Kalimantan	-1.0230	116.8650	Indonesia	Bukit Bangkirai	4
652	Shandong	35.7570	117.9460	China	Mengyin County	3
653	Gifu	35.4920	136.7280	Japan	Unknown	1
654	Kalimantan	-1.1130	116.9150	Indonesia	East Kalimantan (2 sites)	1
655	Gwangju	35.1932	126.8291	South Korea	2 sites	1
656	Banten	-6.2330	106.5321	Indonesia	Duku	2
657	Jakarta	-6.1564	106.8612	Indonesia	Jakarta	1
658	Riau	0.7720	101.7960	Indonesia	Unknown	1
659	Hualien	24.0500	121.5800	Taiwan	Fallow land	1
660	Hualien	24.1000	121.5700	Taiwan	Ploughed land	1
661	Gyeonggyi-do	37.0249	127.2845	South Korea	Anseong	2
662	Gyeonggyi-do	37.7469	127.0329	South Korea	Uijongbu	2
663	Gangwon	37.4161	128.1533	South Korea	Anheung-myeon, Pyeongchang	3
664	Gyeonggyi-do	36.9508	127.0432	South Korea	Songhwa-ri, Pyeongtaek	4
665	Gyeonggyi-do	38.0902	127.2740	South Korea	Uncheon, Pocheon	3
666	Gyeonggyi-do	37.0331	127.5742	South Korea	Neungseo-myeon, Yeoju	3
667	Gangwon	37.9385	127.7465	South Korea	Sinbuk-eup, Chuncheon	3
668	Chungbuk	36.4894	127.7180	South Korea	Boeun-eup	3
669	Chungnam	36.8417	127.1905	South Korea	Anseo-ri, Cheonan	4
670	Kyongbuk	35.9172	128.2267	South Korea	Daega myeon, Seongju-gun	3
671	Kyongnam	35.2980	128.4070	South Korea	Haman	2
672	Chon-buk	35.8649	127.1659	South Korea	Wongeuk, Wangju	3
673	Chungnam	36.7117	126.8496	South Korea	Yesan	3
674	Gyeonggi-do	37.7400	126.4500	South Korea	Gangwha	3
675	Gyeonggi-do	38.1206	127.0813	South Korea	Yeoncheon	2
676	Gyeonggi-do	37.3380	126.8353	South Korea	Ansan	3
677	Chungnam	36.8256	127.0078	South Korea	Asan-si	2
678	Gyeongbuk	36.9387	128.7349	South Korea	Bongwha-gun	2
679	Gyeonggi-do	37.6311	126.9087	South Korea	Ungbong Mt.	3
680	Gyeonggi-do	37.8835	127.6087	South Korea	Kekwan Mt.	3
681	Gyeonggi-do	37.9406	127.6122	South Korea	Gaduk Mt.	3

682	Gyeonggi-do	38.1070	127.3370	South Korea	Mungsung Mt.	3
683	Gangwon	37.9950	127.5030	South Korea	Whaak Mt.	3
684	Gangwon	37.8050	127.5980	South Korea	Jumbong Mt.	3
685	Gangwon	37.8660	127.9790	South Korea	Gali Mt.	3
686	Gangwon	37.7280	128.4650	South Korea	Gyebang Mt.	3
687	Gangwon	37.7320	128.5930	South Korea	Odae Mt.	3
688	Gangwon	38.0530	128.2560	South Korea	Hansok Mt.	3
689	Gangwon	37.8790	128.4580	South Korea	Gachil Mt.	3
690	Gangwon	37.8350	127.6210	South Korea	Samak Mt.	3
691	Chungnam	36.7140	126.6310	South Korea	Kaya Mt.	3
692	Chungnam	36.6730	127.0370	South Korea	Kwangduk Mt.	3
693	Chungnam	36.3570	127.2320	South Korea	Gyeryong Mt.	3
694	Gyeongbuk	36.8130	128.0440	South Korea	Choryong Mt.	3
695	Gyeongnam	35.3370	127.7300	South Korea	Jiri Mt.	3
696	Jeonbuk	35.8160	128.5670	South Korea	Taeduk Mt.	3
697	Jeonbuk	35.9730	127.7760	South Korea	Togyu Mt.	3
698	Chungnam	36.8390	127.1040	South Korea	Cheonan-si	2
699	Seoul	37.5940	126.9800	South Korea	Seoul	1
700	Gyeongnam	35.5220	128.7460	South Korea	Miryang-si	2
701	Gyeongnam	35.0470	128.0680	South Korea	Uiryeong	2
702	Gyeongnam	34.9940	128.3200	South Korea	Goseong	2
703	Gyeongnam	35.5540	127.7290	South Korea	Hamyang	2
704	Gyeonggyi-do	38.0960	127.0190	South Korea	Gunnam-myeon, Yeoncheon	3
705	Gyeonggyi-do	37.9710	126.9200	South Korea	Chuksungmyeon	3
706	Gyeonggyi-do	38.0830	127.2810	South Korea	Wuncheonmyeon	3
707	Jeollabuk-do	35.9800	126.7200	South Korea	Kunsan city	2
708	Gyeongbuk	35.5900	129.3160	South Korea	Ulsan city	2
709	Gyeongnam	35.2380	129.0830	South Korea	Pusan city	2
710	Gangwon	38.2070	128.5860	South Korea	Sokcho city	2
711	Jeollanam-do	34.8170	126.3920	South Korea	Mokpo city	2
712	Jeollanam-do	34.7920	127.6770	South Korea	Yeosu city	2
713	Gyeonggyi-do	38.0100	127.3000	South Korea	US Army camp	2
714	Gyeonggyi-do	38.0300	127.5000	South Korea	ROC Army camp	2
715	Gyeonggyi-do	38.0780	127.5300	South Korea	Sachang-ri	4
716	Gyeonggyi-do	37.6000	127.5000	South Korea	unknown	1
717	Gangwon	38.0500	128.2000	South Korea	unknown	1
718	Jeolla	35.3000	127.0000	South Korea	unknown	1
719	Gyeongsang	35.6000	128.2000	South Korea	unknown	1
720	Chungcheongbuk-do	36.9000	127.7000	South Korea	unknown	1
721	Chungcheongnam-do	36.7000	126.8000	South Korea	unknown	1
722	Gyeongsangbuk-do	36.7800	128.9000	South Korea	unknown	1
723	jeju	33.4000	126.5000	South Korea	unknown	1
724	Gyeonggyi-do	37.8720	127.5220	South Korea	Gapyeong-gun	2
725	Gangwon	38.1120	128.1600	South Korea	Inje-gun	2
726	Gangwon	37.7680	127.8910	South Korea	Hongcheon-gun	2
727	Gangwon	38.1290	127.7150	South Korea	Hwacheon-gun	2
728	Primorye	46.7608	143.3334	Russia	Lake Tunaicha	3
729	Primorye	46.4586	143.4247	Russia	Igrivaya river	3
730	Primorye	46.4679	142.3293	Russia	Uryum river	3

731	Primorye	46.5624	143.0291	Russia	3 sites combined	1
732	Primorye	42.4590	130.6550	Russia	Lake Doretsni	3
733	Primorye	42.4290	130.6330	Russia	Tumyn'tszyan river	3
734	Primorye	42.6830	130.7596	Russia	Yanchikhe river	3
735	Primorye	42.6850	130.6630	Russia	Cherukhe river	3
736	Primorye	42.9360	131.3749	Russia	Brus'ya river	3
737	Primorye	43.5590	131.9010	Russia	Suifun river	3
738	Primorye	43.0224	131.8601	Russia	Russkii island	3
739	Primorye	43.1265	131.9565	Russia	Vladivostok district	2
740	Primorye	42.9337	131.1612	Russia	Khasan district	2
741	Primorye	43.3172	132.6183	Russia	Shkotovsk district	2
742	Primorye	43.2802	133.3041	Russia	Partizansk district	2
743	Primorye	43.1389	133.1247	Russia	Suchansk district	2
744	Primorye	43.4977	131.8417	Russia	Nadezhdinski district	2
745	Primorye	43.9805	133.1408	Russia	Anuchinski district	2
746	Primorye	44.3817	132.1783	Russia	Khorolski district	2
747	Primorye	44.3142	134.2674	Russia	Chuguevski district	2
748	Primorye	43.9725	133.0452	Russia	Ivanovski district	2
749	Primorye	46.6584	135.9187	Russia	Pozharski district	2
750	Primorye	43.1050	131.9280	Russia	Ob'yaseniya river	3
751	Primorye	43.0675	131.9510	Russia	Patrokl bay	3
752	Primorye	43.1880	132.1050	Russia	Shamora bay	3
753	Primorye	42.9650	131.7302	Russia	Popova island	3
754	Primorye	42.8774	131.6590	Russia	Rikorda island	3
755	Primorye	43.2165	132.3772	Russia	Kangauz river	3
756	Primorye	42.9903	132.4065	Russia	Promyslovka town	3
757	Primorye	42.8617	132.4156	Russia	Putyatina island	3
758	Primorye	42.7557	132.3414	Russia	Askol'd island	3
759	Primorye	42.8559	133.0149	Russia	Suchan river, Suchanskii	3
760	Primorye	43.2802	133.3041	Russia	Suchan river, Partizanski	3
761	Primorye	44.5472	132.3785	Russia	Luzanova hill (sopka)	3
762	Primorye	42.7138	130.7840	Russia	Kraskino	3
763	Primorye	43.8183	131.8421	Russia	Slavyanka river	3
764	Primorye	44.4088	131.3809	Russia	Pogranichny	3
765	Sakhalin Oblast	43.8138	146.7495	Russia	Krabozavodsk	3
766	Sakhalin Oblast	43.8676	146.8366	Russia	Bezymyannyi stream	3
767	Sakhalin Oblast	45.0709	147.8348	Russia	Iturup island	3
768	Primorye	42.5059	130.6987	Russia	Lake Tal'mi	3
769	Jiangsu	32.6000	119.9400	Russia	Taizhou	3
770	Chiloe	-41.8900	-73.8300	Chile	Ancud area	4
771	Chiloe	-42.4900	-73.7900	Chile	Castro Area	4
772	Gyeonggi-do	37.0403	126.8697	South Korea	Jangan-myeon, Hwaseong-si	4
773	Chungcheongnam-do	36.7620	126.8678	South Korea	Seonjang-myeon, Asan-si	4
774	Jeollanam-do	35.3036	126.7858	South Korea	Bugil-myeon, Jangseong-gun	4
775	Khon Kaen	16.6500	102.9600	Thailand	Khon Kaen military base	3
776	Chaiyaphum	16.2000	101.9000	Thailand	Chaiyaphum military base	3
777	Buriram	14.3000	102.8000	Thailand	Buriram military base	3
778	Chantaburi	12.6800	102.1500	Thailand	Chantaburi military base	3
779	Rayong	12.8500	101.1000	Thailand	Rayong military base	3

780	Multi	16.2000	102.2000	Thailand	Chaiyaphum & Khon Kaen	0.5
781	Multi	14.6000	101.9000	Thailand	Chaiyaphum, Buriram, Chantaburi, Lopburi, Rayong, Chachoengsao	0.5
782	Multi	15.2000	102.1000	Thailand	Chaiyaphum, Chantaburi, Kohn Kaen, Rayong	0.5
783	Baringo	0.4800	36.0100	Kenya	Perkerra, Marigat	4
784	Uttar Pradesh	26.7990	83.3590	India	Gorakhpur, 12 villages	2
785	Tak	16.4100	98.7493	Thailand	Unknown	1
786	Loei	17.3511	101.1582	Thailand	Unknown	1
787	Sisaket	14.4772	104.4899	Thailand	Unknown	1
788	Chumphon	10.5784	99.2128	Thailand	Unknown	1
789	Phang Nga	8.6643	98.4522	Thailand	Unknown	1
790	Central	7.26	80.6	Sri Lanka	Rajawatta	4
791	Central	7.18	80.52	Sri Lanka	Thambavita	4
792	Central	7.32	80.5	Sri Lanka	Western Slopes	3
793	Southern	6.02	80.25	Sri Lanka	Unawatuna	4

§ 0 = unknown location in country, 0.5 = recognized subregion of a country, 1 to 4 following administrative levels listed in the International Organization for Standardization codes ISO 3166-1 and ISO 3166-2.

Additional file 1: Table S4. Vector genera and species tested for *O. tsutsugamushi*, combining all laboratory tests

Group	Chigger mites & other Acari	Total tested	Total positive	Percentage positive
Trombiculidae	*Actomatacrus* sp.	?	3	-
Ixodida	*Amblyomma auricularium*	4	0	0.0
Ixodida	*Amblyomma ovale*	32	0	0.0
Ixodida	*Amblyomma parvum*	241	0	0.0
Ixodida	*Amblyomma sculptum*	1033	0	0.0
Ixodida	*Amblyomma* sp.	269	0	0.0
Ixodida	*Amblyomma tigrinum*	1	0	0.0
Trombiculidae	*Ascoschoengastia indica*	1273	8	0.6
Trombiculidae	*Ascoschoengastia* sp.	1448	14	1.0
Trombiculidae	*Blankaartia acuscutellaris*	919	66	7.2
Trombiculidae	*Blankaartia* sp.	61	1	1.6
Trombiculidae	*Cheladonta ikaoensis*	42	2	4.8
Laelapidae	*Echinolaelaps echidninus*	?	1	-
Trombiculidae	*Eltonella ichikawa*	6	0	0.0
Trombiculidae	*Euschoengastia koreaensis*	1127	5	0.4
Trombiculidae	*Euschoengastia miyagawai*	?	1	-
Trombiculidae	*Euschoengastia* sp.	?	1	-
Trombiculidae	*Eutrombicula* sp.	9	0	0.0
Trombiculidae	*Eutrombicula wichmanni*	161	14	8.7
Trombiculidae	*Gahrliepia octosetosa*	3	0	0.0
Trombiculidae	*Gahrliepia saduski*	1617	5	0.3
Trombiculidae	*Gahrliepia* sp.	1058	7	0.7
Ixodida	*Haemaphysalis flava*	?	1	-
Ixodida	*Haemaphysalis humerosa*	6	0	0.0
Ixodida	*Haemaphysalis hystricis*	?	1	-
Ixodida	*Haemaphysalis* sp.	?	2	-
Trombiculidae	*Helenicula* sp.	?	2	-
Ixodida	*Ixodes* sp.	12	2	16.7
Laelapidae	*Laelaps turkestanicus*	?	1	-
Trombiculidae	*Leptotrombidium akamushi*	26	28	107.7
Trombiculidae	*Leptotrombidium arenicola*	16	6	37.5
Trombiculidae	*Leptotrombidium arvinum*	898	45	5.0
Trombiculidae	*Leptotrombidium chiangraiensis*	211	49	23.2
Trombiculidae	*Leptotrombidium deliense*	31749	16387	51.6
Trombiculidae	*Leptotrombidium dicum*	1	0	0.0
Trombiculidae	*Leptotrombidium fletcheri*	30	6	20.0
Trombiculidae	*Leptotrombidium fuji*	1595	3	0.2
Trombiculidae	*Leptotrombidium hsui*	1	0	0.0
Trombiculidae	*Leptotrombidium imphalum*	458	69	15.1
Trombiculidae	*Leptotrombidium intermedium*	9486	12	0.1
Trombiculidae	*Leptotrombidium kawamurai*	34	7	20.6
Trombiculidae	*Leptotrombidium kitasatoi*	421	1	0.2

Trombiculidae	*Leptotrombidium laxoscutum*	46	0	0.0
Trombiculidae	*Leptotrombidium linhuaikongense*	87	17	19.5
Trombiculidae	*Leptotrombidium miyajima*	8	0	0.0
Trombiculidae	*Leptotrombidium murotoense*	?	1	-
Trombiculidae	*Leptotrombidium nangii*	?	1	-
Trombiculidae	*Leptotrombidium orientale*	1786	33	1.8
Trombiculidae	*Leptotrombidium pallidum*	16153	1732	10.7
Trombiculidae	*Leptotrombidium palpale*	6385	67	1.0
Trombiculidae	*Leptotrombidium pavlovskyi*	3564	161	4.5
Trombiculidae	*Leptotrombidium peniculatum*	469	7	1.5
Trombiculidae	*Leptotrombidium scutellare*	12403	329	2.7
Trombiculidae	*Leptotrombidium* sp.	2837	122	4.3
Trombiculidae	*Leptotrombidium tachensis*	?	1	-
Trombiculidae	*Leptotrombidium taishanicum*	34	2	5.9
Trombiculidae	*Leptotrombidium waiganmensis*	?	1	-
Trombiculidae	*Leptotrombidium yui*	5	0	0.0
Trombiculidae	*Leptotrombidium zetum*	430	2	0.5
Trombiculidae	*Lorillatum* sp.	159	1	0.6
Trombiculidae	*Microtrombicula chamlongi*	35	7	20.0
Trombiculidae	*Neoschoengastia* sp.	?	1	-
Trombiculidae	*Neotrombicula gardellai*	138	0	0.0
Trombiculidae	*Neotrombicula japonica*	1907	39	2.0
Trombiculidae	*Neotrombicula microti*	4	6	150.0
Trombiculidae	*Neotrombicula mitamurai*	39	7	17.9
Trombiculidae	*Neotrombicula nagayoi*	4	3	75.0
Trombiculidae	*Neotrombicula pomeranzevi*	7	5	71.4
Trombiculidae	*Neotrombicula tamiyai*	9552	0	0.0
Trombiculidae	*Neotromicula shiraii*	?	1	-
Trombiculidae	*Odontacarus majesticus*	185	0	0.0
Trombiculidae	*Odontocarus* sp.	567	21	3.7
Macronyssidae	*Ornithonyssus bacoti*	7	1	14.3
Ixodida	*Rhipicephalus microplus*	1	0	0.0
Ixodida	*Rhipicephalus sanguineus*	1	0	0.0
Trombiculidae	*Schoengastia* sp.	35	3	8.6
Trombiculidae	*Trombicula sadoensis* n. sp.	?	1	-
Trombiculidae	*Trombicula tosa*	?	1	-
Trombiculidae	*Walchia chinensis*	257	19	7.4
Trombiculidae	*Walchia disparunguis pingue*	1	0	0.0
Trombiculidae	*Walchia fragilis*	18	0	0.0
Trombiculidae	*Walchia pacifica*	136	10	7.4
Trombiculidae	*Walchia parapacifica*	10	0	0.0
Trombiculidae	*Walchia* sp.	?	53	-
	Multiple species	328	169	51.5
	Not identified	19284	1029	5.3

Additional file 1: Table S5. Complete list of Tromiculid mite and other Acari reported to be positive for *O. tsutsugamushi*. Location, laboratory test used and reference provided.

SPECIES	COUNTRY	LOCATION	LATITUDE	LONGITUDE	DATE OF COLLECTION	LABORATORY TEST	1st AUTHOR	2nd AUTHOR	TITLE	JOURNAL	YEAR	NOTES
Actomatacrus sp.	China	Fujian	26.640	118.682	1953-54	Xenodiagnosis & serology	Yu, E.S.	Lin, S.H.	Study on the condition of natural infection with R. tsutsugamushi among mites and dometic animals in Fukien	Acta Microbiologica Sinica	1957	
Actomatacrus sp.	China	Zhejiang	29.180	120.089	?	?	Fan, M.Y.	Walker, D.H.	Epidemiology and ecology of rickettsial diseases in the People's Republic of China	Rev. Infect. Dis.	1987	
Ascoschoengastia indica	Thailand	Nonthaburi	13.833	100.517	1992	Direct immunofluorescence	Frances, S.P.	Watcharapichat, P.	Occurrence of Orienta tsutsugamushi in Chiggers (Acari:Trombiculidae) and small animals in an Orchard near Bangkok, Thailand	J. Med. Entomol.	1999	
Ascoschoengastia indica	Malaysia	Selangor	3.298	101.779	1949	Xenodiagnosis	Traub, R	Frick, LP	Observations on the occurence of Rickettsia tsutsugamushi in rats and mites in the Malayan Jungle	Am. J. Hyg.	1950	
Ascoschoengastia indica	China	Fujian	26.640	118.682	1953-54	Xenodiagnosis & serology	Yu, E.S.	Lin, S.H.	Study on the condition of natural infection with R. tsutsugamushi among mites and dometic animals in Fukien	Acta Med. Biol. (Niigata)	1957	
Ascoschoengastia sp.	Thailand	Nonthaburi	13.833	100.517	1992	Direct immunofluorescence	Frances, S.P.	Watcharapichat, P.	Occurrence of Orienta tsutsugamushi in Chiggers (Acari:Trombiculidae) and small animals in an Orchard near Bangkok, Thailand	J. Med. Entomol.	1999	
Ascoschoengastia sp.	Thailand	Phitsanulok	16.751	100.311	1988-90	Direct immunofluorescence	Tanskul, P.	Strickman, D.	Rickettsia tsutsugamushi in chiggers (Acari: Trombiculidae) associated with rodents in central Thailand	J. Med. Entomol.	1994	

Ascoschoengastia sp.	Thailand	Sisaket	14.477	104.490	2015	PCR (56kDa + 47kDa)	Takhampunya, R.	Korkusol, A.	Heterogeneity of Orientia tsutsugamushi genotypes in field-collected trombiculid mites from wild-caught small mammals in Thailand	Plos Negl. Trop. Dis.	2018	
Ascoschoengastia sp.	Thailand	Multiple sites	16.200	102.200	2017-18	PCR (56kDa + 47kDa)	Linsuwananon, P.	Krairojananan, P.	Surveillance for scrub typhus, Rickettsial diseases, and Leptospirosis in US and multinational military training exercise Cobra Gold Sites in Thailand	US Army Med. Dep. J.	2018	
Blankaartia acuscutellaris	Thailand	Phitsanulok	16.751	100.311	1989-90	Direct immunofluorescence	Tanskul, P.	Strickman, D.	Rickettsia tsutsugamushi in chiggers (Acari: Trombiculidae) associated with rodents in central Thailand	J. Med. Entomol.	1994	
Blankaartia acuscutellaris	Thailand	Phitsanulok	16.751	100.311	1993	Xenodiagnosis & serology	Frances, S.P.	Watcharapichat, P.	Investigation of the role of Blankaartia acuscutellaris (Acari: Trombiculidae) as a vector of scrub typhus in central Thailand	J. Med. Entomol.	2001	
Blankaartia acuscutellaris	Thailand	Phitsanulok	16.760	100.271	1989-90	Direct immunofluorescence	Tanskul, P.	Strickman, D.	Rickettsia tsutsugamushi in chiggers (Acari: Trombiculidae) associated with rodents in central Thailand	J. Med. Entomol.	1994	
Blankaartia sp.	Thailand	Phang Nga	8.664	98.452	2015	PCR (56kDa + 47kDa)	Takhampunya, R.	Korkusol, A.	Heterogeneity of Orientia tsutsugamushi genotypes in field-collected trombiculid mites from wild-caught small mammals in Thailand	Plos Negl. Trop. Dis.	2018	
Blankaartia sp.	Thailand	Chumphon	10.578	99.213	2015	PCR (56kDa + 47kDa)	Takhampunya, R.	Korkusol, A.	Heterogeneity of Orientia tsutsugamushi genotypes in field-collected trombiculid mites from wild-caught small mammals in Thailand	Plos Negl. Trop. Dis.	2019	
Cheladonta	Japan	Shizuoka	35.294	138.854	1974	Xenodiagnosis	Asanuma, K.	Kitaoka, M.	Leptotrombidium	J. Hyg.	1974	

ikaoensis									scutellare as a vector of scrub typhus at the endemic area of the foothills of Mt. Fuji, Japan	Epidemiol. Microbiol. Immunol.		
Cheladonta ikaoensis	Japan	Shizuoka	35.361	138.728	1964-66	Complement fixation	Kitaoka, M.	Okubo, K.	Epidemiological survey by means of complement fixation test on scrub typhus in Japan	Acta Med. Biol. (Niigata)	1967	
Echinolaelaps echidninus	China	Zhejiang	29.180	120.089	?	?	Fan, M.Y.	Walker, D.H.	Epidemiology and ecology of rickettsial diseases in the People's Republic of China	Rev. Infect. Dis.	1987	
Euschoengastia koreaensis	South Korea	Jeollanam-do	35.304	126.786	2009	Nested PCR	Choi, Y.J.	Lee, I.Y.	Geographical distribution of Orientia tsutsugamushi strains in chiggers from three provinces of Korea	Microbiol. Immunol.	2018	
Euschoengastia koreaensis	South Korea	Gangwon	38.147	127.313	2005	PCR (56kDa)	Lee, H.I.	Shim, S.K.	Detection of Orientia tsutsugamushi, the causative agent of scrub typhus, in a novel mite species, Euschengastia koreaensis, in Korea	Vector Borne Zoonotic Dis.	2011	
Euschoengastia miyagawai	Japan	Niigata	38.112	138.393	1957-58	Xenodiagnosis & microscopy	Saito, Y.	Otsuru, M.	Notes on Trombiculid mites collected in Sadi Island of Japan and isolation of rickettsia, with a description of Trombicula (Noetrombicula) sadoensis n. sp.	Acta Med. Biol. (Niigata)	1959	
Euschoengastia sp.	China	Zhejiang	29.180	120.089	?	?	Fan, M.Y.	Walker, D.H.	Epidemiology and ecology of rickettsial diseases in the People's Republic of China	Rev. Infect. Dis.	1987	
Eutrombicula wichmanni	Thailand	Nakhon Ratchasima	14.957	102.111	1977-78	Direct immunofluorescence	Shirai, A.	Tanskul, P.L.	Rickettsia tsutsugamushi strains found in chiggers collected in Thailand	Southeast Asian J. Trop. Med. Public Health	1981	Free living
Gahrliepia saduski	Japan	Oita	33.304	131.268	1964-66	Complement fixation	Kitaoka, M.	Okubo, K.	Epidemiological survey by means of complement fixation test on scrub	Acta Med. Biol. (Niigata)	1967	

									typhus in Japan			
Gahrliepia saduski	Japan	Yamagata	38.309	140.149	2012	PCR (56kDa)	Seto, J.	Suzuki, Y	Poposed vector candidate: Leptotrombidium palpale for Shikokoshi type Orientia tsutsugamushi	Microbiol. Immunol.	2013	
Gahrliepia saduski	Japan	Oita	32.233	131.606	1997-99	Nested PCR	Pham, X.D.	Otsuka, Y.	Detection of Orientia tsutsugamushi (Rickettsiales: Rickettsiaceae) in unengorged chiggers (Acari: Ttombiculidae) from Oita Prefecture, Japan, by nested polymerase chain reaction	J. Med. Entomol.	2001	Free living
Gahrliepia sp.	Thailand	Phang Nga	8.664	98.452	2015	PCR (56kDa + 47kDa)	Takhampunya, R.	Korkusol, A.	Heterogeneity of Orientia tsutsugamushi genotypes in field-collected trombiculid mites from wild-caught small mammals in Thailand	Plos Negl. Trop. Dis.	2018	
Gahrliepia sp.	Thailand	Multiple sites	14.600	101.900	2017-18	PCR (56kDa + 47kDa)	Linsuwananon, P.	Krairojananan, P.	Surveillance for scrub typhus, Rickettsial diseases, and Leptospirosis in US and multinational military training exercise Cobra Gold Sites in Thailand	US Army Med. Dep. J.	2018	
Haemaphysalis flava	Japan	Unknown	36.206	138.253	2014	PCR (56kDa)	Namikawa K.	Tanabe A.	Canine Orientia tsutsugamushi infection: report of a case and its epidemicity	Southeast Asian J. Trop. Med. Public Health	2014	
Haemaphysalis hystricis	Japan	Unknown	36.206	138.253	2014	PCR (56kDa)	Namikawa K.	Tanabe A.	Canine Orientia tsutsugamushi infection: report of a case and its epidemicity	Southeast Asian J. Trop. Med. Public Health	2014	
Haemaphysalis sp.	Japan	Unknown	36.206	138.253	2014	PCR (56kDa)	Namikawa K.	Tanabe A.	Canine Orientia tsutsugamushi infection: report of a case and its epidemicity	Southeast Asian J. Trop. Med. Public Health	2014	
Haemaphysalis	India	Manipur	24.452	94.026	1945	Xenodiagnosis	Multiple		Scrub typhus	Scrub Typhus	1947	

sp.									investigations in South East Asia. A report on investigations on scrub typhus by the G.H.O. (India) Field Typhus Research Team, and the Medical Research Council Field Typhus Team, based on the Scrub Typhus Research Laboratory, South East Asia Command	Research Laboratory, South East Asia Command		
Helenicula sp.	Thailand	Khon Kaen	16.650	102.960	2017-18	PCR (56kDa + 47kDa)	Linsuwananon, P.	Krairojananan, P.	Surveillance for scrub typhus, Rickettsial diseases, and Leptospirosis in US and multinational military training exercise Cobra Gold Sites in Thailand	US Army Med. Dep. J.	2018	
Ixodes sp.	China	Shandong	35.924	117.764	2010-12	PCR (56kDa)	Zhang, M.	Zhao, Z.T.	Molecular epidemiology of Orientia tsutsugamushi in chiggers and ticks from domestic rodents in Shandong, northern China	Parasit. Vectors	2013	
Laelaps turkestanicus	China	Zhejiang	29.180	120.089	?	?	Fan, M.Y.	Walker, D.H.	Epidemiology and ecology of rickettsial diseases in the People's Republic of China	Rev. Infect. Dis.	1987	
Leptotrombidium akamushi	Solomon Islands	Ndende (Nendo Island)	-10.799	165.840	1975	Xenodiagnosis & microscopy	Miles, J.A.R.	Austin, F.J.	Scrub typhus in the Eastern Solomon Islands and Northern Vanuatu (New Hebrides)	Am. J. Trop. Med. Hyg.	1981	
Leptotrombidium akamushi	Solomon Islands	Ndende (Nendo Island)	-10.769	165.821	1975	Xenodiagnosis & microscopy	Miles, J.A.R.	Austin, F.J.	Scrub typhus in the Eastern Solomon Islands and Northern Vanuatu (New Hebrides)	Am. J. Trop. Med. Hyg.	1982	
Leptotrombidium akamushi	Solomon Islands	Ndende (Nendo Island)	-10.724	165.799	1975	Xenodiagnosis & microscopy	Miles, J.A.R.	Austin, F.J.	Scrub typhus in the Eastern Solomon Islands and Northern Vanuatu (New Hebrides)	Am. J. Trop. Med. Hyg.	1983	
Leptotrombidium akamushi	Solomon Islands	Ndende (Nendo Island)	-10.723	165.834	1975	Xenodiagnosis & microscopy	Miles, J.A.R.	Austin, F.J.	Scrub typhus in the Eastern Solomon Islands and Northern Vanuatu	Am. J. Trop. Med. Hyg.	1984	

									(New Hebrides)			
Leptotrombidium akamushi	Japan	Niigata	37.745	139.183	1964-66	Complement fixation	Kitaoka, M.	Okubo, K.	Epidemiological survey by means of complement fixation test on scrub typhus in Japan	Acta Med. Biol. (Niigata)	1967	
Leptotrombidium akamushi	Japan	Niigata	37.915	139.147	1966	Xenodiagnosis & microscopy	Saito, Y.		Parasitism of chiggers on the meadow mouse on the islet 'Kawamura nakasu' lying in the lower regions of river Agano, Niigata prefecture, Japan, in 1966, with isolation of Rickettsia from the host and parasites	Acta Med. Biol. (Niigata)	1967	
Leptotrombidium akamushi	Japan	Akita	39.437	140.497	1965	Xenodiagnosis & serology	Kitaoka, M.	Asanuma, K.	Transmission of Rickettsia orientalis to man by Leptotrombidium akamushi at a scrub typhus endemic area in Akita Prefecture, Japan	Am. J. Trop. Med. Hyg.	1974	
Leptotrombidium akamushi	Japan	Akita	39.437	140.497	1964-66	Complement fixation	Kitaoka, M.	Okubo, K.	Epidemiological survey by means of complement fixation test on scrub typhus in Japan	Acta Med. Biol. (Niigata)	1967	
Leptotrombidium akamushi	Japan	Akita	39.437	140.497	1965	Xenodiagnosis & serology	Kitaoka, M.	Asanuma, K.	Monthly observations on rickettsia and complement fixing antibody response in Microtus montebelli placed once on the ground endemic of scrub typhus or inoculated experimentally with Rickettsia orientalis.	J. Hyg. Epidemiol. Microbiol. Immunol.	1968	
Leptotrombidium akamushi	Malaysia	Selangor	2.897	101.729	1969	?	Rapmund, G.	Upham, R.W.	Transovarial development of scrub typhus in a colony of vector mites	Trans. R. Soc. Trop. Med. Hyg.	1969	Free living
Leptotrombidium arenicola	Indonesia	Jakarta	-6.133	106.827	1977	Xenodiagnosis	Dennis, D.T.	Hadi, T.R.	A survey of scrub and murine typhus in the Ancol section of Jakarta, Indonesia	Southeast Asian J. Trop. Med. Public Health	1981	

Leptotrombidium arenicola	Malaysia	Perak	4.228	100.558	1960	Xenodiagnosis	Upham, R.W.	Hubert, A.A.	Distribution of Leptotrombidium (Leptotrombidium) arenicola (Acarina: Trombiculidae) on the ground in West Malaysia	J. Med. Entomol.	1971	
Leptotrombidium arvinum	Thailand	Nakhon Ratchasima	14.957	102.111	1977-78	Direct immunofluorescence	Shirai, A.	Tanskul, P.L.	Rickettsia tsutsugamushi strains found in chiggers collected in Thailand	Southeast Asian J. Trop. Med. Public Health	1981	
Leptotrombidium arvinum	Thailand	Chiang Mai	18.706	98.982	1977-78	Direct immunofluorescence	Shirai, A.	Tanskul, P.L.	Rickettsia tsutsugamushi strains found in chiggers collected in Thailand	Southeast Asian J. Trop. Med. Public Health	1981	
Leptotrombidium chiangraiensis	Thailand	Chiang Rai	20.211	99.930	1994-01	Xenodiagnosis & serology	Lerdthusnee, K.	Khunthirat, B.	Vector competence of Leptotrombidium chiangraiensis chiggers and transmission efficacy and isolation of Orientia tsutsugamushi	J. Med. Entomol.	2003	
Leptotrombidium chiangraiensis	Thailand	Chiang Rai	20.250	99.939	1993-96	Xenodiagnosis & serology	Tanskul, P.	Linthicum, K.J.	A new ecology for scrub typhus associated with a focus of antibiotic resistance in rice farmers in Thailand	J. Med. Entomol.	1998	
Leptotrombidium deliense	Australia	Queensland	-16.383	145.335	1970-71	Xenodiagnosis	Campbell, R.W.	Domrow, R.	Rickettsioses in Australia: isolation of Rickettsia tsutsugamushi and R. australis from naturally infected arthropods	Trans. R. Soc. Trop. Med. Hyg.	1974	
Leptotrombidium deliense	Papua New Guinea	Manus	-2.848	146.232	1945	Xenodiagnosis & microscopy	Philip, C.B.	Kohls, G.M.	Studies on Tsutsugamushi disease (scrub typhus, mite-borne typhus) in New Guinea and adjacent islands. Tsutsugamushi disease with high endemicity on a small South Sea island.	Am. J. Hyg.	1945	
Leptotrombidium deliense	Indonesia	Riau	0.772	101.796	?	Xenodiagnosis	Walch, E.	Keukenschrijver, N.C.	Eenige opmerkingen aangaande de epidemiologie van de pseudotyphus	Nederlands Tijdschrift voor Geneeskunde	1924	

Leptotrombidium deliense	Singapore	Singapore	1.357	103.972	1953	Xenodiagnosis	Lawley, B.J.		The discovery, investigation and control of scrub typhus in Singapore	Trans. R. Soc. Trop. Med. Hyg.	1957	
Leptotrombidium deliense	Malaysia	Selangor	3.080	101.563	1948	Xenodiagnosis	Philip, C.B.	Traub, R.	Chloramphenicol (chloromycetin) in the chemoprophylaxis of scrub typhus (Tsutsugamushi disease). 1. Epidemiological observations on hyperendemic areas of scrub typhus in Malaya	Am. J. Hyg.	1949	
Leptotrombidium deliense	Malaysia	Selangor	3.088	101.615	1948	Xenodiagnosis	Philip, C.B.	Traub, R.	Chloramphenicol (chloromycetin) in the chemoprophylaxis of scrub typhus (Tsutsugamushi disease). 1. Epidemiological observations on hyperendemic areas of scrub typhus in Malaya	Am. J. Hyg.	1949	
Leptotrombidium deliense	Thailand	Rajburi and Nakhon Pathom	13.649	99.735	1963	Xenodiagnosis	Trishnananda, M	Harinasuta, C	Studies on the vector of Rickettsia tsutsugamushi infection in Thailand	Ann. Trop. Med. Parasitol.	1966	
Leptotrombidium deliense	Thailand	Nonthaburi	13.833	100.517	1993-95	Direct immunofluorescence	Frances, S.P.	Watcharapichat, P.	Seasonal Occurrence of Leptotrombidium deliense (Acari: Trombiculidae) attached to sentinel rodents in an orchard near Bangkok, Thailand	J. Med. Entomol.	1999	
Leptotrombidium deliense	Thailand	Nonthaburi	13.833	100.517	1992	Direct immunofluorescence	Frances, S.P.	Watcharapichat, P.	Occurrence of Orienta tsutsugamushi in Chiggers (Acari:Trombiculidae) and small animals in an Orchard near Bangkok, Thailand	J. Med. Entomol.	1999	
Leptotrombidium deliense	Thailand	Phitsanulok	16.751	100.311	1988-89	Direct immunofluorescence	Tanskul, P.	Strickman, D.	Rickettsia tsutsugamushi in chiggers (Acari: Trombiculidae) associated with rodents in central	J. Med. Entomol.	1994	

									Thailand			
Leptotrombidium deliense	Thailand	Phitsanulok	16.760	100.271	1988-89	Direct immunofluorescence	Tanskul, P.	Strickman, D.	Rickettsia tsutsugamushi in chiggers (Acari: Trombiculidae) associated with rodents in central Thailand	J. Med. Entomol.	1994	
Leptotrombidium deliense	China	Guangdong	20.914	110.097	1998-04	PCR (56kDa)	Wang, S.S.	Huang, J.L.	Study on the characterisitics of tsutsugamushi disease in the epidemic areas of south islands in China	Chinese Journal of Epidemiology	2007	
Leptotrombidium deliense	China	Guangdong	20.917	110.600	1998-04	PCR (56kDa)	Wang, S.S.	Huang, J.L.	Study on the characterisitics of tsutsugamushi disease in the epidemic areas of south islands in China	Chinese Journal of Epidemiology	2007	
Leptotrombidium deliense	China	Guangdong	22.037	113.917	1998-04	PCR (56kDa)	Wang, S.S.	Huang, J.L.	Study on the characterisitics of tsutsugamushi disease in the epidemic areas of south islands in China	Chinese Journal of Epidemiology	2007	
Leptotrombidium deliense	Taiwan	Taitung	22.050	121.513	1990	xenodiagnosis/cell culture & indirect IFA & PCR	Tamura, A.	Ohashi, N.	Characterization of Orientia tsutsugamushi isolated in Taiwan by immunofluorescence and restriction fragment length polymorphism analyses	FEMS Microbiol. Lett.	1997	
Leptotrombidium deliense	Taiwan	Lan-Yu island	22.079	121.529	1990	xenodiagnosis/cell culture & indirect IFA & PCR	Tamura, A.	Ohashi, N.	Characterization of Orientia tsutsugamushi isolated in Taiwan by immunofluorescence and restriction fragment length polymorphism analyses	FEMS Microbiol. Lett.	1997	
Leptotrombidium deliense	China	Guangdong	23.257	117.288	1998-99	Xendiagnosis & PCR	Wang, S.	Jiang, P.	Demonstration of the natural focus of Tsutsugamushi disease in the Nan Peng Lie Islands in China	Southeast Asian J. Trop. Med. Public Health	2001	
Leptotrombidium	China	Guangdong	23.257	117.288	1998-04	PCR (56kDa)	Wang, S.S.	Huang, J.L.	Study on the	Chinese	2007	

deliense									characterisitics of tsutsugamushi disease in the epidemic areas of south islands in China	Journal of Epidemiology		
Leptotrombidium deliense	China	Guangdong	23.431	117.093	1998-04	PCR (56kDa)	Wang, S.S.	Huang, J.L.	Study on the characterisitics of tsutsugamushi disease in the epidemic areas of south islands in China	Chinese Journal of Epidemiology	2007	
Leptotrombidium deliense	Taiwan	Fengping	23.585	121.503	1970	Xenodiagnosis & microscopy	Gale, J.L.	Irving, G.S.	Scrub typhus in Eastern Taiwan, 1970	Am. J. Trop. Med. Hyg.	1974	
Leptotrombidium deliense	Myanmar	Sagaing	24.226	94.311	?	Xenodiagnosis	Multiple		Scrub typhus investigations in South East Asia. A report on investigations on scrub typhus by the G.H.O. (India) Field Typhus Research Team, and the Medical Research Council Field Typhus Team, based on the Scrub Typhus Research Laboratory, South East Asia Command	Scrub Typhus Research Laboratory, South East Asia Command	1947	
Leptotrombidium deliense	Taiwan	Fujian	24.440	118.330	1999-00	Nested PCR	Wang, H.C.	Chung, C.L.	Studies on the vectors and pathogens of scrub typhus on murine-like animals in Kinmen Country, Taiwan	Formosan Entomol.	2004	
Leptotrombidium deliense	China	Fujian	24.446	118.083	1992	Xenodiagnosis	Cheng, G.H.	Zhang, C.X.	First isolation of Rickettsia tsutsugamushi from Leptotrombidium deliensis in Xiamen City with scanning electron microscope observation	Journal of Xiamen University Natural Science	1995	
Leptotrombidium deliense	India	Manipur	24.452	94.026	1945	Xenodiagnosis	Multiple		Scrub typhus investigations in South East Asia. A report on investigations on scrub typhus by the G.H.O. (India) Field Typhus Research Team, and the Medical Research Council	Scrub Typhus Research Laboratory, South East Asia Command	1947	

									Field Typhus Team, based on the Scrub Typhus Research Laboratory, South East Asia Command			
Leptotrombidium deliense	Taiwan	Taiwan strait	24.658	119.698	1962	Xenodiagnosis	Cooper, W.C.	Lien, J.C.	Scrub typhus in the Pescadores Islands: An epidemiologic and clinical study	Am. J. trop. Med. Hyg.	1964	
Leptotrombidium deliense	Taiwan	Taiwan strait	24.658	119.698	2006-10	Immunofluorescence + PCR	Kuo, C.C.	Lee, P.L.	Surveillance of potential hosts and vectors of scrub typhus in Taiwan	Parasit. Vectors	2015	
Leptotrombidium deliense	Taiwan	Taiwan strait	24.658	119.698	1975	Xenodiagnosis & microscopy	Olson, J.G	Ho, C.M	Isolation of Rickettsia tsutsugamushi from mammals and chiggers (Fam. Trombiculidae) in the Pescadores Islands, Taiwan.	Trans. R. Soc. Trop. Med. Hyg.	1978	
Leptotrombidium deliense	China	Fujian	25.221	119.475	1997	Nested PCR	Huang, Z.S.	Guo, H.B.	Epidemiologic study of scrub typhus [Orienta tsutsugamushi] on Nanri Island	Chinese Journal of Zoonoses	1998	
Leptotrombidium deliense	Myanmar	Kachin	25.395	97.203	1945	Xenodiagnosis	Mackie, T.T.		Observations on Tsutsugamushi disease (scrub typhus) in Assam and Burma; Preliminary report.	Trans. R. Soc. Trop. Med. Hyg.	1946	
Leptotrombidium deliense	China	Fujian	25.479	119.743	2002	Xendiagnosis & PCR	Cao, M.	Guo, H.	Spring scrub typhus, People's Republic of China	Emerg. Infect. Dis.	2006	
Leptotrombidium deliense	China	Fujian	25.544	118.760	1997-98	Nested PCR	Yan, Y.S.	Zheng, J.	Detection of Orienta tsutsugamushi in chigger mites collected in Fujian coastal and mountain areas in recent years.	Chinese Journal of Zoonoses	1999	
Leptotrombidium deliense	China	Fujian	26.640	118.682	1953-54	Xenodiagnosis & serology	Yu, E.S.	Lin, S.H.	Study on the condition of natural infection with R. tsutsugamushi among mites and dometic animals in Fukien	Acta Microbiologica Sinica	1957	
Leptotrombidium deliense	India	Uttar Pradesh	26.799	83.359	2015	PCR (GroEL)	Sadanandane, C.	Jambulingam, P.	Occurrence of Orientia tsutsugamushi, the Etiological Agent of	Vector Borne Zoonotic Dis.	2018	

									Scrub Typhus in Animal Hosts and Mite Vectors in Areas Reporting Human Cases of Acute Encephalitis Syndrome in the Gorakhpur Region of Uttar Pradesh, India.			
Leptotrombidium deliense	India	Assam	27.328	95.835	1945	Xenodiagnosis	Traub, R.		Observations on Tsutsugamushi disease (scrub typhus) in Assam and Burma. The mite Trombicula deliensis Walch, and its relation to scrub typhus in Assam	Am. J. Hyg.	1949	
Leptotrombidium deliense	India	Assam	27.328	95.835	1945	Xenodiagnosis	Mackie, T.T.		Observations on Tsutsugamushi disease (scrub typhus) in Assam and Burma; Preliminary report.	Trans. R. Soc. Trop. Med. Hyg.	1946	
Leptotrombidium deliense	China	Tibet	29.321	95.325	1973	Xenodiagnosis	Fan, R.S.	Zhang, Y.	Baseline survey on taxonomy and population fluctuation of murine animals and their ectoparasites: forcast research on the related diseases of natural focus in Maoming Port.	Chinese Journal of Vector Biology and Control	2003	
Leptotrombidium deliense	India	Uttarakhand	29.592	79.646	?	Xenodiagnosis	Multiple		Scrub typhus investigations in South East Asia. A report on investigations on scrub typhus by the G.H.O. (India) Field Typhus Research Team, and the Medical Research Council Field Typhus Team, based on the Scrub Typhus Research Laboratory, South East Asia Command	Scrub Typhus Research Laboratory, South East Asia Command	1947	
Leptotrombidium deliense	Pakistan	Punjab	32.482	74.858	1962-65	Xenodiagnosis	Traub, R.	Wisseman, C.L.	The occurrence of scrub typhus in unusual habitats in West Pakistan	Trans. R. Soc. Trop. Med. Hyg.	1967	

Leptotrombidium deliense	China	Unknown	35.862	104.195	1957	Serology (OXK)	Ch'iu, F-H.	Chung, H-L.	Isolation of Rickettsia tsutsugamushi from patients, rats and mites	Chinese Med. J.	1962	
Leptotrombidium deliense	Malaysia	Johor	1.668	103.785	?	Direct immunofluorescence	Shirai, A.	Dohany, A.L.	Serological classification of Rickettsia tsutsugamushi organisms found in chiggers (Acarina: Trombiculidae) collected in Peninsular Malaysia	Trans. R. Soc. Trop. Med. Hyg.	1981	Free living
Leptotrombidium deliense	Malaysia	Pahang	3.171	102.319	1975-77	Direct immunofluorescence	Dohany, A.L.	Shirai, A.	Variation in populations of chigger vectors of scrub typhus in developing oil palm areas of different ages	Jap. J. Med. Sci. Biol.	1980	Free living
Leptotrombidium deliense	Malaysia	Selangor	3.183	101.600	1973-74	Direct immunofluorescence	Roberts, L.W.	Muul, I.	Numbers of Leptotrombidium (Leptotrombidium) deliense (Acarina: Trombiculidae) and prevalence of Rickettsia tsutsugamushi in adjacent habitats of peninsular Malaysia	Southeast Asian J. Trop. Med. Public Health	1977	Free living
Leptotrombidium deliense	Malaysia	Selangor	3.211	101.505	1976-77	Xenodiagnosis & direct IF	Shirai, A.	Robinson, D.M.	Rickettsia tsutsugamushi infections in chiggers and small mammals on a mature oil palm estate	Southeast Asian J. Trop. Med. Public Health	1978	Free living
Leptotrombidium deliense	Thailand	Rajburi and Nakhon Pathom	13.649	99.735	1963	Xenodiagnosis	Trishnananda, M	Harinasuta, C	Studies on the vector of Rickettsia tsutsugamushi infection in Thailand	Ann. Trop. Med. Parasitol.	1966	Free living
Leptotrombidium deliense	Thailand	Nakhon Ratchasima	14.957	102.111	1977-78	Direct immunofluorescence	Shirai, A.	Tanskul, P.L.	Rickettsia tsutsugamushi strains found in chiggers collected in Thailand	Southeast Asian J. Trop. Med. Public Health	1981	Free living
Leptotrombidium deliense	Thailand	Ubon Ratchathani	15.183	105.113	1977-78	Indirect immunofluorescence	Shirai, A.	Tanskul, P.L.	Rickettsia tsutsugamushi strains found in chiggers collected in Thailand	Southeast Asian J. Trop. Med. Public Health	1981	Free living
Leptotrombidium deliense	China	Hainan	16.330	112.026	2000	PCR (56kDa)	Wang, S.S.	Zhan, D.C.	Sequence analysis of Orientia tsutsugamushi	Southeast Asian J. Trop.	2002	Free living

									DNA from mites collected in Xisa archipelago, China	Med. Public Health		
Leptotrombidium deliense	Thailand	Chiang Mai	18.706	98.982	1977-78	Direct immunofluorescence	Shirai, A.	Tanskul, P.L.	Rickettsia tsutsugamushi strains found in chiggers collected in Thailand	Southeast Asian J. Trop. Med. Public Health	1981	Free living
Leptotrombidium deliense	Taiwan	Taiwan strait	24.658	119.698	1962	Xenodiagnosis	Cooper, W.C.	Lien, J.C.	Scrub typhus in the Pescadores Islands: An epidemiologic and clinical study	Am. J. trop. Med. Hyg.	1964	Free living
Leptotrombidium deliense	India	Assam	27.328	95.835	?	Xenodiagnosis	Davis, G.E.	Austrian, R.C.	Observations on Tsutsugamushi disease (scrub typhus) in Assam and Burma	Am. J. Hyg.	1947	Free living
Leptotrombidium fletcheri	Papua New Guinea	Oro	-9.131	148.692	1945	Xenodiagnosis	Manwaring, W. H.		Vectors of Tsutsugamushi disease (scrub typhus)	Cal. West. Med.	1945	
Leptotrombidium fletcheri	Papua New Guinea	Oro	-8.762	148.367	1943	Xenodiagnosis & serology	Blake, F.G.	Maxcy, K.F.	Studies on Tsutsugamushi disease (scrub typhus, mite-borne typhus) in New Guinea and adjacent islands: epidemiology, clinical observations, and etiology in the Dobadura area	Am. J. Hyg.	1945	
Leptotrombidium fletcheri	Malaysia	Johor	1.668	103.785	?	Direct immunofluorescence	Shirai, A.	Dohany, A.L.	Serological classification of Rickettsia tsutsugamushi organisms found in chiggers (Acarina: Trombiculidae) collected in Peninsular Malaysia	Trans. R. Soc. Trop. Med. Hyg.	1981	Free living
Leptotrombidium fletcheri	Malaysia	Selangor	3.264	101.588	?	Direct immunofluorescence	Shirai, A.	Dohany, A.L.	Serological classification of Rickettsia tsutsugamushi organisms found in chiggers (Acarina: Trombiculidae) collected in Peninsular Malaysia	Trans. R. Soc. Trop. Med. Hyg.	1981	Free living
Leptotrombidium fletcheri	Malaysia	Selangor	3.211	101.505	1976-77	Xenodiagnosis & serology	Shirai, A.	Robinson, D.M.	Rickettsia tsutsugamushi infections in chiggers and	Southeast Asian J. Trop.	1978	Free living

									small mammals on a mature oil palm estate	Med. Public Health		
Leptotrombidium fuji	Japan	Aichi	35.228	137.303	1998	Cell culture & 56 kDa PCR	Tamura, A.	Makisaka, Y.	Isolation of Orientia tsutsugamushi from Leptotrombidium fuji and its characterization	Microbiol. Immunol.	2000	
Leptotrombidium fuji	Japan	Yamagata	38.309	140.149	2012	PCR (56kDa)	Seto, J.	Suzuki, Y	Poposed vector candidate: Leptotrombidium palpale for Shikokoshi type Orientia tsutsugamushi	Microbiol. Immunol.	2013	
Leptotrombidium fuji	Japan	Oita	32.233	131.606	1997-99	Nested PCR	Pham, X.D.	Otsuka, Y.	Detection of Orientia tsutsugamushi (Rickettsiales: Rickettsiaceae) in unengorged chiggers (Acari: Ttombiculidae) from Oita Prefecture, Japan, by nested polymerase chain reaction	J. Med. Entomol.	2001	Free living
Leptotrombidium fuji	Japan	Oita	33.199	131.517	1997-99	Nested PCR	Pham, X.D.	Otsuka, Y.	Detection of Orientia tsutsugamushi (Rickettsiales: Rickettsiaceae) in unengorged chiggers (Acari: Ttombiculidae) from Oita Prefecture, Japan, by nested polymerase chain reaction	J. Med. Entomol.	2001	Free living
Leptotrombidium imphalum	Thailand	Chiang Rai	20.250	99.939	1993-96	Xenodiagnosis & direct IF	Tanskul, P.	Linthicum, K.J.	A new ecology for scrub typhus associated with a focus of antibiotic resistance in rice farmers in Thailand	J. Med. Entomol.	1998	
Leptotrombidium intermedium	China	Shandong	35.924	117.764	2010-12	PCR (56kDa)	Zhang, M.	Zhao, Z.T.	Molecular epidemiology of Orientia tsutsugamushi in chiggers and ticks from domestic rodents in Shandong, northern China	Parasit. Vectors	2013	
Leptotrombidium intermedium	Japan	Niigata	38.112	138.393	1957-58	Xenodiagnosis & microscopy	Saito, Y.	Otsuru, M.	Notes on Trombiculid mites collected in Sadi Island of Japan and	Acta Med. Biol. (Niigata)	1959	

									isolation of rickettsia, with a description of Trombicula (Noetrombicula) sadoensis n. sp.			
Leptotrombidium intermedium	Japan	Yamagata	38.309	140.149	2012	PCR (56kDa) & indirect IF	Seto, J.	Suzuki, Y	Poposed vector candidate: Leptotrombidium palpale for Shikokoshi type Orientia tsutsugamushi	Microbiol. Immunol.	2013	
Leptotrombidium intermedium	Japan	Kyoto	35.550	135.130	1996-99	Indirect immunofluorescence	Takahashi, M.	Misumi, H.	Mite Vectors (Acari: Trombiculidae) of Scrub Typhus in a New Endemic Area in Northern Kyoto, Japan	J. Med. Entomol.	2004	Free living
Leptotrombidium intermedium	Japan	Kyoto	35.550	135.130	1997-01	xenodiagnosis/cell culture & indirect IFA	Misumi, H.	Takahashi, M.	Distribution of infective spots composed of unfed larvae infected with Orientia tsutsugamushi in Leptotrombidium mites and their annual fluctuations on the soil surface in an endemic area of tsutsugamushi disease (Acari: Trombiculidae)	Med. Entomol. Zool.	2002	Free living
Leptotrombidium intermedium	Japan	Kyoto	35.567	135.153	1997-98	Indirect immunofluorescence	Urakami, H	Takahashi, M	Detection, isolation and characterization of Orientia tsutsugamushi in Leptotrombidium intermedium	Med. Entomol. Zool.	2000	Free living
Leptotrombidium intermedium	Japan	Niigata	37.820	139.115	1996	Xenodiagnosis & indirect IF	Urakami, H	Takahashi, M	Detection, isolation and characterization of Orientia tsutsugamushi in Leptotrombidium intermedium	Med. Entomol. Zool.	2000	Free living
Leptotrombidium kawamurai	Japan	Hokkaido	43.058	141.476	1964-66	Complement fixation	Kitaoka, M.	Okubo, K.	Epidemiological survey by means of complement fixation test on scrub typhus in Japan	Acta Med. Biol. (Niigata)	1967	
Leptotrombidium kawamurai	Japan	Hokkaido	43.058	141.476	1959-68	Xenodiagnosis & complement fixation	Kitaoka, M.	Asanuma, K,	Seasonal occurrence of trombiculid mites species	J. Hyg. Epidemiol.	1973	

									and Leptotrombidium kawamurai (Acarina, Trombiculidae) as a carrier of Rickettsia orientalis in the Nopporo area, Hokkaido, Japan.	Microbiol. Immunol.		
Leptotrombidium kitasatoi	Japan	Yamagata	38.309	140.149	2012	PCR (56kDa) & indirect IF	Seto, J.	Suzuki, Y	Poposed vector candidate: Leptotrombidium palpale for Shikokoshi type Orientia tsutsugamushi	Microbiol. Immunol.	2013	
Leptotrombidium kitasatoi	Japan	Oita	32.233	131.606	1997-99	Nested PCR	Pham, X.D.	Otsuka, Y.	Detection of Orientia tsutsugamushi (Rickettsiales: Rickettsiaceae) in unengorged chiggers (Acari: Ttombiculidae) from Oita Prefecture, Japan, by nested polymerase chain reaction	J. Med. Entomol.	2001	Free living
Leptotrombidium linhuaikongense	China	Anhui	32.777	117.989	2009-12	Xenodiagnosis	Cao, M.	Che, L.	Determination of scrub typhus suggests a new epidemic focus in Anhui Province, China	Sci. Rep.	2016	
Leptotrombidium linhuaikongense	China	Shandong	35.266	117.977	1995-03	Xendiagnosis & PCR	Liu, Y.	Jia, N.	Consistency of the key genotypes of Orientia tsutsugamushi in scrub typhus patients, rodents, and chiggers from a New Endemic focus of Northern China	Cell Biochem. Biophys.	2013	
Leptotrombidium linhuaikongense	China	Shandong	35.266	117.977	1995-03	PCR (56kDa)	Liu, Y.	Jia, N.	Consistency of the key genotypes of Orientia tsutsugamushi in scrub typhus patients, rodents, and chiggers from a New Endemic focus of Northern China	Cell Biochem. Biophys.	2013	
Leptotrombidium linhuaikongense	China	Shandong	35.266	117.977	1995-99	Xenodiagnosis & indirect IF	Liu, Y.	Yang, Z.	First isolation of Orientia (O.) tsutsugamushi from larvae and reared nymphs of Leptotrombidium (L.)	Syst. Appl. Acarol.	2002	

									linhuaikongense collected from wild rodents in Fei County, Shandong Province, China			
Leptotrombidium linhuaikongense	China	Shandong	35.286	118.185	1995-96	Xenodiagnosis & indirect IF	Liu, Y.	Yang, Z.	Isolation of Rickettsia tsutsugamushi from Trombiculid mites (Acari: Trombiculidae) in Feixian county, Shandong province, China	Entomologia Sinica	1999	
Leptotrombidium linhuaikongense	China	Shandong	35.362	118.090	1995-96	Xenodiagnosis & indirect IF	Liu, Y.	Yang, Z.	Epidemiological study of autumn-winter type scrub typhus in a new endemic focus of Fei County, Shandong Province, China	Syst. Appl. Acarol.	2000	
Leptotrombidium linhuaikongense	China	Shandong	35.879	117.928	1995-02	Xendiagnosis & PCR	Liu, Y.	Zhao, Z.	Characterization of Orientia tsutsugamushi strains isolated in Shandong Province, China by immunofluorescence and restriction fragment length polymorphism (RFLP) analyses	Southeast Asian J. Trop. Med. Public Health	2004	
Leptotrombidium linhuaikongense	China	Shandong	35.924	117.764	2010-12	PCR (56kDa)	Zhang, M.	Zhao, Z.T.	Molecular epidemiology of Orientia tsutsugamushi in chiggers and ticks from domestic rodents in Shandong, northern China	Parasit. Vectors	2013	
Leptotrombidium murotoense	Japan	Oita	33.304	131.268	1964-66	Complement fixation	Kitaoka, M.	Okubo, K.	Epidemiological survey by means of complement fixation test on scrub typhus in Japan	Acta Med. Biol. (Niigata)	1967	
Leptotrombidium nangii	China	Zhejiang	29.180	120.089	?	?	Fan, M.Y.	Walker, D.H.	Epidemiology and ecology of rickettsial diseases in the People's Republic of China	Rev. Infect. Dis.	1987	
Leptotrombidium orientale	South Korea	Jeollanam-do	34.673	126.925	1992-93	Immunofluorescence + PCR	Ree, H. I	Chang, W. H	Detection of Orientia tsutsugamushi DNA in individual trombiculids using polymerase chain reaction in Korea	Med. Entomol. Zool.	1997	

Leptotrombidium orientale	South Korea	Gwangju	35.193	126.829	2014-15	Immunofluorescence + PCR	Park, J. W	Chung, J.K	Seroepidemiological Survey of Zoonotic Diseases in Small Mammals with PCR Detection of Orientia tsutsugamushi in Chiggers, Gwangju, Korea	Korean J. Parasitol.	2016	
Leptotrombidium orientale	South Korea	Gwangju	35.193	126.829	2014-16	PCR (56kDa)	Park, J.W.	Kim, S.H.	Molecular epidemiology of an Orientia tsutsugamushi gene encoding 56-kDa type-specific antigen in chiggers, small mammals and patients from Southwest region of Korea	Am. J. Trop. Med. Hyg.	2018	
Leptotrombidium orientale	South Korea	Jeollanam-do	35.218	126.323	1995	Immunofluorescence + PCR	Ree, H. I	Chang, W. H	Detection of Orientia tsutsugamushi DNA in individual trombiculids using polymerase chain reaction in Korea	Med. Entomol. Zool.	1997	
Leptotrombidium orientale	South Korea	Jeollanam-do	35.304	126.786	2009	Nested PCR	Choi, Y.J.	Lee, I.Y.	Geographical distribution of Orientia tsutsugamushi strains in chiggers from three provinces of Korea	Microbiol. Immunol.	2018	
Leptotrombidium orientale	South Korea	Gyeongsangnam-do	35.567	128.166	2005	PCR (56kDa)	Lee, H.I.	Shim, S.K.	Detection of Orientia tsutsugamushi, the causative agent of scrub typhus, in a novel mite species, Euschengastia koreaensis, in Korea	Vector Borne Zoonotic Dis.	2011	
Leptotrombidium orientale	South Korea	Jeollabuk-do	35.960	126.995	1992-93	Immunofluorescence + PCR	Ree, H. I	Chang, W. H	Detection of Orientia tsutsugamushi DNA in individual trombiculids using polymerase chain reaction in Korea	Med. Entomol. Zool.	1997	
Leptotrombidium orientale	South Korea	Chungcheongnam-do	36.762	126.868	2009	Nested PCR	Choi, Y.J.	Lee, I.Y.	Geographical distribution of Orientia tsutsugamushi strains in chiggers from three provinces of Korea	Microbiol. Immunol.	2018	
Leptotrombidium orientale	South Korea	Chungcheongbuk-do	36.991	127.926	2005	PCR (56kDa)	Lee, H.I.	Shim, S.K.	Detection of Orientia tsutsugamushi, the	Vector Borne Zoonotic Dis.	2011	

									causative agent of scrub typhus, in a novel mite species, Euschengastia koreaensis, in Korea			
Leptotrombidium orientale	South Korea	Gangwon-do	37.449	129.165	1992-93	Immunofluorescence + PCR	Ree, H. I	Chang, W. H	Detection of Orientia tsutsugamushi DNA in individual trombiculids using polymerase chain reaction in Korea	Med. Entomol. Zool.	1997	
Leptotrombidium orientale	Russia	Primorsky Krai	42.430	130.678	1963-65	Xenodiagnosis & Indirect IF	Kulagin, S.M.	Tarasevich, I.V.	On the natural focus of scrub typhus in the south of the Primorie area of the USSR	Acta Med. Biol. (Niigata)	1967	
Leptotrombidium orientale	Russia	Primorsky Krai	42.459	130.655	1964-65	Direct immunofluorescence	Kudryashova, N.I.	Mirolyubova, L.N.	Natural infection of Trombiculid mites with the rickettsiae of tsutsugamushi disease in the Maritime Province	Meditsinskaya Parazitologiya i Parazitarnye Bolezni	1968	
Leptotrombidium pallidum	Japan	Oita	33.304	131.268	1964-66	Complement fixation	Kitaoka, M.	Okubo, K.	Epidemiological survey by means of complement fixation test on scrub typhus in Japan	Acta Med. Biol. (Niigata)	1967	
Leptotrombidium pallidum	Japan	Shizuoka	34.651	138.859	1964-66	Complement fixation	Kitaoka, M.	Okubo, K.	Epidemiological survey by means of complement fixation test on scrub typhus in Japan	Acta Med. Biol. (Niigata)	1967	
Leptotrombidium pallidum	Japan	Shizuoka	34.651	138.859	1952-60	Xenodiagnosis	Asanuma, K.	Okubo, K.	Determination of the vector mites of scrub typhus in Japan	Jap. J. Med. Sci. Biol.	1962	
Leptotrombidium pallidum	South Korea	Jeollanam-do	34.673	126.925	1992-93	Immunofluorescence + PCR	Ree, H. I	Chang, W. H	Detection of Orientia tsutsugamushi DNA in individual trombiculids using polymerase chain reaction in Korea	Med. Entomol. Zool.	1997	
Leptotrombidium pallidum	South Korea	Jeollanam-do	34.997	127.044	1995	Immunofluorescence + PCR	Ree, H. I	Chang, W. H	Detection of Orientia tsutsugamushi DNA in individual trombiculids using polymerase chain reaction in Korea	Med. Entomol. Zool.	1997	
Leptotrombidium pallidum	Japan	Kanagawa	35.144	139.621	1964-66	Complement fixation	Kitaoka, M.	Okubo, K.	Epidemiological survey by means of complement	Acta Med. Biol. (Niigata)	1967	

									fixation test on scrub typhus in Japan			
Leptotrombidium pallidum	Japan	Kanagawa	35.144	139.621	1952-60	Xenodiagnosis	Asanuma, K.	Okubo, K.	Determination of the vector mites of scrub typhus in Japan	Jap. J. Med. Sci. Biol.	1962	
Leptotrombidium pallidum	South Korea	Jeollanam-do	35.173	126.859	1992-93	Immunofluorescence + PCR	Ree, H. I	Chang, W. H	Detection of Orientia tsutsugamushi DNA in individual trombiculids using polymerase chain reaction in Korea	Med. Entomol. Zool.	1997	
Leptotrombidium pallidum	South Korea	Jeollanam-do	35.200	126.502	1995	Immunofluorescence + PCR	Ree, H. I	Chang, W. H	Detection of Orientia tsutsugamushi DNA in individual trombiculids using polymerase chain reaction in Korea	Med. Entomol. Zool.	1997	
Leptotrombidium pallidum	Japan	Aichi	35.225	137.329	1989-90	xenodiagnosis/cell culture & 56 kDa PCR	Tamura, A.	Makisaka, Y.	Isolation of Orientia tsutsugamushi from Leptotrombidium fuji and its characterization	Microbiol. Immunol.	2000	
Leptotrombidium pallidum	South Korea	Jeollanam-do	35.264	126.476	1995	Immunofluorescence + PCR	Ree, H. I	Chang, W. H	Detection of Orientia tsutsugamushi DNA in individual trombiculids using polymerase chain reaction in Korea	Med. Entomol. Zool.	1997	
Leptotrombidium pallidum	South Korea	Jeollanam-do	35.304	126.786	2009	Nested PCR	Choi, Y.J.	Lee, I.Y.	Geographical distribution of Orientia tsutsugamushi strains in chiggers from three provinces of Korea	Microbiol. Immunol.	2018	
Leptotrombidium pallidum	Japan	Gifu	35.429	137.002	1984	Xenodiagnosis & indirect IF	Kasuya, S.	Hioki, A.	Studies on Tsutsugamushi disease in Gifu Prefecture. 2. A speculation on the vector	Kansenshogaku Zasshi	1985	
Leptotrombidium pallidum	Japan	Gifu	35.439	137.045	1984-86	Xenodiagnosis & indirect IF	Iwasa, M.	Kasuya, S.	Trombiculid Mites (Acari: Trombiculidae) and Rickettsia tsutsugamushi Isolated from Wild Rodents in a New Endemic Area of Japan	J. Med. Entomol.	1990	
Leptotrombidium pallidum	Japan	Gifu	35.492	136.728	1994	Xenodiagnosis & indirect IF	Yamashita, T.	Kasuya, S.	Transmission of Rickettsia tsutsugamushi strains among humans, wild	J. Clin. Microbiol.	1994	

									rodents, and trombiculid mites in an area of Japan in which Tsutsugamushi disease is newly endemic			
Leptotrombidium pallidum	Japan	Kyoto	35.550	135.130	1996-99	xenodiagnosis/cell culture & indirect IFA	Takahashi, M.	Misumi, H.	Mite Vectors (Acari: Trombiculidae) of Scrub Typhus in a New Endemic Area in Northern Kyoto, Japan	J. Med. Entomol.	2004	
Leptotrombidium pallidum	Japan	Kyoto	35.550	135.130	1996-99	Indirect immunofluorescence	Takahashi, M.	Misumi, H.	Mite Vectors (Acari: Trombiculidae) of Scrub Typhus in a New Endemic Area in Northern Kyoto, Japan	J. Med. Entomol.	2004	
Leptotrombidium pallidum	Japan	Kyoto	35.550	135.130	1997-01	xenodiagnosis/cell culture & indirect IFA	Misumi, H.	Takahashi, M.	Distribution of infective spots composed of unfed larvae infected with Orientia tsutsugamushi in Leptotrombidium mites and their annual fluctuations on the soil surface in an endemic area of tsutsugamushi disease (Acari: Trombiculidae)	Med. Entomol. Zool.	2002	
Leptotrombidium pallidum	Japan	Saitama	35.985	139.090	1985-86	Xenodiagnosis & indirect IF	Takahashi, M.	Murata, M.	Aggregated distribution of infective spots composed of Leptotrombidium pallidum, highly prevalent with Rickettsia tsutsugamushi, demonstrated by sentinel voles, Microtus montebelli, on the ground	Jpn. J. Exp. Med.	1990	
Leptotrombidium pallidum	Japan	Saitama	35.985	139.090	1985	Direct immunofluorescence	Takahashi, M.	Murata, M.	Vector mite of Tsutsugamushi disease in the area of Hitsujiyama, Chichibu City, Saitama Prefecture: accumulation of Rickettsia tsutsugamushi in the endemic spot	Journal of Saitama Medical School	1992	

Leptotrombidium pallidum	Japan	Saitama	35.985	139.090	1985	Xenodiagnosis & indirect IF	Takahashi, M.	Murata, M.	Vector mite of Tsutsugamushi disease in the area of Hitsujiyama, Chichibu City, Saitama Prefecture: accumulation of Rickettsia tsutsugamushi in the endemic spot	Journal of Saitama Medical School	1992	
Leptotrombidium pallidum	Japan	Saitama	35.985	139.090	1984-85	Xenodiagnosis & indirect IF	Takahashi, M.	Murata, M.	Trombiculid mites and Rickettsia tsutsugamushi isolated from wild rodents and mites in an endemic area of Saitama Prefecture, Japan	Japanese Journal of Sanitary Zoology	1993	
Leptotrombidium pallidum	South Korea	Chungcheongnam-do	36.762	126.868	2009	Nested PCR	Choi, Y.J.	Lee, I.Y.	Geographical distribution of Orientia tsutsugamushi strains in chiggers from three provinces of Korea	Microbiol. Immunol.	2018	
Leptotrombidium pallidum	Japan	Toyama	36.818	137.598	1979-84	Xenodiagnosis & indirect IF	Ishikura, M.	Watanabe, M.	Epidemiological Studies on the Background of the Endemic Occurrence of Tsutsugamushi Disease in Toyama Prefecture. I. Epidemiology of Infection with Rickettsia tsutsugamuchi among Field Rodents in Endemic and Nonendemic Areas	Microbiol. Immunol.	1985	
Leptotrombidium pallidum	South Korea	Gyeonggi-do	37.040	126.870	2009	Nested PCR	Choi, Y.J.	Lee, I.Y.	Geographical distribution of Orientia tsutsugamushi strains in chiggers from three provinces of Korea	Microbiol. Immunol.	2018	
Leptotrombidium pallidum	South Korea	Gyeonggi-do	37.234	127.707	2005	PCR (56kDa)	Lee, H.I.	Shim, S.K.	Detection of Orientia tsutsugamushi, the causative agent of scrub typhus, in a novel mite species, Euschengastia koreaensis, in Korea	Vector Borne Zoonotic Dis.	2011	
Leptotrombidium pallidum	South Korea	Gyeonggi-do	37.298	127.637	2005	PCR (56kDa)	Lee, H.I.	Shim, S.K.	Detection of Orientia tsutsugamushi, the causative agent of scrub typhus, in a novel mite	Vector Borne Zoonotic Dis.	2011	

									species, Euschengastia koreaensis, in Korea			
Leptotrombidium pallidum	Japan	Niigata	37.446	138.851	1952-60	Xenodiagnosis	Asanuma, K.	Okubo, K.	Determination of the vector mites of scrub typhus in Japan	Jap. J. Med. Sci. Biol.	1962	
Leptotrombidium pallidum	South Korea	Gangwon-do	37.536	129.115	1992-93	Immunofluorescence + PCR	Ree, H. I	Chang, W. H	Detection of Orientia tsutsugamushi DNA in individual trombiculids using polymerase chain reaction in Korea	Med. Entomol. Zool.	1997	
Leptotrombidium pallidum	South Korea	Gyeonggi-do	37.681	126.839	1989	Indirect immunofluorescence	Ree, H.I	Lee, I. Y	Determination of the vector species of tsutsugamushi disease in Korea	Korean J. Parasitol.	1991	
Leptotrombidium pallidum	South Korea	Gyeonggi-do	37.683	126.840	1992-93	Immunofluorescence + PCR	Ree, H. I	Chang, W. H	Detection of Orientia tsutsugamushi DNA in individual trombiculids using polymerase chain reaction in Korea	Med. Entomol. Zool.	1997	
Leptotrombidium pallidum	Japan	Niigata	37.745	139.183	1964-66	Complement fixation	Kitaoka, M.	Okubo, K.	Epidemiological survey by means of complement fixation test on scrub typhus in Japan	Acta Med. Biol. (Niigata)	1967	
Leptotrombidium pallidum	Japan	Niigata	37.915	139.147	1966	Xenodiagnosis & microscopy	Saito, Y.		Parasitism of chiggers on the meadow mouse on the islet 'Kawamura nakasu' lying in the lower regions of river Agano, Niigata prefecture, Japan, in 1966, with isolation of Rickettsia from the host and parasites	Acta Med. Biol. (Niigata)	1967	
Leptotrombidium pallidum	South Korea	Gangwon-do	37.927	127.742	1992-93	Immunofluorescence + PCR	Ree, H. I	Chang, W. H	Detection of Orientia tsutsugamushi DNA in individual trombiculids using polymerase chain reaction in Korea	Med. Entomol. Zool.	1997	
Leptotrombidium pallidum	South Korea	Gangwon-do	38.147	127.313	2005	PCR (56kDa)	Lee, H.I.	Shim, S.K.	Detection of Orientia tsutsugamushi, the causative agent of scrub typhus, in a novel mite	Vector Borne Zoonotic Dis.	2011	

									species, Euschengastia koreaensis, in Korea			
Leptotrombidium pallidum	Japan	Yamagata	38.309	140.149	2012	PCR (56kDa) & indirect IF	Seto, J.	Suzuki, Y	Poposed vector candidate: Leptotrombidium palpale for Shikokoshi type Orientia tsutsugamushi	Microbiol. Immunol.	2013	
Leptotrombidium pallidum	Japan	Akita	39.211	140.515	1969-70	Xenodiagnosis	Asanuma, K.	Kitaoka, M.	Occurrence of Scrub Typhus in clinical endemic area in Japan but not transmitted by Leptotrombidium akamushi	J. Med. Entomol.	1972	
Leptotrombidium pallidum	Japan	Akita	39.215	140.521	1970-71	Xenodiagnosis & complement fixation	Kitaoka, M.	Asanuma, K.	Experiments on chickens placed on ground in endemic of classical scrub typhus in Akita prefecture, Japan	J. Hyg. Epidemiol. Microbiol. Immunol.	1976	
Leptotrombidium pallidum	Japan	Akita	39.437	140.497	1964-66	Complement fixation	Kitaoka, M.	Okubo, K.	Epidemiological survey by means of complement fixation test on scrub typhus in Japan	Acta Med. Biol. (Niigata)	1967	
Leptotrombidium pallidum	Japan	Aomori	40.710	140.578	1975-76	Xenodiagnosis	Takada, N.		Epidemiology of Tsutsugamushi disease in Aomori Prefecture	Japanese Journal of Sanitary Zoology	1982	
Leptotrombidium pallidum	Russia	Primorsky Krai	42.430	130.678	1963-65	Xenodiagnosis & indirect IF	Kulagin, S.M.	Tarasevich, I.V.	On the natural focus of scrub typhus in the south of the Primorie area of the USSR	Acta Med. Biol. (Niigata)	1967	
Leptotrombidium pallidum	Russia	Primorsky Krai	42.459	130.655	1964-65	Direct immunofluorescence	Kudryashova, N.I.	Mirolyubova, L.N.	Natural infection of Trombiculid mites with the rickettsiae of tsutsugamushi disease in the Maritime Province	Meditsinskaya Parazitologiya i Parazitarnye Bolezni	1968	
Leptotrombidium pallidum	Russia	Primorsky Krai	42.459	130.655	1963	Xenodiagnosis	Kudryashova, N.I.	Tarasevich, I.V.	Trombiculids in a natural focus of tsutsugamushi disease in the south of the Maritime Province	Meditsinskaya Parazitologiya i Parazitarnye Bolezni	1964	
Leptotrombidium pallidum	Japan	Oita	32.233	131.606	1997-99	Nested PCR	Pham, X.D.	Otsuka, Y.	Detection of Orientia tsutsugamushi	J. Med. Entomol.	2001	Free living

									(Rickettsiales: Rickettsiaceae) in unengorged chiggers (Acari: Ttombiculidae) from Oita Prefecture, Japan, by nested polymerase chain reaction			
Leptotrombidium pallidum	Japan	Oita	33.199	131.517	1997-99	Nested PCR	Pham, X.D.	Otsuka, Y.	Detection of Orientia tsutsugamushi (Rickettsiales: Rickettsiaceae) in unengorged chiggers (Acari: Ttombiculidae) from Oita Prefecture, Japan, by nested polymerase chain reaction	J. Med. Entomol.	2001	Free living
Leptotrombidium pallidum	Japan	Shizuoka	35.322	138.897	1986-90	Xenodiagnosis & indirect IF	Kawamori, F.	Akiyama, M.	Epidemiology of Tsutsugamushi Disease in Relation to the Serotypes of Rickettsia tsutsugamushi Isolated from Patients, Field Mice, and Unfed Chiggers on the Eastern Slope of Mount Fuji, Shizuoka Prefecture, Japan	J. Clin. Microbiol.	1992	Free living
Leptotrombidium pallidum	Japan	Kyoto	35.550	135.130	1996-99	Indirect immunofluorescence	Takahashi, M.	Misumi, H.	Mite Vectors (Acari: Trombiculidae) of Scrub Typhus in a New Endemic Area in Northern Kyoto, Japan	J. Med. Entomol.	2004	Free living
Leptotrombidium pallidum	Japan	Kyoto	35.550	135.130	1997-01	xenodiagnosis/cell culture & indirect IFA	Misumi, H.	Takahashi, M.	Distribution of infective spots composed of unfed larvae infected with Orientia tsutsugamushi in Leptotrombidium mites and their annual fluctuations on the soil surface in an endemic area of tsutsugamushi disease (Acari: Trombiculidae)	Med. Entomol. Zool.	2002	Free living

Leptotrombidium pallidum	Japan	Kyoto	35.567	135.153	1997-98	Indirect immunofluorescence	Urakami, H	Takahashi, M	Detection, isolation and characterization of Orientia tsutsugamushi in Leptotrombidium intermedium	Med. Entomol. Zool.	2000	Free living
Leptotrombidium palpale	Japan	Oita	33.304	131.268	1964-66	complement fixation	Kitaoka, M.	Okubo, K.	Epidemiological survey by means of complement fixation test on scrub typhus in Japan	Acta Med. Biol. (Niigata)	1967	
Leptotrombidium palpale	South Korea	Jeollanam-do	34.997	127.044	1995	Immunofluorescence + PCR	Ree, H. I	Chang, W. H	Detection of Orientia tsutsugamushi DNA in individual trombiculids using polymerase chain reaction in Korea	Med. Entomol. Zool.	1997	
Leptotrombidium palpale	South Korea	Jeollanam-do	35.173	126.859	1992-93	Immunofluorescence + PCR	Ree, H. I	Chang, W. H	Detection of Orientia tsutsugamushi DNA in individual trombiculids using polymerase chain reaction in Korea	Med. Entomol. Zool.	1997	
Leptotrombidium palpale	South Korea	Jeollanam-do	35.200	126.502	1995	Immunofluorescence + PCR	Ree, H. I	Chang, W. H	Detection of Orientia tsutsugamushi DNA in individual trombiculids using polymerase chain reaction in Korea	Med. Entomol. Zool.	1997	
Leptotrombidium palpale	South Korea	Jeollanam-do	35.218	126.323	1995	Immunofluorescence + PCR	Ree, H. I	Chang, W. H	Detection of Orientia tsutsugamushi DNA in individual trombiculids using polymerase chain reaction in Korea	Med. Entomol. Zool.	1997	
Leptotrombidium palpale	South Korea	Jeollanam-do	35.264	126.476	1995	Immunofluorescence + PCR	Ree, H. I	Chang, W. H	Detection of Orientia tsutsugamushi DNA in individual trombiculids using polymerase chain reaction in Korea	Med. Entomol. Zool.	1997	
Leptotrombidium palpale	China	Shandong	35.266	117.977	1995-03	Xendiagnosis & PCR	Liu, Y.	Jia, N.	Consistency of the key genotypes of Orientia tsutsugamushi in scrub typhus patients, rodents, and chiggers from a New Endemic focus of Northern China	Cell Biochem. Biophys.	2013	

Leptotrombidium palpale	China	Shandong	35.266	117.977	1995-03	PCR (56kDa)	Liu, Y.	Jia, N.	Consistency of the key genotypes of Orientia tsutsugamushi in scrub typhus patients, rodents, and chiggers from a New Endemic focus of Northern China	Cell Biochem. Biophys.	2013	
Leptotrombidium palpale	China	Shandong	35.266	117.977	1995-96	Direct immunofluorescence	Liu, Y.X.	Wu, Q.Y.	First isolation of Rickettsia Tsutsugamushi from Leptotrombidium palpalis	Chinese Journal of Vector Biology and Control	1998	
Leptotrombidium palpale	China	Shandong	35.286	118.185	1995-96	Xenodiagnosis & indirect IF	Liu, Y.	Yang, Z.	Isolation of Rickettsia tsutsugamushi from Trombiculid mites (Acari: Trombiculidae) in Feixian county, Shandong province, China	Entomologia Sinica	1999	
Leptotrombidium palpale	South Korea	Jeollanam-do	35.304	126.786	2009	Nested PCR	Choi, Y.J.	Lee, I.Y.	Geographical distribution of Orientia tsutsugamushi strains in chiggers from three provinces of Korea	Microbiol. Immunol.	2018	
Leptotrombidium palpale	China	Shandong	35.362	118.090	1995-96	Xenodiagnosis & indirect IF	Liu, Y.	Yang, Z.	Epidemiological study of autumn-winter type scrub typhus in a new endemic focus of Fei County, Shandong Province, China	Syst. Appl. Acarol.	2000	
Leptotrombidium palpale	South Korea	Gyeongnam-do	35.567	128.166	2005	PCR (56kDa)	Lee, H.I.	Shim, S.K.	Detection of Orientia tsutsugamushi, the causative agent of scrub typhus, in a novel mite species, Euschengastia koreaensis, in Korea	Vector Borne Zoonotic Dis.	2011	
Leptotrombidium palpale	China	Shandong	35.879	117.928	1995-02	Xendiagnosis & PCR	Liu, Y.	Zhao, Z.	Characterization of Orientia tsutsugamushi strains isolated in Shandong Province, China by immunofluorescence and restriction fragment length polymorphism (RFLP) analyses	Southeast Asian J. Trop. Med. Public Health	2004	
Leptotrombidium palpale	China	Shandong	35.924	117.764	2010-12	PCR (56kDa)	Zhang, M.	Zhao, Z.T.	Molecular epidemiology of Orientia tsutsugamushi	Parasit. Vectors	2013	

									in chiggers and ticks from domestic rodents in Shandong, northern China			
Leptotrombidium palpale	South Korea	Jeollabuk-do	35.960	126.995	1992-93	Immunofluorescence + PCR	Ree, H. I	Chang, W. H	Detection of Orientia tsutsugamushi DNA in individual trombiculids using polymerase chain reaction in Korea	Med. Entomol. Zool.	1997	
Leptotrombidium palpale	South Korea	Jeollabuk-do	36.029	126.929	1992-93	Immunofluorescence + PCR	Ree, H. I	Chang, W. H	Detection of Orientia tsutsugamushi DNA in individual trombiculids using polymerase chain reaction in Korea	Med. Entomol. Zool.	1997	
Leptotrombidium palpale	Japan	Yamagata	38.309	140.149	2012	56kDa & nested PCR, sequencing, indirect immunofluorescence	Seto, J.	Suzuki, Y	Poposed vector candidate: Leptotrombidium palpale for Shikokoshi type Orientia tsutsugamushi	Microbiol. Immunol.	2013	
Leptotrombidium palpale	Russia	Primorsky Krai	42.872	131.365	1963-65	Xenodiagnosis & microscopy	Kulagin, S.M.	Tarasevich, I.V.	The investigation of scrub typhus in the USSR	J. Hyg. Epidemiol. Microbiol.	1968	
Leptotrombidium palpale	Russia	Primorsky Krai	42.936	131.375	1964-65	Direct immunofluorescence	Kudryashova, N.I.	Mirolyubova, L.N.	Natural infection of Trombiculid mites with the rickettsiae of tsutsugamushi disease in the Maritime Province	Meditsinskaya Parazitologiya i Parazitarnye Bolezni	1968	
Leptotrombidium pavlovskyi	Russia	Primorsky Krai	42.429	130.633	1964-65	Direct immunofluorescence	Kudryashova, N.I.	Mirolyubova, L.N.	Natural infection of Trombiculid mites with the rickettsiae of tsutsugamushi disease in the Maritime Province	Meditsinskaya Parazitologiya i Parazitarnye Bolezni	1968	
Leptotrombidium pavlovskyi	Russia	Primorsky Krai	42.430	130.678	1963-65	Xenodiagnosis & indirect IF	Kulagin, S.M.	Tarasevich, I.V.	On the natural focus of scrub typhus in the south of the Primorie area of the USSR	Acta Med. Biol. (Niigata)	1967	
Leptotrombidium pavlovskyi	Russia	Primorsky Krai	42.683	130.760	1964-65	Direct immunofluorescence	Kudryashova, N.I.	Mirolyubova, L.N.	Natural infection of Trombiculid mites with the rickettsiae of tsutsugamushi disease in the Maritime Province	Meditsinskaya Parazitologiya i Parazitarnye Bolezni	1968	
Leptotrombidium	Russia	Primorsky Krai	42.683	130.760	1963	Xenodiagnosis	Kudryashova,	Tarasevich, I.V.	Trombiculids in a natural	Meditsinskaya	1964	

pavlovskyi							N.I.		focus of tsutsugamushi disease in the south of the Maritime Province	Parazitologiya i Parazitarnye Bolezni		
Leptotrombidium pavlovskyi	Russia	Primorsky Krai	42.685	130.663	1964-65	Direct immunofluorescence	Kudryashova, N.I.	Mirolyubova, L.N.	Natural infection of Trombiculid mites with the rickettsiae of tsutsugamushi disease in the Maritime Province	Meditsinskaya Parazitologiya i Parazitarnye Bolezni	1968	
Leptotrombidium pavlovskyi	Russia	Primorsky Krai	42.872	131.365	1963-65	Xenodiagnosis & microscopy	Kulagin, S.M.	Tarasevich, I.V.	The investigation of scrub typhus in the USSR	J. Hyg. Epidemiol. Microbiol.	1968	
Leptotrombidium pavlovskyi	Russia	Primorsky Krai	42.429	130.633	1963	Xenodiagnosis	Kudryashova, N.I.	Tarasevich, I.V.	Trombiculids in a natural focus of tsutsugamushi disease in the south of the Maritime Province	Meditsinskaya Parazitologiya i Parazitarnye Bolezni	1964	Free living
Leptotrombidium peniculatum	*Thailand*	Nakhon Ratchasima	14.957	102.111	1977-78	Direct immunofluorescence	Shirai, A.	Tanskul, P.L.	Rickettsia tsutsugamushi strains found in chiggers collected in Thailand	Southeast Asian J. Trop. Med. Public Health	1981	Free living
Leptotrombidium scutellare	*China*	Fujian	24.440	118.330	1999-00	Nested PCR	Wang, H.C.	Chung, C.L.	Studies on the vectors and pathogens of scrub typhus on murine-like animals in Kinmen Country, Taiwan	Formosan Entomol.	2004	
Leptotrombidium scutellare	*China*	Fujian	25.544	118.760	1997-98	Nested PCR	Yan, Y.S.	Zheng, J.	Detection of Orienta tsutsugamushi in chigger mites collected in Fujian coastal and mountain areas in recent years.	Chinese Journal of Zoonoses	1999	
Leptotrombidium scutellare	*China*	Jiangsu	32.868	120.320	1994	PCR (56kDa)	Guo, H.B.	Wu, G.H.	Studies on detection of Rickettsia tsutsugamushi DNA in the single larva of Leptotrombidium (L.) scutellare collected from the endemic areas by PCR	Chinese Journal of Zoonoses	1996	
Leptotrombidium scutellare	*South Korea*	Cheju Island	33.480	126.384	1991-92	Indirect immunofluorescence	Ree, H.I.	Lee, I.Y	Study on vector mites of tsutsugamushi disease in Cheju Island, Korea	Korean J. Parasitol.	1992	
Leptotrombidium scutellare	*South Korea*	Cheju Island	33.488	126.416	1991-92	Indirect immunofluorescence	Ree, H.I.	Lee, I.Y	Study on vector mites of tsutsugamushi disease in Cheju Island, Korea	Korean J. Parasitol.	1992	
Leptotrombidium	*South*	Jeollanam-do	34.673	126.925	1992-93	Immunofluorescence	Ree, H. I	Chang, W. H	Detection of Orientia	Med. Entomol.	1997	

scutellare	*Korea*					+ PCR			tsutsugamushi DNA in individual trombiculids using polymerase chain reaction in Korea	Zool.		
Leptotrombidium scutellare	*South Korea*	Jeollanam-do	34.772	127.662	1992-93	Immunofluorescence + PCR	Ree, H. I	Chang, W. H	Detection of Orientia tsutsugamushi DNA in individual trombiculids using polymerase chain reaction in Korea	Med. Entomol. Zool.	1997	
Leptotrombidium scutellare	*Japan*	Chiba	34.907	139.899	1952-61	Xenodiagnosis	Asanuma, K.	Okubo, K.	Determination of the vector mites of scrub typhus in Japan	Jap. J. Med. Sci. Biol.	1962	
Leptotrombidium scutellare	*South Korea*	Jeollanam-do	34.997	127.044	1995	Immunofluorescence + PCR	Ree, H. I	Chang, W. H	Detection of Orientia tsutsugamushi DNA in individual trombiculids using polymerase chain reaction in Korea	Med. Entomol. Zool.	1997	
Leptotrombidium scutellare	*Japan*	Chiba	35.135	139.866	1952	Xenodiagnosis	Asanuma, K.	Kitaoka, M.	Evidences for Trombicula scutellaris to be a vector of scrub typhus in Chiba prefecture, Japan	Japanese Journal of Sanitary Zoology	1959	
Leptotrombidium scutellare	*South Korea*	Jeollanam-do	35.173	126.859	1992-93	Immunofluorescence + PCR	Ree, H. I	Chang, W. H	Detection of Orientia tsutsugamushi DNA in individual trombiculids using polymerase chain reaction in Korea	Med. Entomol. Zool.	1997	
Leptotrombidium scutellare	*South Korea*	Jeollanam-do	35.193	126.829	2014-15	Immunofluorescence + PCR	Park, J. W	Chung, J.K	Seroepidemiological Survey of Zoonotic Diseases in Small Mammals with PCR Detection of Orientia tsutsugamushi in Chiggers, Gwangju, Korea	Korean J. Parasitol.	2016	
Leptotrombidium scutellare	*South Korea*	Gwangju	35.193	126.829	2014-16	PCR (56kDa)	Park, J.W.	Kim, S.H.	Molecular epidemiology of an Orientia tsutsugamushi gene encoding 56-kDa type-specific antigen in chiggers, small mammals and patients from Southwest region of Korea	Am. J. Trop. Med. Hyg.	2018	
Leptotrombidium scutellare	*South Korea*	Jeollanam-do	35.200	126.502	1995	Immunofluorescence + PCR	Ree, H. I	Chang, W. H	Detection of Orientia tsutsugamushi DNA in individual trombiculids using polymerase chain reaction in Korea	Med. Entomol. Zool.	1997	

Leptotrombidium scutellare	*South Korea*	Jeollanam-do	35.218	126.323	1995	Immunofluorescence + PCR	Ree, H. I	Chang, W. H	Detection of Orientia tsutsugamushi DNA in individual trombiculids using polymerase chain reaction in Korea	Med. Entomol. Zool.	1997	
Leptotrombidium scutellare	*South Korea*	Jeollanam-do	35.264	126.476	1995	Immunofluorescence + PCR	Ree, H. I	Chang, W. H	Detection of Orientia tsutsugamushi DNA in individual trombiculids using polymerase chain reaction in Korea	Med. Entomol. Zool.	1997	
Leptotrombidium scutellare	*China*	Shandong	35.266	117.977	1995-03	Xendiagnosis & PCR	Liu, Y.	Jia, N.	Consistency of the key genotypes of Orientia tsutsugamushi in scrub typhus patients, rodents, and chiggers from a New Endemic focus of Northern China	Cell Biochem. Biophys.	2013	
Leptotrombidium scutellare	*China*	Shandong	35.266	117.977	1995-03	PCR (56kDa)	Liu, Y.	Jia, N.	Consistency of the key genotypes of Orientia tsutsugamushi in scrub typhus patients, rodents, and chiggers from a New Endemic focus of Northern China	Cell Biochem. Biophys.	2013	
Leptotrombidium scutellare	*China*	Shandong	35.286	118.185	1995-96	Xenodiagnosis & indirect IF	Liu, Y.	Yang, Z.	Isolation of Rickettsia tsutsugamushi from Trombiculid mites (Acari: Trombiculidae) in Feixian county, Shandong province, China	Entomologia Sinica	1999	
Leptotrombidium scutellare	*Japan*	Shizuoka	35.294	138.854	1952-60	Xenodiagnosis	Asanuma, K.	Okubo, K.	Determination of the vector mites of scrub typhus in Japan	Jap. J. Med. Sci. Biol.	1962	
Leptotrombidium scutellare	*Japan*	Shizuoka	35.294	138.854	1954-62	Xenodiagnosis	Asanuma, K.	Kitaoka, M.	Leptotrombidium scutellare as a vector of scrub typhus at the endemic area of the foothills of Mt. Fuji, Japan	J. Hyg. Epidemiol. Microbiol. Immunol.	1974	
Leptotrombidium scutellare	*South Korea*	Jeollanam-do	35.304	126.786	2009	Nested PCR	Choi, Y.J.	Lee, I.Y.	Geographical distribution of Orientia tsutsugamushi strains in chiggers from three provinces of Korea	Microbiol. Immunol.	2018	
Leptotrombidium scutellare	*China*	Shandong	35.362	118.090	1995-96	Xenodiagnosis & indirect IF	Liu, Y.	Yang, Z.	Epidemiological study of autumn-winter type scrub typhus in a new endemic	Syst. Appl. Acarol.	2000	

									focus of Fei County, Shandong Province, China			
Leptotrombidium scutellare	*South Korea*	Gyeongnam-do	35.567	128.166	2005	PCR (56kDa)	Lee, H.I.	Shim, S.K.	Detection of Orientia tsutsugamushi, the causative agent of scrub typhus, in a novel mite species, Euschengastia koreaensis, in Korea	Vector Borne Zoonotic Dis.	2011	
Leptotrombidium scutellare	*China*	Shandong	35.757	117.946	1986	Xenodiagnosis & indirect IF	Xiangrui, C.	Jinju, W.	Recent studies on scrub typhus and Rickettsia tsutsugamushi in Shandong Province - China	Eur. J. Epidemiol.	1991	
Leptotrombidium scutellare	*China*	Shandong	35.879	117.928	?	Xendiagnosis & PCR	Liu, Y.	Zhao, Z.	Characterization of Orientia tsutsugamushi strains isolated in Shandong Province, China by immunofluorescence and restriction fragment length polymorphism (RFLP) analyses	Southeast Asian J. Trop. Med. Public Health	2004	
Leptotrombidium scutellare	*China*	Shandong	35.924	117.764	2010-12	PCR (56kDa)	Zhang, M.	Zhao, Z.T.	Molecular epidemiology of Orientia tsutsugamushi in chiggers and ticks from domestic rodents in Shandong, northern China	Parasit. Vectors	2013	
Leptotrombidium scutellare	*South Korea*	Jeollabuk-do	35.960	126.995	1992-93	Immunofluorescence + PCR	Ree, H. I	Chang, W. H	Detection of Orientia tsutsugamushi DNA in individual trombiculids using polymerase chain reaction in Korea	Med. Entomol. Zool.	1997	
Leptotrombidium scutellare	*South Korea*	Jeollabuk-do	36.029	126.929	1992-93	Immunofluorescence + PCR	Ree, H. I	Chang, W. H	Detection of Orientia tsutsugamushi DNA in individual trombiculids using polymerase chain reaction in Korea	Med. Entomol. Zool.	1997	
Leptotrombidium scutellare	*South Korea*	Gyeongsangbuk-do	36.454	129.380	1992-93	Immunofluorescence + PCR	Ree, H. I	Chang, W. H	Detection of Orientia tsutsugamushi DNA in individual trombiculids using polymerase chain reaction in Korea	Med. Entomol. Zool.	1997	
Leptotrombidium	*South*	Chungcheongnam-	36.762	126.868	2009	Nested PCR	Choi, Y.J.	Lee, I.Y.	Geographical distribution of	Microbiol.	2018	

scutellare	*Korea*	do							Orientia tsutsugamushi strains in chiggers from three provinces of Korea	Immunol.		
Leptotrombidium scutellare	*South Korea*	Chungcheongnam-do	37.040	126.870	2009	Nested PCR	Choi, Y.J.	Lee, I.Y.	Geographical distribution of Orientia tsutsugamushi strains in chiggers from three provinces of Korea	Microbiol. Immunol.	2018	
Leptotrombidium scutellare	*China*	Shandong	37.167	117.834	1995-03	PCR (56kDa)	Liu, Y.	Jia, N.	Consistency of the key genotypes of Orientia tsutsugamushi in scrub typhus patients, rodents, and chiggers from a New Endemic focus of Northern China	Cell Biochem. Biophys.	2013	
Leptotrombidium scutellare	*Malaysia*	Pahang	4.502	101.390	?	Indirect immunofluorescence	Shirai, A.	Dohany, A.L.	Serological classification of Rickettsia tsutsugamushi organisms found in chiggers (Acarina: Trombiculidae) collected in Peninsular Malaysia	Trans. R. Soc. Trop. Med. Hyg.	1981	Free living
Leptotrombidium scutellare	*Thailand*	Nakhon Ratchasima	14.957	102.111	1977-78	Direct immunofluorescence	Shirai, A.	Tanskul, P.L.	Rickettsia tsutsugamushi strains found in chiggers collected in Thailand	Southeast Asian J. Trop. Med. Public Health	1981	Free living
Leptotrombidium scutellare	*Thailand*	Chiang Mai	18.706	98.982	1977-78	Direct immunofluorescence	Shirai, A.	Tanskul, P.L.	Rickettsia tsutsugamushi strains found in chiggers collected in Thailand	Southeast Asian J. Trop. Med. Public Health	1981	Free living
Leptotrombidium scutellare	*Japan*	Kagoshima	31.700	130.616	2000-01	Direct immunofluorescence	Takahashi, M.	Urakami, H.	Detection and serotyping of Orientia tsutsugamushi from the unfed larval trombiculid mite Leptotrombidium scutellare (Nagayo, Miyagawa, Mitamura, Tamiya, et Tenjin, 1921) (Acari: Trombiculidae)	Med. Entomol. Zool.	2002	Free living
Leptotrombidium scutellare	*China*	Jiangsu	32.600	119.940	2013	Nested PCR	He, Y.L.	Yang, H.Y.	Study of epidemic area on Tsutsugamushi disease in Taizhou from 2013 to 2014	Chinese Journal of Preventive Medicine	2017	Free living
Leptotrombidium scutellare	*Japan*	Oita	32.967	131.400	1997-99	Nested PCR	Pham, X.D.	Otsuka, Y.	Detection of Orientia tsutsugamushi (Rickettsiales:	J. Med. Entomol.	2001	Free living

									Rickettsiaceae) in unengorged chiggers (Acari: Ttombiculidae) from Oita Prefecture, Japan, by nested polymerase chain reaction			
Leptotrombidium scutellare	*Japan*	Shizuoka	35.322	138.897	1986-90	Xenodiagnosis & indirect IF	Kawamori, F.	Akiyama, M.	Epidemiology of Tsutsugamushi Disease in Relation to the Serotypes of Rickettsia tsutsugamushi Isolated from Patients, Field Mice, and Unfed Chiggers on the Eastern Slope of Mount Fuji, Shizuoka Prefecture, Japan	J. Clin. Microbiol.	1992	Free living
Leptotrombidium sp.	*Indonesia*	East Kalimantan	-1.023	116.865	2007-08	PCR & ELISA	Widjaja, S.	Williams, M.	Geographical assessment of Rickettsioses in Indonesia	Vector Borne Zoonotic Dis.	2016	
Leptotrombidium sp.	*Indonesia*	North Sulawesi	1.447	124.813	2007-08	ELISA	Widjaja, S.	Williams, M.	Geographical assessment of Rickettsioses in Indonesia	Vector Borne Zoonotic Dis.	2016	
Leptotrombidium sp.	*Thailand*	Phang Nga	8.664	98.452	2015	PCR (56kDa)	Takhampunya, R.	Korkusol, A.	Heterogeneity of Orientia tsutsugamushi genotypes in field-collected trombiculid mites from wild-caught small mammals in Thailand	Plos Negl. Trop. Dis.	2018	
Leptotrombidium sp.	*Thailand*	Chachoengsao	13.786	101.092	2010	Xendiagnosis & PCR	Takhampunya, R.	Tippayachai, B.	Characterization based on the 56kDa type-specific antigen gene of Orientia tsutsugamushi genotypes isolated from Leptotrombidium mites and the rodent host post-infection	Am. J. trop. Med. Hyg.	2014	
Leptotrombidium sp.	*Thailand*	Multiple sites	15.200	102.100	2017-18	PCR (56kDa)	Linsuwananon, P.	Krairojananan, P.	Surveillance for scrub typhus, Rickettsial diseases, and Leptospirosis in US and multinational military training exercise Cobra Gold Sites in Thailand	US Army Med. Dep. J.	2018	
Leptotrombidium sp.	*Taiwan*	Hualien	23.923	121.566	2007-08	PCR (56kDa)	Kuo, C.C.	Wang, H.C.	The potential effect of exotic Pacific rats Rattus exulans on vectors of scrub typhus	J. Appl. Ecol.	2011	
Leptotrombidium sp.	Taiwan	Hualien	24.100	121.570	2007-08	PCR (56kDa)	Kuo, C.C.	Huang, J.L.	Cascading effect of economic globalization on	Ecol. Appl.	2012	

									human risks of scrub typhus and tick-borne rickettsial diseases.			
Leptotrombidium sp.	Pakistan	Gilgit-Baltistan	34.907	73.654	1962-65	Xenodiagnosis	Traub, R.	Wisseman, C.L.	The occurrence of scrub typhus in unusual habitats in West Pakistan	Trans. R. Soc. Trop. Med. Hyg.	1967	
Leptotrombidium sp.	Thailand	Chiang Mai	18.706	98.982	1977-78	Direct immunofluorescence	Shirai, A.	Tanskul, P.L.	Rickettsia tsutsugamushi strains found in chiggers collected in Thailand	Southeast Asian J. Trop. Med. Public Health	1981	Free living
Leptotrombidium tachensis	China	Zhejiang	29.180	120.089	?	?	Fan, M.Y.	Walker, D.H.	Epidemiology and ecology of rickettsial diseases in the People's Republic of China	Rev. Infect. Dis.	1987	
Leptotrombidium taishanicum	China	Shandong	35.924	117.764	2010-12	PCR (56kDa)	Zhang, M.	Zhao, Z.T.	Molecular epidemiology of Orientia tsutsugamushi in chiggers and ticks from domestic rodents in Shandong, northern China	Parasit. Vectors	2013	
Leptotrombidium waiganmensis	China	Zhejiang	29.180	120.089	?	?	Fan, M.Y.	Walker, D.H.	Epidemiology and ecology of rickettsial diseases in the People's Republic of China	Rev. Infect. Dis.	1987	
Leptotrombidium zetum	South Korea	Gyeongnam-do	34.851	128.429	1992-93	PCR (56kDa) & indirect IF	Ree, H. I	Chang, W. H	Detection of Orientia tsutsugamushi DNA in individual trombiculids using polymerase chain reaction in Korea	Med. Entomol. Zool.	1997	
Leptotrombidium zetum	South Korea	Jeollabuk-do	35.960	126.995	1992-93	PCR (56kDa) & indirect IF	Ree, H. I	Chang, W. H	Detection of Orientia tsutsugamushi DNA in individual trombiculids using polymerase chain reaction in Korea	Med. Entomol. Zool.	1997	
Lorillatum sp.	Thailand	Sisaket	14.477	104.490	2015	PCR (56kDa)	Takhampunya, R.	Korkusol, A.	Heterogeneity of Orientia tsutsugamushi genotypes in field-collected trombiculid mites from wild-caught small mammals in Thailand	Plos Negl. Trop. Dis.	2018	
Microtrombicula chamlongi	Thailand	Nakhon Ratchasima	14.957	102.111	1977-78	Direct immunofluorescence	Shirai, A.	Tanskul, P.L.	Rickettsia tsutsugamushi strains found in chiggers	Southeast Asian J. Trop.	1981	Free living

									collected in Thailand	Med. Public Health		
Neoschoengastia sp.	China	Zhejiang	29.180	120.089	?	?	Fan, M.Y.	Walker, D.H.	Epidemiology and ecology of rickettsial diseases in the People's Republic of China	Rev. Infect. Dis.	1987	
Neotrombicula japonica	South Korea	Gyeonggi-do	37.760	126.780	2005	PCR (56kDa)	Lee, H.I.	Shim, S.K.	Detection of Orientia tsutsugamushi, the causative agent of scrub typhus, in a novel mite species, Euschengastia koreaensis, in Korea	Vector Borne Zoonotic Dis.	2011	
Neotrombicula japonica	South Korea	Gangwon-do	38.147	127.313	2005	PCR (56kDa)	Lee, H.I.	Shim, S.K.	Detection of Orientia tsutsugamushi, the causative agent of scrub typhus, in a novel mite species, Euschengastia koreaensis, in Korea	Vector Borne Zoonotic Dis.	2011	
Neotrombicula japonica	Russia	Primorsky Krai	42.429	130.633	1964-65	Direct immunofluorescence	Kudryashova, N.I.	Mirolyubova, L.N.	Natural infection of Trombiculid mites with the rickettsiae of tsutsugamushi disease in the Maritime Province	Meditsinskaya Parazitologiya i Parazitarnye Bolezni	1968	
Neotrombicula japonica	Russia	Primorsky Krai	42.430	130.678	1963-65	Xenodiagnosis & indirect IF	Kulagin, S.M.	Tarasevich, I.V.	On the natural focus of scrub typhus in the south of the Primorie area of the USSR	Acta Med. Biol. (Niigata)	1967	
Neotrombicula japonica	Russia	Primorsky Krai	42.459	130.655	1964-65	Direct immunofluorescence	Kudryashova, N.I.	Mirolyubova, L.N.	Natural infection of Trombiculid mites with the rickettsiae of tsutsugamushi disease in the Maritime Province	Meditsinskaya Parazitologiya i Parazitarnye Bolezni	1968	
Neotrombicula japonica	Russia	Primorsky Krai	42.685	130.663	1964-65	Direct immunofluorescence	Kudryashova, N.I.	Mirolyubova, L.N.	Natural infection of Trombiculid mites with the rickettsiae of tsutsugamushi disease in the Maritime Province	Meditsinskaya Parazitologiya i Parazitarnye Bolezni	1968	
Neotrombicula japonica	Russia	Primorsky Krai	42.872	131.365	1963-65	Xenodiagnosis & microscopy	Kulagin, S.M.	Tarasevich, I.V.	The investigation of scrub typhus in the USSR	J. Hyg. Epidemiol. Microbiol.	1968	
Neotrombicula	Russia	Primorsky Krai	42.936	131.375	1964-65	Direct	Kudryashova,	Mirolyubova, L.N.	Natural infection of	Meditsinskaya	1968	

japonica						immunofluorescence	N.I.		Trombiculid mites with the rickettsiae of tsutsugamushi disease in the Maritime Province	Parazitologiya i Parazitarnye Bolezni		
Neotrombicula japonica	Russia	Primorsky Krai	43.022	131.860	1964-65	Direct immunofluorescence	Kudryashova, N.I.	Mirolyubova, L.N.	Natural infection of Trombiculid mites with the rickettsiae of tsutsugamushi disease in the Maritime Province	Meditsinskaya Parazitologiya i Parazitarnye Bolezni	1968	
Neotrombicula japonica	Japan	Oita	32.967	131.400	1997-99	Nested PCR	Pham, X.D.	Otsuka, Y.	Detection of Orientia tsutsugamushi (Rickettsiales: Rickettsiaceae) in unengorged chiggers (Acari: Ttombiculidae) from Oita Prefecture, Japan, by nested polymerase chain reaction	J. Med. Entomol.	2001	Free living
Neotrombicula microti	Russia	Sakhalin Oblast	43.795	146.747	1968-73	Xenodiagnosis	Somov, G.P.	Shubin, F.N.	Tsutsugamushi fever in the Kuril Islands	Zh. Mikrobiol. Epidemiol. Immunobiol.	1976	
Neotrombicula microti	Russia	Sakhalin Oblast	43.795	146.747	?	Xenodiagnosis	Somov, G.P.	Shubin, F.N.	A major result of studying Tsutsugamushi fever in the Soviet Far East	Folia Microbiol.	1976	
Neotrombicula mitamurai	Russia	Primorsky Krai	42.430	130.678	1963-65	Xenodiagnosis & indirect IF	Kulagin, S.M.	Tarasevich, I.V.	On the natural focus of scrub typhus in the south of the Primorie area of the USSR	Acta Med. Biol. (Niigata)	1967	
Neotrombicula mitamurai	Russia	Primorsky Krai	42.683	130.760	1964-65	Direct immunofluorescence	Kudryashova, N.I.	Mirolyubova, L.N.	Natural infection of Trombiculid mites with the rickettsiae of tsutsugamushi disease in the Maritime Province	Meditsinskaya Parazitologiya i Parazitarnye Bolezni	1968	
Neotrombicula mitamurai	Russia	Primorsky Krai	42.685	130.663	1963	Xenodiagnosis	Kudryashova, N.I.	Tarasevich, I.V.	Trombiculids in a natural focus of tsutsugamushi disease in the south of the Maritime Province	Meditsinskaya Parazitologiya i Parazitarnye Bolezni	1964	
Neotrombicula mitamurai	Russia	Primorsky Krai	42.872	131.365	1963-65	Xenodiagnosis & microscopy	Kulagin, S.M.	Tarasevich, I.V.	The investigation of scrub typhus in the USSR	J. Hyg. Epidemiol. Microbiol.	1968	
Neotrombicula	Russia	Primorsky Krai	42.936	131.375	1964-65	Direct	Kudryashova,	Mirolyubova, L.N.	Natural infection of	Meditsinskaya	1968	

mitamurai						immunofluorescence	N.I.		Trombiculid mites with the rickettsiae of tsutsugamushi disease in the Maritime Province	Parazitologiya i Parazitarnye Bolezni		
Neotrombicula nagayoi	Russia	Sakhalin Oblast	44.150	145.875	1973	Xenodiagnosis	Somov, G.P.	Shubin, F.N.	Tsutsugamushi fever in the Kuril Islands	Zh. Mikrobiol. Epidemiol. Immunobiol.	1976	
Neotrombicula nagayoi	Russia	Sakhalin Oblast	44.150	145.875	?	Xenodiagnosis	Somov, G.P.	Shubin, F.N.	A major result of studying Tsutsugamushi fever in the Soviet Far East	Folia Microbiol.	1976	
Neotrombicula pomeranzevi	Japan	Hokkaido	43.058	141.476	1959-68	Xenodiagnosis & complement fixation	Kitaoka, M.	Asanuma, K,	Seasonal occurrence of trombiculid mites species and Leptotrombidium kawamurai (Acarina, Trombiculidae) as a carrier of Rickettsia orientalis in the Nopporo area, Hokkaido, Japan.	J. Hyg. Epidemiol. Microbiol. Immunol.	1973	
Neotrombicula pomeranzevi	Russia	Sakhalin Oblast	43.795	146.747	1968-73	Xenodiagnosis	Somov, G.P.	Shubin, F.N.	Tsutsugamushi fever in the Kuril Islands	Zh. Mikrobiol. Epidemiol. Immunobiol.	1976	
Neotrombicula pomeranzevi	Russia	Sakhalin Oblast	43.795	146.747	?	Xenodiagnosis	Somov, G.P.	Shubin, F.N.	A major result of studying Tsutsugamushi fever in the Soviet Far East	Folia Microbiol.	1976	
Neotrombicula shiraii	Japan	Niigata	38.112	138.393	1957-58	Xenodiagnosis & microscopy	Saito, Y.	Otsuru, M.	Notes on Trombiculid mites collected in Sadi Island of Japan and isolation of rickettsia, with a description of Trombicula (Noetrombicula) sadoensis n. sp.	Acta Med. Biol. (Niigata)	1959	
Odontacarus sp.	Thailand	Nakhon Ratchasima	14.957	102.111	1977-78	Direct immunofluorescence	Shirai, A.	Tanskul, P.L.	Rickettsia tsutsugamushi strains found in chiggers collected in Thailand	Southeast Asian J. Trop. Med. Public Health	1981	Free living
Ornithonyssus bacoti	India	Maharashtra	21.146	79.086	2016	PCR (56kDa)	Bhate, R.	Pansare,N.	Prevalence and phylogenetic analysis of Orientia tsutsugamushi in rodents and mites from Central India	Vector Borne Zoonotic Dis.	2017	

Schoengastia sp.	Thailand	Buriram	14.300	102.800	2017-18	PCR (56kDa)	Linsuwananon, P.	Krairojananan, P.	Surveillance for scrub typhus, Rickettsial diseases, and Leptospirosis in US and multinational military training exercise Cobra Gold Sites in Thailand	US Army Med. Dep. J.	2018	
Trombicula sadoensis n. sp.	Japan	Niigata	38.112	138.393	1957-58	Xenodiagnosis & microscopy	Saito, Y.	Otsuru, M.	Notes on Trombiculid mites collected in Sadi Island of Japan and isolation of rickettsia, with a description of Trombicula (Noetrombicula) sadoensis n. sp.	Acta Med. Biol. (Niigata)	1959	
Trombicula tosa	Japan	Kochi	33.720	133.550	1952-60	Xenodiagnosis	Asanuma, K.	Okubo, K.	Determination of the vector mites of scrub typhus in Japan	Jap. J. Med. Sci. Biol.	1962	
Walchia chinensis	China	Fujian	24.440	118.330	1999-00	Nested PCR	Wang, H.C.	Chung, C.L.	Studies on the vectors and pathogens of scrub typhus on murine-like animals in Kinmen Country, Taiwan	Formosan Entomol.	2004	
Walchia chinensis	China	Fujian	25.544	118.760	1997-98	Nested PCR	Yan, Y.S.	Zheng, J.	Detection of Orienta tsutsugamushi in chigger mites collected in Fujian coastal and mountain areas in recent years.	Chinese Journal of Zoonoses	1999	
Walchia chinensis	China	Zhejiang	29.180	120.089	?	?	Fan, M.Y.	Walker, D.H.	Epidemiology and ecology of rickettsial diseases in the People's Republic of China	Rev. Infect. Dis.	1987	
Walchia chinensis	China	Hainan	16.330	112.026	2000	PCR (56kDa)	Wang, S.S.	Zhan, D.C.	Sequence analysis of Orientia tsutsugamushi DNA from mites collected in Xisa archipelago, China	Southeast Asian J. Trop. Med. Public Health	2002	
Walchia pacifica	China	Shandong	35.266	117.977	1995-03	Xendiagnosis & PCR	Liu, Y.	Jia, N.	Consistency of the key genotypes of Orientia tsutsugamushi in scrub typhus patients, rodents, and chiggers from a New Endemic focus of	Cell Biochem. Biophys.	2013	

									Northern China			
Walchia pacifica	China	Shandong	35.266	117.977	1995-03	PCR (56kDa)	Liu, Y.	Jia, N.	Consistency of the key genotypes of Orientia tsutsugamushi in scrub typhus patients, rodents, and chiggers from a New Endemic focus of Northern China	Cell Biochem. Biophys.	2013	
Walchia pacifica	China	Shandong	35.286	118.185	1995-96	Xenodiagnosis & indirect IF	Liu, Y.	Yang, Z.	Isolation of Rickettsia tsutsugamushi from Trombiculid mites (Acari: Trombiculidae) in Feixian county, Shandong province, China	Entomologia Sinica	1999	
Walchia pacifica	China	Shandong	35.362	118.090	1995-96	Xenodiagnosis & indirect IF	Liu, Y.	Yang, Z.	Epidemiological study of autumn-winter type scrub typhus in a new endemic focus of Fei County, Shandong Province, China	Syst. Appl. Acarol.	2000	
Walchia pacifica	China	Shandong	35.879	117.928	?	Xendiagnosis & PCR	Liu, Y.	Zhao, Z.	Characterization of Orientia tsutsugamushi strains isolated in Shandong Province, China by immunofluorescence and restriction fragment length polymorphism (RFLP) analyses	Southeast Asian J. Trop. Med. Public Health	2004	

Additional file 1: Table S6. Non-human vertebrate species tested for *O. tsutsugamushi* (all laboratory tests combined)

Group	Host species	Total Tested	Total Positive	Percentage positive
Artiodactyla	*Bos taurus* (cow)	706	12	1.7
Artiodactyla	*Capra aegagrus hircus* (goat)	292	25	8.6
Artiodactyla	*Sus scrofa* (domestic pig)	60	1	1.7
Artiodactyla	*Ovis aries* (sheep)	510	16	3.1
Aves	"Birds"	150	0	0.0
Aves	*Carpodacus sibircus*	?	1	-
Aves	*Gallus gallus domesticus* (chicken)	13	4	30.8
Aves	*Climacteris* sp.	1	0	0.0
Aves	*Cracticus nigrogularis*	1	0	0.0
Aves	*Cracticus torquatus*	1	0	0.0
Aves	*Dacelo gigas*	1	0	0.0
Aves	*Emberiza spodocephala*	?	2	-
Aves	*Eopsaltria chrysorrhoa*	4	0	0.0
Aves	*Macropygia phasianella*	1	0	0.0
Aves	*Motacilla cinerea*	16	3	18.8
Aves	*Myzantha melanocephala*	4	0	0.0
Aves	*Philemon corniculatus*	2	0	0.0
Aves	*Passer domesticus*	91	5	5.5
Aves	*Strepera graculina*	1	1	100.0
Aves	*Trichoglossus chlorolepidoptus*	7	0	0.0
Canidae	*Cerdocyon thous*	78	0	0.0
Canidae	*Canus lupus familiaris* (dog)	1588	319	20.1
Canidae	*Nasua nasua*	31	0	0.0
Chiroptera	*Eptesicus serotinus*	308	38	12.3
Chiroptera	*Miniopterus schreibersii*	47	1	2.1
Chiroptera	*Murina leucogaster*	2	0	0.0
Chiroptera	*Myotis formosus*	1	0	0.0
Chiroptera	*Myotis macrodactylus*	9	0	0.0
Chiroptera	*Plecotus auritus*	1	0	0.0
Chiroptera	*Rhinolophus ferrumequinum*	385	58	15.1
Chiroptera	*Vespertilio superans*	44	2	4.5
Cricetidae	*Alticola roylei*	21	4	19.0
Cricetidae	*Arvicola scherman*	64	2	3.1
Cricetidae	*Clethrionomys rufocanus*	81	10	12.3
Cricetidae	*Clethrionomys rutilus*	13	0	0.0
Cricetidae	*Cricetulus barabensis*	27	2	7.4
Cricetidae	*Cricetulus migratorius*	303	7	2.3
Cricetidae	*Cricetulus triton*	655	77	11.8
Cricetidae	*Cricetus* sp.	4	0	0.0
Cricetidae	*Hyperacrius fertilis*	31	5	16.1
Cricetidae	*Hyperacrius wynnei*	11	2	18.2
Cricetidae	*Lagurus lagurus*	84	8	9.5

Cricetidae	*Microtus agrestis*	7	0	0.0
Cricetidae	*Microtus arvalis*	49	6	12.2
Cricetidae	*Microtus fortis*	375	33	8.8
Cricetidae	*Microtus maximowiczii*	33	1	3.0
Cricetidae	*Microtus montebelli*	252	81	32.1
Cricetidae	*Microtus pennsylvanicus*	?	0	-
Cricetidae	*Microtus subterraneus*	4	0	0.0
Cricetidae	*Myodes andersoni*	1	0	0.0
Cricetidae	*Myodes glareolus*	302	44	14.6
Cricetidae	*Myodes regulus*	160	16	10.0
Cricetidae	*Myodes smithii*	14	10	71.4
Cricetidae	*Oecomys mamorae*	25	0	0.0
Didelphidae	*Gracilianus agilis*	11	0	0.0
Didelphidae	*Monodelphis domestica*	4	0	0.0
Didelphidae	*Thylamys macrurus*	14	0	0.0
Echimyidae	*Clyomys laticeps*	8	0	0.0
Echimyidae	*Thrichomys fosteri*	77	0	0.0
Erinaceidae	*Echinosorex gymnura*	4	0	0.0
Erinaceidae	*Erinaceus amurensis*	4	0	0.0
Felidae	*Leopardus pardalis*	7	0	0.0
Herpestidae	*Herpestes javanicus*	1	0	0.0
Lagomorpha	*Ochotona roylei*	6	0	0.0
Marsupialia	*Didelphis albiventris*	1	0	0.0
Marsupialia	*Echymipera kalubu*	30	4	13.3
Marsupialia	*Isoodon macrourus*	196	33	16.8
Marsupialia	*Perameles nasuta*	29	0	0.0
Muridae	*Acomys wilsoni*	21	0	0.0
Muridae	*Apodemus agrarius*	11656	3704	31.8
Muridae	*Apodemus argenteus*	41	6	14.6
Muridae	*Apodemus flavicollis*	95	15	15.8
Muridae	*Apodemus peninsulae*	290	26	9.0
Muridae	*Apodemus speciosus*	2435	866	35.6
Muridae	*Apodemus sylvaticus*	82	2	2.4
Muridae	*Apomys datae*	?	0	-
Muridae	*Arvicanthis niloticus*	6	0	0.0
Muridae	*Bandicota bengalensis*	233	43	18.5
Muridae	*Bandicota indica*	2176	301	13.8
Muridae	*Bandicota savilei*	54	12	22.2
Muridae	*Bandicota* sp.	17	1	5.9
Muridae	*Berylmys berdmorei*	33	3	9.1
Muridae	*Berylmys bowersi*	204	99	48.5
Muridae	*Chiropodomys gliroides*	6	1	16.7
Muridae	*Chrotomys whiteheadi*	?	0	-
Muridae	*Gerbillus gleadowi*	27	0	0.0
Muridae	*Golunda ellioti*	4	0	0.0
Muridae	*Hapalomys delacouri*	1	0	0.0

Muridae	*Hydromys chrysogaster*	8	0	0.0
Muridae	*Lenothrix canus*	63	3	4.8
Muridae	*Leopoldamys edwardsi*	20	5	25.0
Muridae	*Lophuromys sikapusi*	1	0	0.0
Muridae	*Mastomys erythroleucus*	147	0	0.0
Muridae	*Mastomys natalensis*	8	1	12.5
Muridae	*Maxomys inas*	15	0	0.0
Muridae	*Maxomys moi*	4	1	25.0
Muridae	*Maxomys rajah*	277	97	35.0
Muridae	*Maxomys sp.*	5	4	80.0
Muridae	*Maxomys surifer*	306	130	42.5
Muridae	*Maxomys whiteheadi*	372	101	27.2
Muridae	*Melomys cervinipes*	173	16	9.2
Muridae	*Melomys littoralis*	37	5	13.5
Muridae	*Melomys lutillus*	180	12	6.7
Muridae	*Melomys sp.*	3	0	0.0
Muridae	*Meriones erythrourus*	30	0	0.0
Muridae	*Meriones libycus*	4	0	0.0
Muridae	*Meriones tamariscinus*	1	1	100.0
Muridae	*Micromys minutus*	30	8	26.7
Muridae	*Milardia kondana*	8	0	0.0
Muridae	*Milardia meltada*	33	1	3.0
Muridae	"Mouse"	455	10	2.2
Muridae	*Mus booduga*	17	2	11.8
Muridae	*Mus caroli*	310	53	17.1
Muridae	*Mus cervicolor*	31	0	0.0
Muridae	*Mus commisarius*	?	0	-
Muridae	*Mus cookii*	11	0	0.0
Muridae	*Mus fragilicauda*	1	0	0.0
Muridae	*Mus musculus*	1333	198	14.9
Muridae	*Mus platythrix*	42	0	0.0
Muridae	*Mus saxicola*	16	0	0.0
Muridae	*Mus* sp.	68	5	7.4
Muridae	*Nesokia indica*	69	8	11.6
Muridae	*Niviventer confucians*	41	5	12.2
Muridae	*Niviventer coninga*	3	1	33.3
Muridae	*Niviventer cremoriventer*	122	12	9.8
Muridae	*Niviventer fulvescens*	67	4	6.0
Muridae	*Niviventer niviventer*	12	2	16.7
Muridae	*Rattus andamanensis*	40	10	25.0
Muridae	*Rattus annandalei*	499	282	56.5
Muridae	*Rattus argentiventer*	1074	397	37.0
Muridae	*Rattus blandfordi*	33	2	6.1
Muridae	*Rattus bukit*	12	0	0.0
Muridae	*Rattus canus malaisia*	?	0	-
Muridae	*Rattus conatus*	58	6	10.3

Muridae	*Rattus concolor browni*	51	2	3.9
Muridae	*Rattus coxingi*	13	7	53.8
Muridae	*Rattus diardi*	2	0	0.0
Muridae	*Rattus everetti*	7	7	100.0
Muridae	*Rattus exulans*	1034	175	16.9
Muridae	*Rattus flavipectus*	1516	1025	67.6
Muridae	*Rattus fuscipes*	84	4	4.8
Muridae	*Rattus gestri*	4	0	0.0
Muridae	*Rattus huang*	1	0	0.0
Muridae	*Rattus leucopus*	14	2	14.3
Muridae	*Rattus losea*	2614	742	28.4
Muridae	*Rattus luzonicus*	40	0	0.0
Muridae	*Rattus mackenziei*	1	0	0.0
Muridae	*Rattus mindanensis*	291	68	23.4
Muridae	*Rattus mordax*	30	0	0.0
Muridae	*Rattus muelleri*	605	235	38.8
Muridae	*Rattus nitidus*	1	0	0.0
Muridae	*Rattus norvegicus*	6050	1049	17.3
Muridae	*Rattus panglima*	2	2	100.0
Muridae	*Rattus praetor*	1	0	0.0
Muridae	*Rattus rattoides*	48	8	16.7
Muridae	*Rattus rattus*	7228	1487	20.6
Muridae	*Rattus rattus diardii*	226	18	8.0
Muridae	*Rattus rattus frugivorus*	5	2	40.0
Muridae	*Rattus rattus rufescens*	16	1	6.3
Muridae	*Rattus rattus satarae*	23	2	8.7
Muridae	*Rattus rattus septicus*	9	1	11.1
Muridae	*Rattus rattus sladeni*	?	0	-
Muridae	*Rattus rattus thai*	201	44	21.9
Muridae	*Rattus remotus*	?	0	-
Muridae	*Rattus ruber*	1	0	0.0
Muridae	*Rattus sabanus*	2474	896	36.2
Muridae	*Rattus* sp.	394	17	4.3
Muridae	*Rattus tanezumi*	359	65	18.1
Muridae	*Rattus tiomanicus*	4117	984	23.9
Muridae	*Rattus turkestanicus*	3	3	100.0
Muridae	*Rhombomys opimus*	305	4	1.3
Muridae	"Rodent"	1270	93	7.3
Muridae	*Sicista concolor*	7	0	0.0
Muridae	Swiss albino mouse	?	0	-
Muridae	*Tatera indica*	98	8	8.2
Muridae	*Uromys caudimaculatus*	47	7	14.9
Muridae	*Zapus hudsonius*	58	0	0.0
Mustelidae	*Melogale personata*	7	1	14.3
Reptilia	"Lizards"	51	0	0.0
Reptilia	*Physignathus lesueurii*	8	0	0.0

Reptilia	*Varanus* sp.	1	0	0.0
Reptilia	*Varanus varius*	1	0	0.0
Sciuridae	*Callosciurus caniceps*	46	4	8.7
Sciuridae	*Callosciurus erythraeus*	1	0	0.0
Sciuridae	*Callosciurus flavimanus*	1	0	0.0
Sciuridae	*Callosciurus nigrovittatus*	241	17	7.1
Sciuridae	*Callosciurus notatus*	791	14	1.8
Sciuridae	*Callosciurus prevostii*	18	0	0.0
Sciuridae	*Callosciurus* sp.	4	0	0.0
Sciuridae	*Dremomys rufigens*	1	0	0.0
Sciuridae	*Funambulus pennantii*	9	0	0.0
Sciuridae	*Funambulus tristriatus*	5	0	0.0
Sciuridae	*Hylopetes spadiceus*	125	0	0.0
Sciuridae	*Iomys horsfieldii*	2	0	0.0
Sciuridae	*Lariscus insignis*	2	0	0.0
Sciuridae	*Marmota baibacina*	58	4	6.9
Sciuridae	*Marmota bobak*	26	0	0.0
Sciuridae	*Menetes berdmorei*	21	2	9.5
Sciuridae	*Menetes* sp.	30	0	0.0
Sciuridae	*Petinomys setosus*	2	0	0.0
Sciuridae	*Pteromyscus pulverulentus*	7	0	0.0
Sciuridae	*Spermophilopsis leptodactylus*	2	0	0.0
Sciuridae	*Sundasciurus brookei*	2	0	0.0
Sciuridae	*Sundasciurus hippurus*	1	0	0.0
Sciuridae	*Sundasciurus lowii*	22	0	0.0
Sciuridae	*Sundasciurus tenuis*	26	0	0.0
Sciuridae	*Tamias sibiricus*	249	64	25.7
Simiformes	*Macaca fascicularis*	27	12	44.4
Soricidae	*Crocidura attenuata*	11	0	0.0
Soricidae	*Crocidura dsinezumi*	2	0	0.0
Soricidae	*Crocidura horsfieldii*	1	0	0.0
Soricidae	*Crocidura lasiura*	279	6	2.2
Soricidae	*Crocidura olivieri*	2	0	0.0
Soricidae	*Crocidura* sp.	25	0	0.0
Soricidae	*Crocidura suaveolens*	25	1	4.0
Soricidae	*Neomys fodiens*	2	0	0.0
Soricidae	"Shrews"	62	0	0.0
Soricidae	*Sorex caecutiens*	?	0	-
Soricidae	*Sorex minutus*	2	0	0.0
Soricidae	*Suncus murinus*	836	158	18.9
Talpidae	*Talpa mogra*	3	0	0.0
Talpidae	*Urotrichus taipoides*	10	3	30.0
Tupaiidae	*Tupaia belangeri*	11	6	54.5
Tupaiidae	*Tupaia glis*	200	43	21.5
Tupaiidae	*Tupaia gracilis*	8	0	0.0
Tupaiidae	*Tupaia minor*	17	0	0.0

Tupaiidae	*Tupaia* sp.	11	0	0.0
Tupaiidae	*Tupaia tana*	86	0	0.0
Viverridae	*Paradoxurus hermaphroditus*	5	0	0.0
Viverridae	*Viverricula indica*	1	0	0.0
	Multiple listed	4643	1480	31.9
	Not Identified	15413	3169	20.6

Additional file 1: Table S7. Total and median tested and testing *O. tsutsugamushi* positive for the 5 most frequently tested vertebrate species, subdivided by laboratory test category

Lab method	Host species name	Total Tested	Median tested	Min	Max	Total positive	Median positive/study site	Median positive (%)	Minium (%)	Max (%)
**Combination culture & molecular**	*Rattus norvegicus*	224	112	12	212	38	19	20.5	16	25
	Apodemus agrarius	30	15	3	27	13	4	30	0	33
	Bandicota indica	15	15	15	15	8	2	0	0	13
	Rattus rattus	15	15	15	15	1	0.5	0	0	0
	Cricetulus triton	13	13	13	13	5	5	38	38	38
**Combination culture & serology**	*Rattus rattus*	5626	284.5	12	1643	1316	21	16	0	100
	Bandicota indica	1561	75	1	885	127	17	12	0	29
	Apodemus speciosus	1435	9	1	263	438	2	20	0	100
	Rattus losea	1335	163	49	411	213	29.5	22	0	30
	Rattus tiomanicus	1171	585.5	234	937	54	27	10.5	0	21
**Culture +- microscopy**	*Rattus tiomanicus*	1503	115.5	1	303	355	24.5	24	0	30
	Rattus sabanus	1084	72	2	362	251	19.5	18.5	0	60
	Rattus argentiventer	497	23.5	0	150	182	7	34	0	100
	Rattus exulans	377	6	0	152	56	1	0	0	50
	Apodemus agrarius	296	8.5	3	109	38	2	13	0	86
**Serological**	*Apodemus agrarius*	8991	19	1	1094	3250	4.5	22.5	0	88
	Rattus tiomanicus	1413	2	1	327	573	1	26	0	100
	Dog (*Canis l. familiaris*)	1523	20	6	238	307	5.5	24.5	0	84
	Rattus sabanus	1375	14.5	2	748	644	4	30.5	0	63

	Rattus flavipectus	1081	21.5	7	769	870	6	44	4	100
**Molecular**	*Rattus norvegicus*	2414	25	1	1261	113	1	2	0	57
	Apodemus agrarius	1410	16	2	370	304	3	27	0	88
	"Rodent"	927	230	201	266	60	12.5	5	4	12
	Mus musculus	557	20	1	183	25	1	5	0	100
	Sheep (*Ovis aries*)	510	255	210	300	16	8	3.5	3	4

Additional file 1: Table S8. Median chigger index (mean number of chiggers per host species) for species where reported.

Species	Median chigger index
Apodemus agrarius	78
Apodemus argenteus	89
Apodemus peninsulae	8
Apodemus speciosus	53
Bandicota bengalensis	22
Bandicota indica	54
Bandicota savilei	58
Berylmys berdmorei	28
Callosciurus erythraeus	0
Gallus gallus domesticus (chicken)	2
Clethrionomys rufocanus	29
Clethrionomys rutilus	16
Cricetulus triton	76
Crocidura lasiura	1
Funambulus tristriatus	17
Golunda ellioti	11
Micromys minutus	15
Microtus fortis	0
Milardia kondana	240
Milardia meltada	131
Mus booduga	5
Mus caroli	2
Mus musculus	1
Mus platythrix	7
Myodes smithii	180
Niviventer confucians	26
Niviventer coninga	0
Niviventer fulvescens	17
Rattus argentiventer	170
Rattus blandfordi	18
Rattus coxingi	14
Rattus exulans	8
Rattus flavipectus	85
Rattus losea	51
Rattus mindanensis	4
Rattus norvegicus	50
Rattus rattus	45
Rattus rattus diardii	400
Rattus rattus rufescens	47
Rattus rattus satarae	130
Rattus remotus	8
Rattus tanezumi	394

Rattus tiomanicus	78
Sorex caecutiens	0
Sorex minutus	0
Suncus murinus	29
Multiple listed	92
Not identified	74

Additional file 1: Table S9. Median percentage infestation rate (percentage of hosts with at least 1 chigger attached) for species where reported.

Species	Median percentage infestation
Apodemus agrarius	61
Apodemus speciosus	14
Bandicota bengalensis	25
Bandicota indica	95
Bandicota savilei	100
Berylmys berdmorei	67
Callosciurus erythraeus	0
Clethrionomys rufocanus	74
Clethrionomys rutilus	77
Cricetulus triton	89
Crocidura lasiura	4
Echymipera kalubu	100
Funambulus tristriatus	42
Golunda ellioti	56
Isoodon macrourus	47
Melomys cervinipes	61
Melomys lutillus	45
Menetes berdmorei	88
Microtus fortis	0
Milardia kondana	96
Milardia meltada	100
Mus booduga	38
Mus caroli	26
Mus musculus	15
Mus platythrix	28
Myodes regulus	100
Neomys fodiens	0
Niviventer confucians	48
Niviventer coninga	50
Niviventer fulvescens	41
Rattus argentiventer	99
Rattus coxingi	27
Rattus exulans	33
Rattus flavipectus	89
Rattus leucopus	47
Rattus losea	93
Rattus mindanensis	25
Rattus norvegicus	46
Rattus rattus	56
Rattus rattus diardii	41
Rattus rattus rufescens	64

Rattus rattus satarae	92
Rattus remotus	50
Rattus sp.	62
Rattus tanezumi	100
Rattus tiomanicus	96
Sorex caecutiens	0
Sorex minutus	0
Suncus murinus	59
Tatera indica	0
Tupaia glis	100
Uromys caudimaculatus	47
Not Identified	17
